# Health net-outcome objectives and approaches for spatial planning and development: a scoping review

**DOI:** 10.11124/JBIES-25-00058

**Published:** 2026-12-10

**Authors:** James Stewart-Evans, Emma Wilson, Tessa Langley, Angela Hands, Karen Exley, Jo Leonardi-Bee

**Affiliations:** 1 Nottingham Centre for Public Health and Epidemiology, University of Nottingham, Nottingham, UK; 2 Environmental Hazards and Emergencies Department, UK Health Security Agency, UK; 3 Nottingham JBI Centre for Evidence-Based Healthcare, Nottingham, UK; 4 Office for Health Improvement and Disparities, Department of Health and Social Care, UK

**Keywords:** health, net gain, no net loss, policy, spatial planning

## Abstract

**Objective:**

The objective of this scoping review was to map the body of knowledge on net gain and no-net-loss (net-outcome) objectives and approaches applicable to health in spatial planning and development policies and practice.

**Introduction:**

There is an established body of academic and gray literature addressing environmental net-outcome objectives, such as biodiversity net gain, in spatial planning policies and practice. While a health net-gain objective has been proposed as a driver for health protection and the realization of health, such an objective and approach are yet to be scoped and defined.

**Eligibility criteria:**

This review considered scientific and gray literature sources that described health net-outcome objectives and approaches that can be implemented in spatial planning and development policies and practice. Source contexts were not limited to specific countries, geographical areas, or settings. All types of evidence were considered.

**Methods:**

This review followed the JBI methodology for scoping reviews. Searches of 19 information sources were conducted in August 2023 and updated in July 2024. Key databases included Scopus, MEDLINE, and Embase. Sources of gray literature were included, and citation searching was conducted. No language or date restrictions were applied. Following a high level of agreement during piloting, titles and abstracts were screened by 1 reviewer, and 50% of full texts were screened by 2 reviewers. One reviewer extracted data describing the characteristics of evidence sources and the net-outcome objectives and approaches described within them. Data analysis included categorization, frequency counts, and a SWOT (strengths, weaknesses, opportunities, threats) analysis.

**Results:**

Of 8290 unique records identified through database and gray literature searching, 474 evidence sources were assessed for eligibility, resulting in the inclusion of 112 sources, alongside 7 others identified from citation searching, for a total of 119 sources. Included evidence sources dated from 1974 to 2024, with an increasing frequency of publication from 2008. Social objectives were found from the 1990s, and conservation policies engendered well-being objectives from 2018. Frequently encountered perspectives related to regenerative and sustainable design and development, biodiversity, and conservation. Almost all sources originated from developed Western economies. Broad objectives relevant to health (90/119) outnumbered distinct health objectives (29/119). Most sources addressed development projects, among other scales. Sources frequently described the reconceived use of development to protect and improve health and well-being, overcome sustainability challenges, and strengthen socioecological systems. Implementation often featured participatory approaches, mitigation hierarchies, and assessment, although some sources advocated positive opportunities for health creation rather than the use of contested quantitative accounting frameworks. Challenges and opportunities were predominantly associated with objective specification and assessment. Potential value conflicts were identified relating, in part, to differing anthropocentric and biocentric approaches and objections to quantification and commodification.

**Conclusions:**

This review found many socio-environmental net-outcome objectives relevant to health and emergent health objectives that were immature and less frequently reported. These present differing scopes, focuses, and implementation options that are relevant to policymakers’ specification of future objectives. Specification entails value judgments, and equity considerations are important. Knowledge gaps to address include transferability between countries, policy domains, and disciplines; multilevel evaluation; and integration within spatial planning systems and current impact assessment theory and practice.

**Review registration:**

OSF https://osf.io/4dbcm

## Introduction

The longstanding and ubiquitous objective of minimizing costs and maximizing benefits is illustrated by a founding Hippocratic principle of medical ethics—produce net health benefits with minimal harm^1^—and established economic analyses of societal costs and benefits that influence regulation and decision-making.^2^-^5^ The specific costs and benefits in question in any given policy context depend on policymakers’ priorities and interests, and can include consideration of the implications of changes to our built and natural environments; aspects of individual health; and costs and benefits to nature, the health care system, or wider economy.

Spatial planning is a socio-spatial and integrative process that shapes present and future places through visions, actions, implementation strategies, and coproduction.^6^ Global goals and principles for socioeconomic development are articulated by international initiatives such as the UN Sustainable Development Goals and New Urban Agenda.^7^,^8^ Spatial planning policies are incrementally localized through national to local government policies that influence the organizational policies and actions of the private and public sectors.^9^ The implications of development and changes in land use are typically subject to assessment, both at a strategic policy level and at the level of individual development projects.^9^,^10^

Health is one consideration for spatial planning policies and associated assessments because our built and natural environments are determinants of health.^9^,^11^,^12^ The traditional land-use planning model focuses on regulating and ordering the use of land, aiming, inter alia, to protect nature and human health from harms associated with inappropriate and unconstrained economic development and poor environmental conditions.^9^,^13^-^15^ Contemporary spatial planning policies may temper optimization of economic efficiency with environmental constraints or equity objectives.^13^ This is reflected by the broader objectives of sustainable development: balancing economic, social, and environmental objectives and seeking net gains across them,^7^,^16^,^17^ increasing the emphasis on social and environmental outcomes.

Environmental net-outcome policy objectives set specific targets for measurable aspects of the natural environment.^18^ Net zero is a prominent contemporary neutrality policy objective applied to carbon emissions. Neutrality objectives also feature in conservation: no net loss of wetlands has been a policy objective in the USA since the 1970s,^19^ while biodiversity no-net-loss (NNL) objectives have proliferated internationally in recent decades.^19^,^20^ Net gain (NG) objectives within environmental and spatial planning policies extend the ambition of preceding NNL policies, requiring demonstrable gains in target outcomes.^18^,^21^ A wide range of environmental pressures and outcomes can be targeted, as illustrated by the application of NG objectives to natural capital and ecosystem services essential to human health.^22^,^23^ The potential for environmental net-outcome policies to affect social outcomes (either negatively or positively) has spurred the development of secondary social net-outcome objectives and arguments for policy designs that benefit nature and people.^24^-^30^ Overarching ambitions for socioecological NG are similarly expressed in literature that argues for a transition beyond sustainable to regenerative development: development that, in essence, does more good for people and the planet.^31^-^35^

The premise of minimizing harms and maximizing health benefits is, in itself, not new: spatial planning policy and practice already incorporate some of the aforementioned socioecological objectives and offer an established framework for the protection and improvement of nature and human health^9^,^24^,^31^,^36^-^38^; however, development can lead to damage despite the existence of protections.^11^,^39^ Although spatial planning policies typically address health, it is usually within the context of the broader economic, social, and environmental objectives of sustainable development. Health is one of many competing considerations for spatial planners, and the proponents of development for whom health matters are not always a priority^11^,^40^; planning policies may prioritize economic growth and set housebuilding targets to be delivered by cost-sensitive private-sector developers. Enduring challenges to health creation are illustrated by the persistence of spatial inequalities that include worse built and natural environmental quality and poorer health in more deprived areas.^41^

Addressing these entrenched challenges calls for approaches to sustainable urban development that systematically prioritize health and well-being.^8^,^42^-^44^ Recent advocates suggest that a health NG objective in spatial planning policy may be transformative and attractive to health policymakers,^45^-^49^ both as a means of prioritizing health in future development and of narrowing existing spatial inequalities through specific consideration of the distribution of harms and benefits at policy and development project levels. Although such an objective and approach have not been defined in this context, it has been suggested that recent environmental net-outcome policies and approaches are potentially transferable.^24^,^48^-^50^ Net-outcome objectives and approaches used in other health policy contexts (such as regulatory appraisal or risk regulation) are also potentially relevant, as are other broader objectives that specify social or socioecological net-outcome objectives relevant to health, such as community NG.^51^-^54^ Although the argument is made that health NG offers new opportunities to reprioritize health within the planning system as has been done for biodiversity, the literature describing socio-environmental net-outcome objectives also documents challenges and impacts associated with different policy designs and implementation models, from which lessons for prospective health policymakers can be collated.

A preliminary search of PROSPERO, the Cochrane Database of Systematic Reviews, *JBI Evidence Synthesis*, EPPI Database of Promoting Health Effectiveness Reviews (DoPHER), Collaboration for Environmental Evidence Database of Evidence Reviews, OSF, PubMed, Scopus, Web of Science, and Figshare was conducted. No current or in-progress scoping or systematic reviews were identified that addressed health NG or NNL of health in the context of spatial planning policy objectives or approaches.^55^

The overarching objective of this scoping review was to scope the body of knowledge addressing net-outcome objectives and approaches applicable to health in spatial planning and development policies and practice.^55^ Related objectives were to clarify health net-outcome concepts and conceptual boundaries, describe how health net-outcome objectives are implemented in practice and the associated opportunities and challenges, and identify knowledge gaps in theory and practice.^55^

## Review questions

- What are the characteristics of health net-outcome policy objectives and the approaches that implement them in practice, including the rationales for their existence and use, and definitions of the objectives?
- How do health net-outcome policy objectives and approaches define health, and what are the principles or requirements that govern their implementation?
- What is the contextual positioning of health net-outcome policy objectives and approaches, and what are their effects or implications, including implementation opportunities and challenges?

## Eligibility criteria

### Concept

The overarching concept of interest of the review was health net-outcome objectives that can be implemented in spatial planning and development policies and practice. Sources were included if they defined, described, or appraised health net-outcome objectives or approaches.

Health was primarily conceptualized as population health and well-being, but inclusion was not dependent on sources’ definitions of health, and sources directly referencing any form of health or well-being net-outcome objective were included. Sources describing broader societal, social, community, and people-oriented net-outcome objectives were eligible for inclusion (eg, objectives of social NG, community NG, or NNL of well-being). Sources describing health net-outcome objectives related to specific environmental determinants of health were included (eg, a health NG objective related to health outcomes associated with changes in air quality^48^).

Sources describing other net-outcome objectives (eg, environmental NG) were included if they addressed environmental change in broad terms (ie, changes in the built and natural environment affecting people and nature) or explicated the application of net-outcome objectives or elements of a net-outcome approach to social or health objectives. Otherwise, sources describing nature and ecological-oriented net-outcome objectives (eg, natural capital, ecosystem services, biodiversity NG) were included only if their main aim and focus was the appraisal or conceptual elaboration of net-outcome-type policy objectives in general, or if they drew transferable principles from specific approaches. Sources describing regenerative development and related concepts of systemic socioecological NG were excluded unless they detailed a specific net-objective’s definition and appraisal.

Net-outcome *objectives* encompassed goals, aims, targets, or requirements for, or principles of, NNL or an NG (or equivalently termed objectives). Sources describing *approaches* that implement net-outcome objectives in policies and practice were also included. The review’s concept of a net-outcome approach encompassed sources that described conceptual frameworks, theories, models, and definitions; underpinning ideological stances, implementation principles, and their implications; and associated assessment and delivery requirements. Mitigation hierarchies (a well-established framework designed to prevent and limit environmental harm through prioritized steps of harm avoidance, reduction or minimization, reversal or remediation, and offsetting^56^) were considered a feature of net-outcome approaches, and sources describing them were eligible for inclusion.

Sources that referred to health net outcomes in general terms without relating them to the core concept of a policy objective or approach (eg, an evaluation reporting an NG in health after an intervention) were excluded. Sources that described methodologies for the economic assessment of costs and benefits (in particular, cost-benefit analysis) were excluded unless they specifically addressed net-outcome policy considerations or the context of spatial planning.

### Context

Sources were included if they described objectives or approaches that were or can be implemented by, or were applicable to, spatial planning and development policies and practice.

The phrase *implemented by* encompassed sources describing spatial planning principles; spatial planning and development planning policies; spatial planning practice guidance; spatial plans; design codes; development plans; and assessments of the effects of development projects, spatial plans, or planning policies.

The phrase *can be implemented* encompassed sources that did not explicitly address spatial planning and development but addressed place-making or the planning and governance of changes in natural and built environments related to land use, development, building, and infrastructure provision.

The phrase *applicable to* encompassed sources that described or evaluated the abstract concept of net-outcome policy objectives or approaches (without applying them to a particular domain). Sources that addressed health net-outcome objectives or approaches in other domains of policy or practice were considered if they described or evaluated the overarching concept of net-outcome policy objectives or approaches, or referred to them in the context of development or spatial planning and development planning policies and practice.

Source contexts were not limited to specific countries, geographical areas, or settings.

### Types of sources

Sources of evidence included any type of evidence. The review included sources from both scientific and gray literature.

## Methods

This scoping review was conducted in accordance with the JBI methodology for scoping reviews,^57^ and reported in line with the Preferred Reporting Items for Systematic Reviews and Meta-Analyses extension for Scoping Reviews (PRISMA-ScR).^58^ The a priori protocol for the review^55^ is registered in OSF (https://osf.io/4dbcm).

### Search strategy

The search strategy was not limited by types of evidence source and aimed to locate published and unpublished studies, reviews, opinion papers, policy and briefing papers, reports, articles, transcripts, and any other relevant material. Searches targeted 3 domains: i) sources referencing the specific concept of health NG; ii) sources referencing health terms and net-outcome concepts; and iii) sources describing net-outcome concepts and features of policy objectives and approaches. To avoid inadvertent omission of sources describing net-outcome objectives in other transferable contexts, contextual relevance to spatial planning and development was determined during evidence selection.

An initial search of Scopus and MEDLINE (Ovid) was conducted to identify articles that referenced synonyms of the phrase *health net gain* or the terms *health* and *net gain*. The text words contained in the titles and abstracts of relevant articles, and the index terms used to describe them, were reviewed to identify and incorporate additional phrases and terms related to the 3 domains described previously, and to iteratively develop a full search strategy for MEDLINE via Ovid (Appendix I). The search strategy was replicated across all included evidence sources using, or adapting, all identified keywords and index terms, and a second search was undertaken on all databases from August 17, 2023. To update the review, a third search of the databases was undertaken from July 12, 2024. Simplified approaches were used for gray literature databases with limited search functionalities. Search strategies and dates are provided in Appendix I.

Forward and backward (1 level) reference searches of sources included in the review were used to identify additional sources. These were only conducted for included sources that described a stand-alone, primary health net-outcome objective (see row T1, Table 1).

**Table 1:** Types and groups of net-outcome objectives described

Type of net-outcome objective		Group
T1	A stand-alone health or well-being net-outcome objective	Distinct health net-outcome objectives
T2	A net-outcome objective with a subsidiary/secondary health or well-being net-outcome objective	
T3	A composite/multi-outcome net-outcome objective incorporating a specific health or well-being net-outcome objective	
T4	A stand-alone social or community net-outcome objective	Broader net-outcome objectives
T5	A net-outcome objective with a subsidiary/secondary social or community net-outcome objective	
T6	A composite/multi-outcome net-outcome objective relevant to or encompassing health	
T7	A generic net-outcome objective	
T8	Universal features of or considerations for net-outcome objectives (eg, mitigation hierarchies, offsetting, equity)	

Articles published in any language from database inception to the search dates were included (ie, no date restrictions were applied). Translations from languages other than English were sourced using DeepL (DeepL, Cologne, Germany). Due to the number of evidence sources, resource constraints, and research timelines, automated translations were not subject to further verification. The databases searched were APA PsycINFO (Ovid), Embase (Ovid), HMIC Health Management Information Consortium (Ovid), MEDLINE (Ovid), International Bibliography of the Social Sciences (ProQuest), Applied Social Sciences Index and Abstracts (ProQuest), PAIS Index (ProQuest), Political Science Database (ProQuest), Worldwide Political Science Abstracts (ProQuest), Social Science Database‎ (ProQuest), Sociology Database (ProQuest), Social Services Abstracts (ProQuest), Sociological Abstracts (ProQuest), Policy File (ProQuest), and Scopus.

Gray literature databases searched were TRIP Pro, ProQuest Dissertations and Theses (ProQuest), and BASE. Limited additional web searches of Google were conducted (using an adapted subset of journal database search strings and reviewing all hits per search).

### Source of evidence selection

Search results were imported into EndNote v.20.6 (Clarivate Analytics, PA, USA), de-duplicated following 2 software-specific methodologies,^59^,^60^ then transferred to Covidence (Veritas Health Innovation, Melbourne, Australia) for screening in line with the eligibility criteria. A combination of manual (EndNote) and automated (Covidence) duplicate detection was used after the updated search (in 2024) to remove results that duplicated sources found in the original search.

A pilot test of a sample of 30 titles and abstracts was performed to refine eligibility criteria and reviewers’ guidance prior to screening. The sample comprised 20 randomly selected sources and 10 purposively selected to represent different types and aspects of net-outcome objectives. Next, the titles and abstracts of the first 10% of evidence sources were screened independently by 2 reviewers (JSE and AH). As agreement was >90% (93%), the remaining titles and abstracts were screened by 1 reviewer (JSE). Due to the number of potentially eligible evidence sources, full-text screening was conducted independently by 2 reviewers (JSE and AH; 237/474, 50%, with 72% agreement); the remaining evidence sources were screened by 1 reviewer (JSE). Any disagreements that arose between the reviewers were resolved by consensus. Eligibility criteria permitted inclusion of evidence sources associated with the authors and the review itself. Full-text evidence sources that did not meet the eligibility criteria were excluded and are listed in Appendix II.

### Data extraction

Data were extracted from included evidence sources by a single reviewer (JSE) using a data extraction tool developed in Microsoft Excel v.2408 (Microsoft, Washington, USA; Appendix III), with a second reviewer (AH) double-checking a random sample of 10% of sources. Initially, the extracted content from 10 evidence sources was reviewed and used to generate lists of keywords relevant to the following data items: “net” terms, definitions and metrics, principles, strengths, weaknesses, opportunities, and threats. Remaining source documents were subsequently imported into ATLAS.ti V23-24 (Lumivero, CO, US), and paragraphs containing these keywords were tagged (highlighted) to assist identification of potentially relevant content for extraction.

The data extracted included evidence source details and specific details about the characteristics of health net-outcome policy objectives and approaches, and their contextual positioning and implications. Modifications to the protocol’s draft data extraction instrument^55^ are denoted by annotations in Appendix III and comprised the following:

- extraction of additional source details (author or database keywords, URLs)
- extraction of data differentiating between net-outcome objectives’ descriptions, definitions, and measurement
- supplementary categorization of extracted data describing types of net-outcome objectives
- supplementary categorization of source content (source perspectives, scales of objectives’ application).

Appendix III outlines categorical data items and reviewer guidance related to specific data items. Corresponding authors were not contacted for additional information or clarification. Any disagreements that arose between the reviewers were resolved through discussion.

In parallel, the use of a large language model (LLM) Claude 3.5 Sonnet (Anthropic, CA, US), was trialed by reviewer JSE to expedite data extraction and review as part of a study within a review (SWAR).^61^ The LLM and the review protocol were used to extract a subset of data items from the publicly available sources previously selected for human review. LLM data extraction was subsequently compared to the baseline human data extraction. Human data extraction (addressing all data items) was also separately reviewed by the LLM against the original content of sampled sources. The SWAR concluded that while data extraction could be expedited using an LLM, performance was subpar for complex data items. A second LLM review of baseline extraction proved unreliable. Consequently, LLM double-extraction or second review was not pursued beyond the trial sample. Detailed findings from this SWAR are beyond the scope of this manuscript and are reported separately.^61^

### Data analysis and presentation

To facilitate data analysis and presentation, text descriptions of net-outcome objectives were recoded into a standardized acronymic format (eg, HNG: health net gain) then categorized by type and grouped as i) distinct health net-outcome objectives or ii) broader net-outcome objectives (Table 1).

Categorical levels of operationalization of net-outcome objectives were assigned post-extraction after reviewing the data extracted for each source, as were (free-text) specific metrics or broader frameworks. Descriptive content analysis of findings was undertaken, including frequency counts of source characteristics and categorical variables that described net-outcome objectives’ implementation. Heterogeneous text excerpts describing net-objectives, and their contextual positioning and effects, were summarized using alternative techniques, as described hereafter.

A comparative SWOT (strengths, weaknesses, opportunities, threats) analysis was conducted by aggregating extracted data for each SWOT element of each group of sources for analysis in ATLAS.ti. A concept search was carried out on each element to generate a list of concepts, associated noun phrases, and frequencies (see Supplemental File 1: http://links.lww.com/SRX/A150). Concept nouns were ordered by highest to lowest frequency, and each concept was reviewed (considering associated noun phrases and originating source text contexts) to confirm that it was a relevant SWOT concept (as detailed in the data extraction instrument; Appendix III). If relevant, the concept was summarized using a single bullet point sentence that contained the verbatim concept noun and thematically reflected relevant component noun phrases and originating source text contexts. Concepts that reiterated or elaborated existing bullet points were merged with them (and the combination denoted), unless their associated noun phrases and source contexts emphasized new points, in which case a new bullet point was added to the list. This process was repeated until either all the listed concepts had been reviewed or 5 concepts had been summarized.

This concept search and summary process was repeated for other data extracted as extensive free-text (objective rationale, objective description, net definition, and implementation principles) until 5 concepts had been summarized for each of these 4 data items for each of the 2 groups.

To identify potentially unique concept nouns, ATLAS.ti concept lists were tabulated and cross-referenced within Excel (Supplemental File 1: http://links.lww.com/SRX/A141). Firstly, previously presented frequent concept nouns were highlighted if they were unique within the dataset (ie, unique to a given data extraction target and group). Secondly, for each data extraction target, the 5 most frequently occurring nouns unique to each group (eg, strengths of health objectives that were not strengths of broader objectives) were listed (if count n>2), along with any recurring or notable contextualizing words from their component noun phrases.

As extracted data describing i) health terms and ii) specific metrics and frameworks were, respectively, limited and heterogeneous, their presentation was limited to narrative summary.

## Results

### Source of evidence inclusion

**Figure 1:**
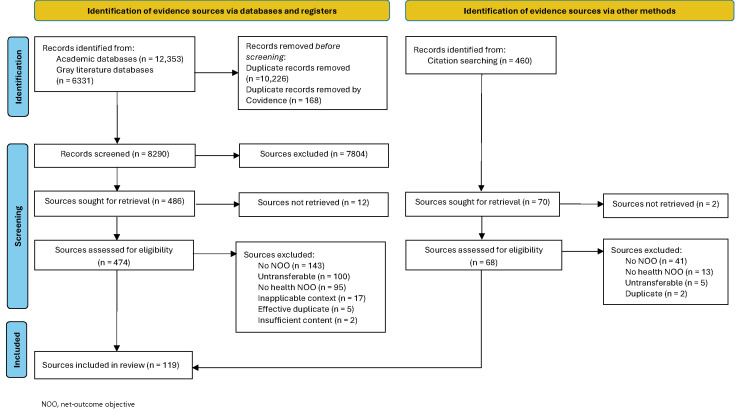
Search results and evidence source selection and inclusion/exclusion process^62^

The search strategy identified 18,684 records and, post-deduplication, 8290 unique records for screening (Figure 1,^62^). Title and abstract screening excluded 7804; therefore, 486 evidence sources were sought for retrieval, of which 12 were unavailable. Subsequently, 474 evidence sources were assessed for eligibility, of which 362 were excluded. The main reasons evidence sources were excluded on full-text examination were because they did not describe an explicit net-outcome objective, they did not describe an explicit health net-outcome objective, or the objective or approach described by the source was judged not transferable to other contexts. One exclusion reason was recorded for each excluded evidence source, although multiple exclusion reasons typically applied. Individual reasons for exclusion are summarized in Appendix II.

A further 7 evidence sources were identified from citation searching. Thus, a total of 119 evidence sources were included in the review.^4^,^5^,^16^-^20^,^22^-^37^,^45^-^56^,^63^-^146^

### Characteristics of included evidence sources

**Figure 2:**
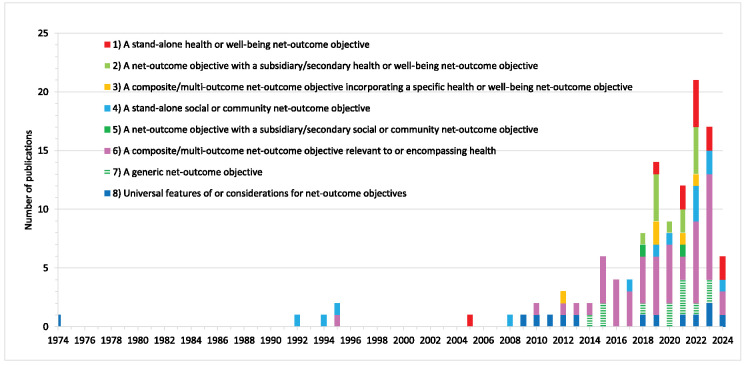
Source publication year and type of net-outcome objective described

The 119 evidence sources dated from 1974 to 2024 (Figure 2, Appendix IV, and Supplemental File 2: http://links.lww.com/SRX/A147), with the earliest sources describing social, community, and composite net-outcome objectives dating from the mid-1990s and the earliest stand-alone health objective dating from 2005. Several sources describing relevant net-outcome objectives were found each year from 2008–2014, after which there was a general upward trend in count per annum. Contributors to this trend comprised, firstly, a few additional sources each year describing generic net-outcome objectives; secondly, an increasing number of sources describing relevant composite net-outcome objectives; and thirdly, from the late 2010s, sources describing secondary and stand-alone health and social objectives.

**Figure 3:**
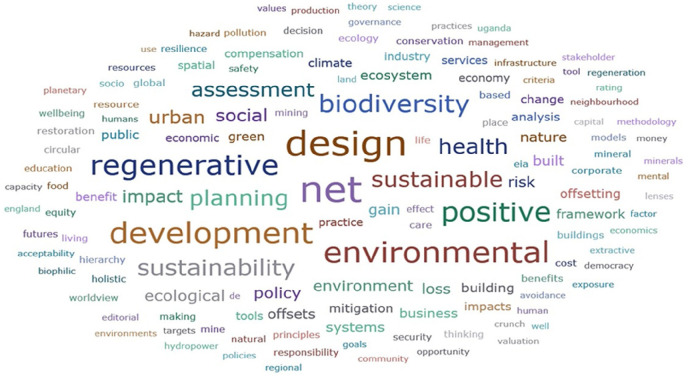
Word cloud of author keyword frequencies (where n>1) from included evidence sources

Figure 3 presents a visual summary of author keywords aggregated for all 119 evidence sources. Prominent keywords were associated with spatial planning and built and economic development (eg, design, assessment, urban, planning, development, sustainable, sustainability), net terms (eg, net, positive, gain, regenerative), the natural environment (eg, environmental, biodiversity, ecological), and health and social outcomes (eg, health, social). Extracted keywords were diverse and related to concepts, specific components of policies and practice, outcomes, spatial scales, stakeholders, and specific issues and considerations. Gains and losses were both addressed (keywords included benefit, gain, loss, cost). The most frequently encountered keywords related to regenerative and sustainable design and development. *Health* was referenced more often than *social*. Keywords addressing the environment (and biodiversity) were more frequent than health or social terms, which were more frequent than economic terms (Figure 3).

Table 2 provides a detailed breakdown of the characteristics of the included sources. These are grouped by the type of net-outcome objective described to facilitate the presentation of results. Tables S1 and S2 in Supplemental File 3 (http://links.lww.com/SRX/A148) also present selected characteristics grouped by source perspectives and countries of origin. Drawing from these tables, source characteristics are described firstly overall (characteristics of all 119 sources), then by type of net-outcome objective (T1–T8; Table 2).

**Table 2:** Characteristics of included sources, grouped by the type of net-outcome objective described

**Figure:** 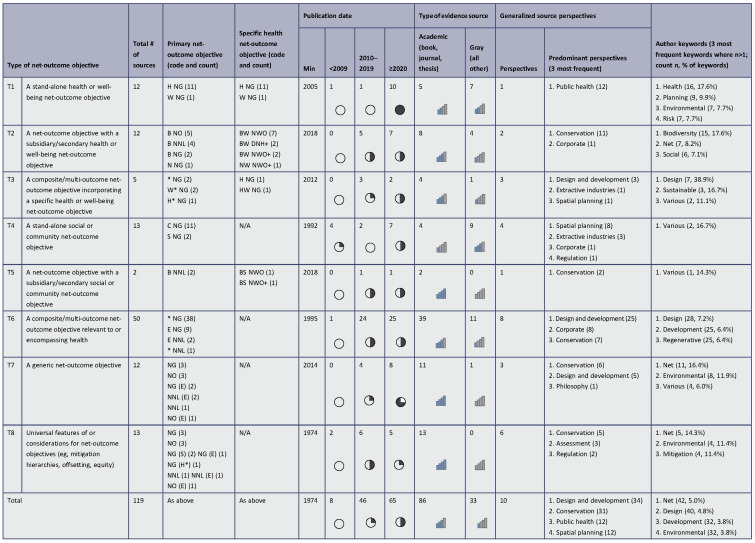

Key to codes abbreviating net-outcome objectives’ targets and outcomes in columns presenting primary and specific health net-outcome objective:

Targets: *, multi-objective; B, biodiversity; C, community; E, environmental; H, health; N, nature; S, social; W, well-being.

Outcomes: DNH+, do no harm or better off; NG, net gain; NNL, no net loss; NO, net outcome; NWO, no worse off; NWO+, no worse off or better off. Outcomes precede targets (in brackets) for generic net-outcome objectives (T7) and universal features (T8).

Harvey balls (round symbols; 5 quarters) and vertical bar icon sets (5 ratings) represent each cell value within quintile bands of row total numbers of evidence sources.

A complete version of this table is available in Supplemental File 4: http://links.lww.com/SRX/A149

Included sources predominantly dated from 2020 (65/119) or 2010–2019 (46/119). Eighty-six of 119 sources were academic (books, journals, or theses), with a comparatively higher proportion of gray literature sources among stand-alone social (type T4, 9/13), health (T1, 7/12), and secondary health (T2, 4/12) objectives (Appendix IV and Supplemental File 2: http://links.lww.com/SRX/A147 provide a breakdown of types of evidence source). Overall, the most frequent target outcome was NG (92/119), with fewer evidence sources describing a target of NNL (14/119), or both (13/119). The most frequent specific primary objectives described were multi-outcome NG (40/119), community NG (11/119), and health NG (11/119). Design and development (34/119) and conservation (31/119) together comprised >50% of source perspectives, followed by public health perspectives (12/119, which were exclusive to sources describing stand-alone health objectives, T1) and spatial planning perspectives (12/119, which primarily related to sources describing stand-alone social objectives [T4, 8/12]).

Word counts of extracted document aims indicated that *net* was the word most frequently mentioned across the 119 sources (47 counts, 2.4% of words). Other more frequently mentioned words varied, with none comprising a high percentage (>1.7%) of extracted words. Countries of origin were almost exclusively (112/119) advanced economies (as defined by the International Monetary Fund) from Europe, North America, or Australasia, as were countries of policy application (UK 18/119, Australia 9/119), although the review considered most objectives described to be universally applicable (87/119). The scales of application most often addressed by sources were the project level (85% across all sources, ranging from 75–100% within types of net-outcome objective), and local/regional (61%, 40–92%) and national (53%, 23–83%) policy levels; the city level was a less frequent focus of policy application (20%, 0–40%).

#### Characteristics of sources describing specific types of net-outcome objectives

Sources describing universal features and considerations (T8, 13/119) derived these primarily from environmental and biodiversity-oriented contexts. Mitigation was a notable author keyword focus. Dominant perspectives reflected conservation (5/13), assessment (3/13), and regulation (2/13) and international to local policies with a project-level application. Similar environmental and conservation focuses were displayed by sources describing generic net-outcome objectives (T7, 12/119), although these included design and development perspectives (5/12) and more frequently emphasized organizational and international, rather than national or sub-national, policy scales.

Composite, multi-outcome objectives (T6) were the type of net-outcome objective most frequently described by sources (50/119). Perspectives comprised primarily design and development (25/50), together with corporate (8/50) and conservation (7/50). Author and document-aim keywords reflected a common focus on sustainable or regenerative development, emphasizing building, project and system scales of application.

In an additional 5/119 cases, composite, multi-outcome objectives incorporated a specific health objective (T3). Perspectives included design and development (n=3), extractive industries (n=1), and spatial planning (n=1); restoration was a notable document-aim keyword. All 5 sources addressed the project scale, with (although noting only 5 sources in this group) more emphasis on the international and organizational policy scales than composite outcomes without a specific health objective (T6).

Social net-outcome objectives (T4, 13/119) were distinguished by a higher proportion of earlier publication dates (4/13 <2009) in comparison to other types of objective and perspectives from spatial planning (8/13) and extractive industries (3/13). These sources originated mainly from Australia (9/13) and Canada (3/13), describing objectives of social (2/13) and community NG (11/13) with a distinct focus on project-level application and local or regional policy.

Secondary health (T2, 12/119) and social (T5, 2/119) objectives were primarily associated with biodiversity net-outcome objectives (13/14) described by authors from the UK (11/14). While T2 sources described specific well-being objectives, the keyword *social* was also emphasized in their author and document-aim keywords. There was a distinct focus on application at the project (rather than building, city, or system) level and both spatially and organizationally oriented policies.

Most sources describing stand-alone health net-outcome objectives (T1) were published from 2021 by UK authors (10/12) representing public health perspectives (12/12). Due in part to publication and search phasing, these included the review protocol itself and related publications addressing the application of net-outcome objectives to health in spatial planning, emphasizing potential application in the UK context (see individual sources detailed in Appendix IV and Supplemental File 2: http://links.lww.com/SRX/A147). Notable author keywords included *risks* and *environmental, health*, and *planning*.

### Review findings

#### Net-objective and approach: characteristics

Table 3 presents the characteristics of distinct health (n=29, types T1–T3) and broader (n=90, types T4–T8) net-objectives and their approaches. Sources describing broader objectives often referred to the need to overcome sustainability challenges, particularly climate change, by moving targets from neutrality or harm minimization to positive outcomes for socioecological systems. Sources describing health objectives emphasized human health and well-being as narrower target outcomes; some described the specific need to link biodiversity policies to their social outcomes. These concepts were reflected in both rationales and descriptions of objectives, which typically applied to economic and built development. Health objectives frequently referenced the project level, while broader objectives included unique concept words related to corporate objectives and activities.

**Table 3:** Characteristics of net-objectives and approaches, grouped by types of net-outcome objective described

Data extraction target	Grouped objectives	Frequent concepts (first 5, contextualized)	Potentially unique concept words* (first 5, n>2)
Rationale(s) for net-outcome objective(s)	Distinct health	• Prioritising the protection and targeted improvement of **health** (#34/12) and **well-being** (#15/9)	• crisis (#5): *energy, economic, climate, multiple, preparedness*
	(T1–T3)		
	(28 of 29 sources)	• Linking changes in **biodiversity** (#22/9) to social impacts and **people**’s (#22/10) values, health, and well-being	• aim (#3): *overarching, strategic*
			• EU (#3): *policymaking*
		• Realizing environmental and health benefits through sustainable economic, urban, and housing **development** (#18/10)	
	Broader	• Overcoming **sustainability** (#24/17) challenges through reconceived **development** (#35/25) paradigms that move beyond minimization of negative **impact**s (#20) to demonstrably “do more good”	• climate (#11): *change, crisis, neutrality, restoration, advocates*
	(T4–T8)		
	(77 of 90 sources)	• Addressing, maintaining, and strengthening integrated socio-ecological **systems** (#22/17)	• decade (#7): *past, recent, next*
		• Broadening or specifying the scope of concepts, goals, or approaches used in environmental or spatial planning **policy** (#21/14)	• ambition (#5): *government, EU*
			• discourse (#5): *motivating positive, regenerative*
			• emission (#5): *carbon, greenhouse gas*
	Description(s) of net-outcome objective(s)	Distinct health	• Delivering **health** (#24/13) or well-being net-outcomes via new, or changes to existing, **development** (#28/11) or development **project**s (#28/10)	N/A
(T1–T3)				
(29 of 29 sources)		• Maintaining or improving health or well-being via **biodiversity** (#25/9) policies, projects, activities, or offsets		
		• Delivering health net **gains** (#24/16) or ensuring affected people are no worse off post-development		
Broader		• Delivering community, ecological, environmental, or social net **gain** (#34/24) through sustainable economic or built **development** (#37/30)	• net positive (#7): *goal*
(T4–T8)			• hurdle (#6): *footprint, goal*
(84 of 90 sources)		• Avoiding, minimizing or mitigating negative impacts and preferably going beyond compensation or offsetting (ie, beyond a no net **loss** [#24/18] criterion) to deliver wider, synergistic net positive **impacts** (#33/24) or **benefits** (#26/21)	• win (#6): *win-win approach*
			• asset (#5): *natural capital, natural, environmental*
			• chain (#4): *supply, customer, value*
Net-outcome definition/characterization and/or metric(s)	Distinct health	• Characterizing changes in specified **people’**s (#51/10) **well-being** (#64/10) using qualitative and quantitative indicators, addressing well-being’s different dimensions (material, subjective, and relational), individual (disaggregated) components, and spatiotemporal scales	• income (#7): *household*
	(T1–T3)		• success (#5): *measure*
	(21 of 29 sources)	• Characterizing changes in specified people’s **health** (#46/11), addressing health’s various aspects (eg, determinants of health; health needs and priorities; physical, mental, and population health outcomes) and spatiotemporal scales	• World Bank (#5): *third phase, funded projects*
		• Characterizing impacts on people specifically associated with changes in **biodiversity** (#48/10); biodiversity-related needs, values, and activities; and ecosystem service provision	• habitat (#3)
		• Characterizing baselines and trends and comparing health and well-being pre- and post-**development** (#42/13)	• situation (#3): *pre-project, their, a given*
	Broader	• Characterizing **development’s** (#87/31) positive and negative environmental, social, and/or economic **impacts** (#123/37), considering historic impacts and trends; cumulative impacts; the scales, acceptability, and significance of impacts; and the balance across a spectrum of impacts and spatiotemporal scales	• estate (#16): *public*
	(T4–T8)		• base (#15): *ecological, case*
			• basis (#14): *regional, biophysical, community-wide, area-wide, quantitative, consistent, rigorous, proper*
	(78 of 90 sources)	• Defining net **gains** (#75/28) or **benefits** (#87/32), inter alia, either as an unfixed decision criteria (ie, ultimately a context-dependent matter for communities or decision-makers) or through assessment of processes and/or outcomes (using qualitative or quantitative measures, including criteria-based tests, scoring, or cost-benefit accounting exercises)	• expectancy (#12): *life, health*
		• Characterizing baselines and effects with reference to inter-dependent human and natural **systems** (#77/25) and their current and future capacities to support life	• resilience (#12): *ecological, ecosystem, flood*
Implementation principle(s) and/or steps	Distinct health	• Addressing and involving defined groups of **people** (#138/19)— potentially focusing on significantly-affected, poor, or marginalized groups within a project’s area of influence—incorporating principles of distributional, procedural, and recognition equity, and recognizing that stakeholder groups and considerations change over time	• consultation (#17): *close, comprehensive, equitable, participatory, planning*
			• pap (#13): *different, individual, experiences, indirectly affected*
	(T1–T3)		
			• aggregation (#12): *units, groups*
			• choice (#12): *experiments, maintenance and operational*
		• Aligning or reconciling **biodiversity** (#129/13) and social agendas, objectives, considerations, approaches, and stakeholders	
	(28 of 29 sources)		
		• Embedding the strategic aim of improving **health** (#102/15) and **well-being** (#115/11) in national and local spatial planning policy and design codes and operationalizing it in policy, place and project-level design, assessment, and decision-making	
			• expert (#10): *social, biodiversity, independent, suitably qualified, judgements*
		• Addressing negative **impacts** (#115/17) within a mitigation hierarchy: preventing, avoiding, and minimizing harms and compensating or offsetting unavoidable residual impacts only as a last resort	
	Broader	• Addressing negative **impacts** (#330/55) within a mitigation hierarchy: preventing, avoiding and minimizing harms and compensating or offsetting unavoidable residual impacts only as a last resort	• boundary (#33): *system, spatial, planetary, temporal, growth, site, project, building, hard, reasonable, impact*
	(T4–T8)		
	(88 of 90 sources)		
		• Multiplying, maximizing or directing positive impacts and **benefits** (#227/44), including through policy recognition of opportunities, synergies and wider or co-benefits; provision of public goods; or targeting of benefits to reduce inequalities	
		• Operationalizing net-outcome objectives through holistic **development** (#214/55) policies, regulations, standards, plans, and processes (including design, assessment, and decision-making)	
		• Adopting new worldviews and **systems** (#187/49) paradigms to effect change across systems	
		• Conceptualizing **projects** (#163/38) as collaborations for positive change, seeking enduring flows of benefits	

EU, European Union; pap, project-affected persons.

* Unique across all targets = unique by comparison to all data extraction targets presented in Tables 3, 6, and 7 for both groups. Recurring or notable words in component noun phrases are presented in italics to aid contextualization.

Note: underlined frequent concepts denote a concept that was also unique between groups = unique to this data extraction target by comparison with the other group.

Note: 2nd column: n1 of n2 sources = number of sources for which data extraction is not blank, “N/A”, “Unstated” or “Aggregated (not extracted)”/total number of sources in that group.

Note: 3rd column: (#n3/n4) = count of concept word frequency/number of separate sources referencing the concept word.

Broad net-objectives’ definitions of NGs and benefits (Table 3) reflected a range of characterizations from unfixed criteria to assessed processes or outcomes. Baselines and effects were often described at the system level, with a particular emphasis on historic impacts and ecological resilience. In contrast, changes in the health and well-being of specified and grouped people pre- and post-development were a unique focus of health objectives, which referenced both socioeconomic (eg, income) and environmental (eg, biodiversity) determinants of health. Health terms were extracted from 18 of 29 sources describing health objectives (see Appendix IV and Supplemental File 2: http://links.lww.com/SRX/A147). References to “health” typically reflected the World Health Organization (WHO) definition of health as a “state of complete physical, mental and social well-being and not merely the absence of disease or infirmity.”^147^^(para.1)^ Similarly, references to “well-being”, which was typically the focal term, were often defined with reference to health as a component of well-being. Health and well-being terms were less frequently extracted from sources (10 of 90) describing broader objectives: most definitions addressed health, welfare, and well-being (as previous) similarly; however, health was defined by life expectancy in 2 cases, and 2 sources referred to a socioecological “health of the whole” or planetary health.

Both groups of objectives described the use of qualitative and quantitative measures and the importance of considering multiple impacts and scales (Table 3). Data extracted from 39 of 90 sources describing broader objectives referenced specific metrics or frameworks (see Appendix IV and Supplemental File 2: http://links.lww.com/SRX/A147). These were typically assessment frameworks: forms of cost-benefit analysis (n=6), environmental impact assessment (n=5), and United Nations Sustainable Development Goals (n=3) were most frequently referenced. Within text extracted from 10 of 29 sources describing health objectives, the conservation intervention well-being framework of Woodhouse *et al.*^148^ was most frequently noted (n=4).

Implementation principles described the operationalization of broader objectives from development policies to practice (Table 3): sources emphasized the role of collaborative projects and the importance of identifying opportunities and multiplying, maximizing, or directing benefits from the design phase onward. The adoption of new worldviews to effect change across systems was frequently described by broader objectives, with a particular focus on boundaries. Both groups of objectives referred to the application of a mitigation hierarchy or component steps (eg, avoid, minimize, mitigate, compensate, offset). Health objectives referenced the specific improvement of health and well-being as a strategic policy aim implemented throughout the planning system, highlighting the roles of consultations and decisions (choices) and the potential need to align or reconcile social and biodiversity objectives. People were frequently referenced with respect to considerations or principles of distributional, procedural, and recognition equity; the role of experts as stakeholders was also noted.

The review characterized the level at which net outcomes were emphasized by sources (Table 4, also see Tables S3 and S4 in Supplemental File 3: http://links.lww.com/SRX/A148). Twenty-eight sources focused on processes (level 2) such as implementation steps and procedural aspects of policies and practice (including the majority [9/13] of sources describing universal features and considerations). More sources described net outcomes as an overarching requirement (levels 1A and 1B, n=52) than an assessed outcome (levels 3A and 3B, n=39), with the highest proportion of the former describing stand-alone health objectives (overarching requirement: 9/12) and the highest proportion of the latter describing stand-alone social objectives (assessed outcome: 10/13). Of the sources describing overarching requirements, these were locally defined in a minority of cases (25%, 13/52), while of the sources describing assessed net outcomes, the majority (77%, 30/39) described determinations (eg, assessments or decisions) that were locally defined.

**Table 4:** Net-outcome level emphasized, grouped by the type of net-outcome objective described

**Figure:** 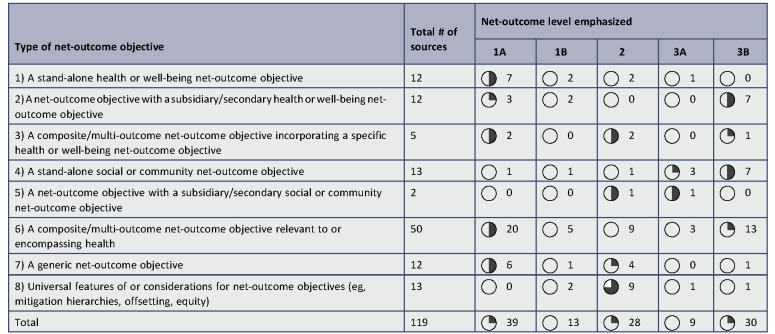

Net-outcome level emphasized: 1A, overarching requirement; 1B, overarching requirement (locally defined); 2, enhanced process; 3A, assessed outcome; 3B, assessed outcome (locally defined).

Harvey balls (round symbols; 5 quarters) represent each cell value within quintile bands of row total numbers of evidence sources.

#### Objective and approach: contextual positioning and effects

To contextualize and summarize relevant characteristics of net-outcome objectives and approaches described by evidence sources included in this review, Table 5 adapts and applies a framework used to develop and describe well-being net-objectives.^90^,^149^

**Table 5:** Characteristics of net-outcome objectives and approaches found by the review with reference to relevant issues and questions in net-outcome policy and practice

Issues in net-outcome policy and practice (adapted from Maron *et al.*^149^)	Perspectives from included evidence sources describing health and broader net-outcome objectives (summary commentary and example evidence sources)
What values are important?	Sources included in the review emphasized conservation and sustainability principles. Net-policies potentially reinforce market values^107^ and can create value conflicts related to contested valuations of nature or people’s well-being.^119^,^120^ Potential value conflicts were also noted between deep sustainability and humanism.^64^ The principles underpinning different assessment methodologies vary^5^,^104^ and embed value judgments relevant to decision-makers.^136^ Inclusive, transparent processes were recommended.^71^ Sources raised the importance of equity considerations,^30^,^66^,^104^ and suggested collaborative agreement of important values. Justice and social acceptability^4^ were other relevant considerations.^17^,^85^
Ethical basis	Some sources suggested that consideration of social, environmental, or economic benefits and costs was a moral obligation^28^,^124^ or matter of global security.^89^ There are ethical objections to accounting approaches that feature commodification and monetization.^19^,^119^ Aggregation of social good raises considerations of moral permissibility and questions of restrictions and human and individual rights.^95^ Sources variously described principles of utilitarianism (maximizing net benefits) and egalitarianism (narrowing disparities) and the setting of social (and ecological) safeguards.
Net outcome compared with what?	Reference scenarios must consider overarching goals (affected by multiple factors) and narrower impact-specific policy goals.^103^ Comparisons entail reasoned choices between fixed and dynamic reference points and consideration of pre- and post-development trends and redress of accumulated historic harms.
	Some sources characterized the realization of net-outcome objectives as not defined by the comparative quantification of outcomes and instead defined subjectively by decision-makers^63^ or with reference to following design processes and approaches that address capacities, flows, impacts, or positive opportunities.^91^,^118^,^125^
Net outcome of what?	Measurement can characterize diverse social, environmental, and economic determinants of health and aspects of health and well-being or narrow the focus to single monetary or other outcome measures, such as life expectancy.^49^,^52^,^90^ Environmental and natural capital net gain policies characterize environmental pressures, assets, and flows of benefits (ecosystem services) that influence human well-being.^23^,^100^ The use of both qualitative and quantitative methods was suggested by multiple sources with respect to multiple measures of people’s well-being. Well-being has material, subjective, and relational dimensions and encompasses objective and subjective elements related to human benefits and preferences. Well-being also has both causal and constitutive contributors.^119^
	In contrast to sources describing accounting approaches, some sources describing socioecological net gain emphasized mapping of the states of and interactions within complex systems and the qualitative social dimensions of transformational change.^122^,^138^ Local and global impacts are potentially relevant, as is the consideration of carrying capacities and thresholds.^127^,^142^
Net outcome for whom?	Building-design teams address future users, and project-level perspectives expand the focus to project-affected persons, communities, and project lifetimes.^25^ Assessment of net outcomes typically requires aggregation of individuals and definition of “appropriate” groups. Equity and scale are important when considering those potentially affected by development.^24^,^66^,^90^,^92^,^104^ The common context of sustainable development prompted consideration of intergenerational effects.^142^ Sources describing regenerative perspectives emphasized multi-scalar spatial and system-level framings.^86^,^115^,^122^
Applying the mitigation hierarchy	Mitigation hierarchies are established in environmental and planning policies,^133^,^141^ but net framing could undesirably predispose development towards compensation or offsetting of residual harms when this should be a last resort.^75^,^107^ One source suggested that net policies might accordingly separate the delivery of net outcomes from offset mechanisms.^107^
Capturing uncertainty and time lags	The latency of the effect of environmental changes on health is diverse, and attribution of causality is often uncertain (and may hinder explorative problem framing and solution finding^118^). Included sources noted that corresponding use of multipliers or “offsets for uncertainty” to strengthen harm offset requirements is debated in environmental policy.^88^,^107^,^108^ Transitional activities to address short-term impacts^28^ and evaluation over project lifecycles^90^ were suggested.
Accounting approach	Assessments of environmental changes and changes in health and well-being typically simplify complexity and may embed value loss.^107^,^126^ While multi-capital accounting may broaden understanding of impacts, quantification may exclude other forms of value.^54^
Agency problems	Net policies and practices presume particular forms of actors and can create or reinforce subjectivities.^107^ Agency considerations included differing political, institutional, and stakeholder priorities (eg, noting profit-led development^54^,^142^) and power imbalances.^71^,^92^
Development contributions	Some sources suggested a potential role for financial, non-financial, or in-kind developer contributions within health net-outcome objectives.^45^,^77^ Broader objectives targeted positive (developer) contributions across environmental or environmental, social, and economic dimensions and the possible redistributive role of financial measures, such as revenue funds, was suggested.^31^,^93^,^106^,^142^
Monitoring, evaluation, and auditing	Sources noted that goals (and progress towards them) may be locally defined through participatory processes,^53^,^130^ and emergent (unpredictable) benefits and opportunities could only be evaluated retrospectively.^91^ Suggested audit principles included transparency and independence.^100^

The most frequently mentioned inherent strengths of broader objectives (Table 6) were setting and realizing the expectation of wider-scale positive sociological benefits in furthering sustainable development. Related sources describing the specific objective of positive development emphasized the value of design-led (rather than policy or decision-dependent) approaches as a means of resolving conflicts and securing public benefits. Inherent weaknesses frequently related to issues of valuation, rights, and justice associated with balance-sheet conceptualizations, and the compensation and offsetting of negative impacts. The practical determination of the scope and nature of baselines and impacts were frequently raised assessment challenges addressed in detail by many sources.

**Table 6:** Strengths, weaknesses, opportunities, threats (SWOT) analysis of sources describing broader net-outcome objectives relevant to health (types T4-T8)

SWOT analysis	Frequent concepts (first 5, contextualized)	Potentially unique concept words* (first 5, n>2)
Strengths (54 of 90 sources)	• Emphasising the creation of wider-scale positive **impact**s (#68/23), rather than (only) reducing negative impacts	• actor (#14): *footprint, individual, multiple*
	• Resolving conflicts and increasing positive opportunities and local to systemwide socioecological (public) benefits through area-specific, **design**-led (#58/12) buildings, projects, and cities (exemplified by positive development, **PD** [#48/5]) rather than indirect policy approaches or existing decision-making processes	• capability (#8): *sustainability, HDI and EF*
		• reduction (#8): *footprint, industrialized world, binding absolute*
	• Furthering sustainable **development** (#54/22) objectives and collaboratively defining societal expectations regarding the distribution of specific financial or socioecological **benefits** (#49/25) and solutions (related to existing needs, deficits, or objections) by projects	
		• SHINE (#8): *handprint framework, scope*
		• innovation (#7): *efficient, analysis, system*
Weaknesses (58 of 90 sources)	• Agreeing the scope of relevant **impacts** (#56/24) and priority and permissibility, noting that co-benefits are not guaranteed, the effectiveness of mitigation varies and some negative impacts cannot be mitigated, trade-offs are foreseeable, and cumulative effects are challenging to assess or address	• EA (#10): *systems, implementation, frameworks*
		• right (#8): *private, private property, nature and animal*
	• Characterizing baselines and defining and demonstrating **benefits** (#41/18; ie, assuring that net-outcome objectives are met), particularly if future benefits are indirect, uncertain, subject to redistribution, or held to be strictly incommensurate	
	• Predisposing **policy** (#39/12) towards balance sheet logic, raising issues associated with commodification, valuation and **value** (#37/16) conflicts (such as omission of dimensions of value relevant to human well-being), over-simplification, and potential exacerbation of social injustice (through procedural or distributive effects)	
	• Specifying **compensation** (#39/9) and offsetting measures, raising further issues associated with assessment, design, implementation, and governance (eg, location constraints, irreplaceability, equivalence, additionality, and permanence)	
Opportunities (67 of 90 sources)	• Using and developing **impact** (#43/23) assessment frameworks and methodologies, including regulatory, sustainability, strategic, environmental, social, and life-cycle impact assessment; impact wheels; cost-benefit analysis; and multi-criteria decision analysis	• construction (#7): *sustainable, industry, processes, activities, materials*
		• planner (#7)
	• Broadening targeted **benefits** (#36/20) to incorporate climate, biodiversity, health, equity, or different **value** (#33/10) considerations; rewarding location and context-sensitive development that delivers public goods and benefits wider socioecological systems	• sector (#7): *standards, aggregate, private, third, partnerships*
		• thinking (#7): *ecosystemic, futures, joined-up, life cycle, natural capital, regenerative*
	• Incorporating net-objectives and mitigation hierarchies in spatial **planning** (#34/13) and urban and organizational **policy** (#35/20), strategies, processes, practices, and building standards; linking regional planning and strategic assessment of opportunities with site and project-level masterplans, assessments, permit applications and amendments	
Threats (58 of 90 sources)	• Emphasising negative **impacts** (#49/26), mitigation, and resource and efficiency considerations, noting status quo limitations include consideration of distributional and compensation issues, and the cumulative, inter-related and systems-level socioecological effects of development	• program (#7): *current, firms, corporate social responsibility, rehabilitation*
		• safety (#7): *health, physical, public, policy, standards, levels*
	• Limiting the scope of **development** (#39/21) through inconsistent, narrow, or overly-prescriptive regulations, **sustainability** (#32/16) standards, and institutional and organizational practices that restrict consideration of positive opportunities and socioecological **benefits** (#32/16) related to alternative development models, project designs, and locations	
		• team (#7): *design, cohesive*
		• factor (#6): *psychological, dread*
		• response (#6): *market, disaster, policy, singular*
	• Conflicting **policy** (#32/12) objectives and unresolved tensions between economic, environmental, and social objectives	

EA, environmental assessment; EF, environmental footprint; HDI, human development index; PD, positive development; SHINE, The Sustainability and Health Initiative for NetPositive Enterprise

Note: underlined frequent concepts denote a concept that was also unique between groups = unique to this data extraction target by comparison with the other group

Opportunities within the context of a SWOT analysis (Table 6) are external factors or aspects of the external environment that net-outcome policies could leverage to their advantage. Frequently described opportunities included the potential expansion of the scope of broader objectives and incorporation of new ways of thinking and different value considerations, with an emphasis on incentivizing or rewarding the future provision of public and socioecological system-wide benefits.

Spatial planning policies and processes were often referenced as potential delivery mechanisms; the related contributions of planners, sector partnerships, and sustainable construction practices were highlighted. The use and development of existing impact assessment frameworks and methods was commonly suggested as a response to associated status quo challenges. External threats described by sources describing broader objectives related to, firstly, entrenched preoccupation with negative impacts and a limited scope of thinking and assessments that was also reflected in development and (in particular) sustainability policies, standards, and practice; and, secondly, conflicts between the component (economic, environmental, and social) objectives of sustainable development itself. Notable concepts included potential issues created by market and policy responses, psychological factors, and teams involved with development projects.

Strengths frequently described in relation to distinct health objectives (Table 7) included the protection and improvement of health and, in particular, people’s well-being within the wider framework of sustainable development, and the role of such objectives as vehicles to more comprehensively address social, moral, and ethical considerations and equity. Notable associated concepts were the building of trust, social acceptability, and social licenses to operate. Conservation and biodiversity activities were frequently considered to be potential beneficiaries of linked social objectives. On the other hand, conflicts between health and other policy objectives were frequently mentioned weaknesses, as was the question of defining health and reconciling net-objectives with other (eg, equity) principles. Spatial exchanges were highlighted as a particular distributional issue. In common with broader objectives (Table 6), measurement and characterization were frequently mentioned weaknesses. Health objectives specifically focused on challenges associated with the characterization of well-being and the distribution of impacts on people (eg, individuals and groups), and issues associated with valuation, monetization, and compensation.

**Table 7:** Strengths, weaknesses, opportunities, threats (SWOT) analysis of sources describing distinct health net-outcome objectives (types T1-T3)

SWOT analysis	Frequent concepts (first 5, contextualized)	Potentially unique concept words* (first 5, n>2)
Strengths (17 of 29 sources)	• Demonstrably preventing and reducing **health** (#27/7) harms and promoting, improving, maintaining, or maximizing health, **well-being,** (#18/8) and equity benefits	• engagement (#4): *local, meaningful*
		• license (#4): *social*
		• acceptability (#3): *social*
		• trust (#3)
	• Comprehensively addressing social considerations; **people**’**s** (#27/9) relationships, values, priorities, preferences, and rights; and impacts on people	
	• Enhancing the transparency, acceptability, sustainability, equity, or efficacy of conservation and **biodiversity** (#19/8) activities	
	• Incentivizing sustainable **development** (#16/8) aligned with ethical or moral principles and the delivery of social, health, and well-being benefits	
Weaknesses (19 of 29 sources)	• Characterizing **well-being** (#34/7), noting assessment challenges associated with data collection, inclusivity, subjectivity, aggregation (reconciliation of individual and population levels), quantification, and variation over time	• AOI (#3)
		• consumer (#3)
		• diversity (#3)
	• Appropriately identifying and compensating affected **people** (#29/9), noting **impacts** (#23/10) vary spatiotemporally and contextually and issues are associated with aggregation, people’s changing values, irreplaceable impacts, and monetization	
		• exchange (#3): *spatial*
	• Defining **health** (#23/7) net-objectives, noting potential conflicts between net- and inequalities-related goals and between health and other policy objectives, such as **biodiversity** (#23/8), and uncertainties associated with causality and the prediction of (differentiated negative and positive) health outcomes	
Opportunities (25 of 29 sources)	• Linking **health** (#74/13) net-objectives with climate and health issues; environmental, energy, or spatial planning **policy** (#43/12); building design standards, codes and guidance; developer contributions; and health impact assessments and indicators	• technique (#7): *qualitative, indirect questioning, quantitative, economic valuation, economic nonmarket valuation*
		• legislation (#6): *country-specific, human rights*
		• code (#5): *design*
		• operationalisation (#5): *earlier or upstream, further*
	• Addressing existing international to local commitments to sustainable and equitable **development** (#43/11), specific development policies and processes, and specific development phases (encompassing design, construction, and operational lifetimes)	
		• threshold (#5): *social, effects*
	• Applying, developing, and integrating **impact** (#39/14) assessment frameworks, methodologies, and capacities, and addressing uncertainties associated with the scope, measurement, timescales, and acceptability of impacts through research	
	• Learning from the operationalisation of **biodiversity** (#36/10) and other environmental net-outcome objectives, and broadening their scope to include social, health or well-being considerations	
Threats (21 of 29 sources)	• Assessing **impacts** (#25/11) of **development** (#22/12), noting status quo limitations of late (post-design) phasing; scope; disaggregation of economic, environmental and social impacts and disciplines; emphasis on project-level direct and first-order effects; exclusion or deprioritization of subjective and relational elements; and insufficient resourcing or expertise	• agency (#3): *different*
		• difference (#3): *large*
		• link (#3): *explicit*
		• user (#3)
	• Incorporating **health** (#21/9) and **well-being** (#18/3) in **policy** (#23/9), noting status quo heterogeneity with respect to policy definition, inclusion or exclusion of health and well-being; variable emphasis on health costs versus benefits; institutional barriers including roles and resources; potential duplication; and issues of integration, primacy and conflicts between health and other policy objectives	

AOI, area of influence

Note: underlined frequent concepts denote a concept that was also unique between groups = unique to this data extraction target by comparison with the other group

The linking of health, specifically, to other current issues and policies was a frequently cited opportunity, as well as its incorporation within spatial planning policies and practice. Health objectives were often referenced as a means of supporting broader international to local commitments to sustainable development. The operationalization of environmental and biodiversity net-outcome policies was referenced as both an opportunity to integrate health and well-being and a model that could inform development of other net-objectives. In common with broader objectives, the use and development of existing impact assessment frameworks was commonly suggested, highlighting both qualitative and quantitative techniques. Design codes and earlier operationalization (within planning and other processes) were noted opportunities. Health objectives also emphasized threats associated with limitations in impact assessment practice. Challenges related to existing and future policy incorporation and integration of health and well-being were often described. The definition, scope, and priority of health and well-being was contextualized as a broader challenge as well as a potentially inherent weakness of health net-objectives themselves.

## Discussion

This review found that although there is increasing interest in net-objectives as explicit targets to prioritize health in spatial planning and development and narrow entrenched inequalities, examples of distinct health net-objectives were immature. Authors describing such objectives from 2019 onwards, including authors directly and indirectly associated with this review, typically presented health NG as a hypothetical requirement rather than explicating approaches. It is, therefore, timely to consider potentially transferable implementation models and their implications. This review has also characterized broader net-objectives relevant to health and well-being to inform ongoing exploration and operationalization of dedicated health net-objectives. Evidence sources presented diverse perspectives, and notable review findings are discussed hereafter mainly in the context of the wider spatial planning and health literature.

### The emergence and evolving scope of net-outcome objectives applicable to health in spatial planning

The different types of health net-objectives found by this review and their chronological development have parallels with the historical progression of public health objectives, which originate in the protection of people from acute environmental harms and sanitary issues created by urban development.^11^,^150^,^151^ During the developed world’s industrialization and urbanization, environmental protections (and frameworks such as the mitigation hierarchy featured in some net-outcome objectives) were determined and implemented, and the focus broadened and shifted towards chronic diseases and health’s social determinants. Holistic approaches to health are latterly exemplified by concepts such as planetary health in the spatial planning and public health literature.^42^,^43^,^152^,^153^

In recent decades, references to multidimensional, multidisciplinary research addressing socioecological concepts have become more frequent in the urban health literature than traditional references to the built environment.^154^ This review found many environmental and socioecological NG objectives relevant to health. These often made the case that environmental damage has accumulated despite protections, and existential environmental threats to health must once more be prioritized (and residual harms redressed) by the planning system, only now addressing both proximate and diffuse aspects.^127^,^142^,^152^,^153^

The different types of net-outcome objectives found during the conduct of the review include examples of this converging socioecological focus: social objectives incorporating environmental objectives and vice versa. It found examples of ecological objectives, such as biodiversity NG, that have recently moved to incorporate nature-linked aspects of people’s well-being. New health net-outcome objectives might continue or diverge from this path. Although the WHO constitution’s definition of health as a state of well-being^147^ was a reference common to distinct health and conservation-oriented well-being net-objectives found by the authors of this review, it is notable that anthropocentric net-objectives focused on people’s needs, and their concept of health and well-being differed in scope, focus, and emphasis from broader biocentric objectives that addressed system-level socioecological capacities for life support. These are fundamental considerations for future objective specification and are relevant to would-be net-outcome policymakers when setting the scope and boundaries of policy objectives.

This review found differing concepts of net-outcome objectives, and some evidence sources presented a distinct focus on the state and functioning of multi-scalar socioecological systems in contrast to measuring changes at the scale of individual development projects. In spatial planning, this has potential implications for the design and assessment of development projects and monitoring of health net-outcome policies. The discussion summarizes system and assessment-oriented net-objectives found by this review before commenting further on their potential incorporation within existing approaches.

### Systems-oriented net-objectives

Systems-oriented net-objectives often focused on system states and dynamic interactions rather than outcomes, sometimes presenting net-outcome objectives as frameworks for the characterization of places and participative mapping and realization of opportunities to meet needs and support thriving socioecological systems.^33^,^72^,^81^,^122^,^125^ This review found sources pushing for building designers to look beyond occupants, and for policymakers and developers to look beyond sites and neighborhoods to systems.^31^,^32^,^35^,^64^,^82^,^87^,^112^, For net-outcome policymakers seeking to address health through spatial plans and development management, this implies expansion of the temporal, spatial, and systemic scales in question, and consideration of both direct and indirect effects. Furthermore, evidence sources that emphasized the health of socioecological systems often advocated conceptual frameworks and processes that emphasized an ongoing process of identifying and realizing opportunities for health creation. The translation into policy and development processes deserves further exploration, and a related step is to consider the role of healthy design principles and post-development interactions between built and natural environments and society.

There are potential opportunities to integrate aspects of systems-oriented socioecological net-objectives with existing initiatives. Urban resilience entails appraisal of the vulnerabilities and strengths of urban systems, considering multiple hazards and cascading effects.^155^ An analogous vision for the continuous improvement of health through supportive physical and social environments is integral to established health-promotion initiatives such as the WHO Healthy Cities program.^156^-^158^ Healthy city planning also emphasizes an integrated focus on neighborhoods, the city center, and the region, and the WHO’s characteristics of a healthy city encompass economic, social, physical, and ecosystem health.^156^ Evidence sources found by this review that presented regenerative or socioecological objectives responding to the impacts of economic development typically contested lines drawn between human and ecological health.^31^,^74^,^102^,^105^,^138^ If drawing from these net-objectives in future, differences perhaps lie in the relative importance placed on the capacity of natural systems to support themselves as a precondition for the built and natural environments to support human health. There are related debates over the primacy of the economic, social, and environmental components of sustainable development.^159^,^160^ These considerations are all relevant to would-be policymakers when deciding how to define health itself within a health net-outcome objective.

### Accounting-oriented net-objectives

This review found that, overall, net-outcome objectives relevant to health were predisposed towards the measurement of outcomes (typically at the development project level), which might aid future operationalization, as this is also an established focus of urban health.^5^,^19^,^34^,^52^,^54^,^98^,^102^,^117^,^135^,^136^,^153^,^161^,^162^ A perceived strength of some objectives found by this review was an explicit requirement for neutrality or improvement—which arguably requires measurement—but accounting mechanisms entailed the prediction and balancing of gains and losses within frameworks predisposed towards the facilitation of exchanges and were contested.^75^,^107^,^119^,^120^,^149^ To illustrate, one source advocated cost-benefit analysis in assessments of community NG; however, in project appraisal, the applicability of economic values to social and environmental capital is contested and considered by some a barrier to stakeholder debate.^5^,^163^ Uncertainties and issues related to valuation, assessment, and exchange (ie, value incommensurability) were challenges commonly reported in relation to established ecological and environmental net-outcome objectives, but these challenges also applied to consideration of people’s well-being specifically.

If prospective policymakers opt for similar accounting frameworks for future health net-outcome objectives, these findings indicate that defining net outcomes through measurement will pose practical challenges and require value judgments in terms of what is, or is not, addressed through measurement (and related issues as summarized in Table 5). Some of these issues are already discussed within the spatial planning and health literature, which documents the consideration of health needs and broader concepts of health that incorporate qualitative measures of well-being alongside quantifiable health outcomes.^38^,^164^,^165^ The potential facilitation of exchange by net-outcome accounting mechanisms is a double-edged sword in that inequalities can be exacerbated or narrowed.^24^,^30^,^66^,^104^ Therefore, distributive considerations, which encompass the spatiotemporal distribution of gains and losses or their distribution between individuals or population groups, are particularly important to address within the design of accounting-oriented health net-outcome policies.

Evidence sources found by the review and the wider literature provide further guidance on this matter, and indicate that assessments should consider groups and environmental, social, and economic aspects.^43^,^104^,^161^,^166^ Addressing health inequity requires societal, regional, and local foci. Needs vary greatly between disadvantaged population groups,^166^ and it is important to consider socioeconomic status and different aspects of deprivation.^153^ To map opportunities and inequalities, spatially disaggregated indicators are required at neighborhood or lower scales.^165^,^167^ However, communities can also be defined by common interests in planning matters, and the socioecological net-outcome objectives previously discussed also raise the question of the distribution of environmental and ecological impacts.

### Health impact assessment and monitoring of health net-outcome objectives in spatial planning

Sources describing net-objectives and the existing spatial planning health impact assessment (HIA) literature both stress the importance of healthy design and upstream consideration of policies and projects’ opportunities to create health.^43^,^46^,^94^,^168^ This positions HIA as a potential delivery mechanism for health net-outcome objectives whether they are oriented towards healthy design, assessment of net outcomes, or both. While HIAs can be particularly useful when exploring multiple future options,^161^ they have sometimes been criticized for comparing preexisting options rather than finding optimal solutions.^169^ Furthermore, if decision-makers are ultimately the arbiters of net outcomes (ie, the net outcome is not predefined), the wider spatial planning and HIA literature indicate that the impact of health appraisals and recommendations on decision-making are not always evidenced,^161^,^168^ which is a relevant consideration for the practical definition of net outcomes.

Conservation net-objectives found by this review discuss issues related to the predication of net-outcome objectives on single assessments linked to planning applications. Once a local spatial planning policy or development has been implemented, benefits thereafter depend on curation, maintenance, and continued provision of services and an ongoing process of identifying and realizing opportunities.^102^,^170^ This is an important consideration for net-outcome policymakers. Potential responses to uncertainties offered by the wider literature include the principles of long-term, multi-sector, multicomponent investment^166^ and the use of development-level health management plans and monitoring.^161^,^170^ While repeated, rigorous evaluation of health outcomes is desirable,^166^,^168^ monitoring is not necessarily well addressed by existing healthy planning frameworks.^164^,^171^ Future evaluation of the realization of net health outcomes could likely encounter existing challenges faced by public health in spatial planning related to indicator data availability,^155^,^167^ issues of timeliness or quality,^153^,^172^ and inconsistent requirements for the monitoring of local policies and plan effects^167^,^173^ or post-construction monitoring of health metrics by developers.^168^,^170^

To address the multiple challenges facing sustainable healthier cities, multiple and boundary-spanning indicators are required.^162^ Given the predisposition towards measurement of many net-outcome objectives found by this review, it is helpful to consider the current role of indicators in addressing the complexity of urban health, which is not well defined,^174^ as well as their future use by health net-outcome policies. Urban health indicators (UHIs) or composite indices can simplify or mask complexity,^165^ and this was also recognized by evidence sources describing net-outcome metrics. Although a significant diversity of UHIs exist, their use in policy and decision-making remains limited,^165^ and existing indicator frameworks differ in terms of scope and the extent to which baselines and urban planning interventions are characterized.^155^ The prescriptive, accounting-oriented approach featured in many net-outcome objectives found by this review could, therefore, require a significant shift. The standardization of UHIs has been suggested while allowing for local prioritization and adaptation,^174^ reflecting the fact that values placed on different aspects of health vary between cultures and individuals,^151^ and approaches to health assessment and valuation can create value conflicts and inequity. Health net-outcome objectives for spatial planning will need to engage further with these overlapping issues, which challenge the prescriptive approach described by some evidence sources and indicate it may be important to adapt the way in which success is defined or how impacts are measured at the development and policy levels according to local context.

### Operationalization of health net-outcome objectives in spatial planning

This review found differing values and focuses of objectives and concepts of net outcomes. Spatial planning systems express social and cultural values,^175^ so their compatibility is a prime question. Principles that are potentially shared with net-outcome objectives include equity, social justice, sustainability, community participation and empowerment, intersectoral cooperation, and accountability,^158^ among others. Public health and urban planning share the longstanding dilemma of determining the extent individual freedoms can legitimately be restricted in favor of the public good,^151^ and this review indicates that this is a specific consideration for net-outcome objectives when considering both the maximization of benefits or nature and distribution of benefits and losses. Further more, policymakers’ standards and values are dynamic, and governmental determination of the scope of protection and public welfare (and health’s reconciliation with other policy objectives) is likely to raise issues of power, justice, and equity.^176^

This review positions health net-objectives for spatial planning as emergent and yet to adopt consistent positions in response to issues identified in the environmental literature (as summarized in Table 5). Uncertainties and issues associated with assessment and measurement appear inherent to net-outcome objectives, but the wider literature indicates that such challenges were already established, especially if attempting to draw clear causal links between built and natural environmental exposures and health-related impacts and outcomes.^169^,^174^,^177^ The importance of participatory approaches to net-outcome objectives’ implementation is, similarly, already emphasized in the health and spatial planning literature.^152^,^156^,^166^,^174^ In the context of assessments, participatory quantitative HIA methods, in particular, could be important to the future development of health net-objectives but require further development and their use is not widespread.^161^,^162^

The review identified positive opportunities related to the targeting of inequalities and development and use of existing assessment approaches and indicators, and the effects of some environmental changes on health outcomes can be quantified so that harms (typically) or benefits are transparent.^48^,^161^ Values associated with healthy planning principles appeared broadly compatible with net-objectives found by this review in that examples can be found of their support for both rationalist and constructivist approaches,^163^,^174^ as can the inclusion of qualitative aspects of health and well-being within assessments, while upholding principles of the mitigation hierarchy (eg, damage avoidance and minimization).^164^,^177^ Sources included in this review noted that the earlier steps of the mitigation hierarchy could be undermined if net-outcome approaches were applied without safeguards (eg, to prevent the preferential realization and offsetting of harms),^75^,^107^ which policymakers will need to consider.

Sources found by this review interpreted net-objectives’ specification and definition differently, and some advocated transitions in mindsets and approaches that emphasize the creation of positive opportunities,^33^,^72^,^81^,^122^,^125^ with an emphasis on the public good and ecological base.^31^ Some authors have recently argued for a focus more on the functions of places rather than their features or state^178^: what they do for whom, rather than what they are. Future research might perhaps explore the integration of socioecological and healthy planning frameworks to characterize interactions, combined flows of benefits, and effects on health and well-being of the natural *and* built environments, and incorporate consideration of their respective capacities to meet needs and support health in perpetuity.

### Limitations of the review

The review faced conceptual and practical challenges arising from its broad scope and number of search results. The aim of scoping as-yet-unknown conceptual boundaries can conflict with conceptual and contextual limits specified by inclusion criteria. In this case, we attempted to reconcile sensitivity with specificity by including borderline eligible sources during title and abstract screening and excluding uncertain interpretations at the full-text review stage. Examples include decisions to exclude many biodiversity and ecology-oriented sources as not unambiguously transferable to health contexts and only include sources addressing regenerative concepts if they referenced net terms and clearly explicated the evaluative element arguably implied by the term and often used in other contexts. The latter example is notable because the review found that a dominant quantitative paradigm was sometimes, if not often, criticized by authors describing regenerative concepts. In mitigation of this potential bias, sources that directly addressed evaluation and alternative definitions of net outcomes were included within this review,^(eg,^^102^^)^ as were sources that presented, inter alia, overarching ethical, equity, or valuation considerations. In contrast, the review could equally be criticized for excluding sources describing economic evaluations and cost-benefit analysis unless they specifically addressed net-outcome policy considerations or the context of spatial planning.

Another consideration is that the review included evidence sources with common authors, which may contribute to the consistency of findings across evidence sources. Furthermore, some evidence sources describing (type T1) stand-alone health objectives were associated with the review authors themselves. While these met eligibility criteria, their inclusion introduced risk of bias because independent screening or extraction of these sources was not possible due to the context of the review team members’ PhD researcher or supervisory interests. As comparatively few directly authored evidence sources (4) were included, they are considered unlikely to have resulted in a substantial impact on overall findings given the total number of included evidence sources (119).

The imposed limits and review decisions discussed potentially increased the subjectivity of source selection and data extraction. Furthermore, the diverse content, number, and types of evidence sources (which included gray literature reports, theses, and books) restricted the number of full-text sources subject to second review at the screening and data extraction stages; however, as good levels of agreement between the reviewers were seen, the potential impact on the findings is likely to be minimal.

Modifications to the original data extraction instrument aimed to clarify and extend the information extracted and presented by the review. The authors ultimately aimed to provide a comprehensive yet accessible review report and rich dataset (Supplemental File 2: http://links.lww.com/SRX/A147) to other researchers; however, the creation of summary categorical variables, use of square brackets in text excerpts to define acronyms on first use and provide minor contextual clarifications to readers, and use of concept mapping in analysis also introduce some subjectivity and simplifications.

Finally, extracted data are extensive and invite further qualitative data analysis that is beyond the scope of a conventional scoping review. Similarly, assessment of the quality of sources was out of the scope of this review.

## Conclusions

There is increasing interest in net-objectives as explicit health targets to prioritize health in spatial planning and development and narrow entrenched inequalities, but this review found few examples of their maturation and operationalization in policy or practice in this domain. This review has characterized emergent examples and a broader suite of established social and environmental net-objectives relevant to health, from which potential implementation models and lessons can be drawn by future policymakers seeking to emphasize health within potentially competing economic, environmental, and social objectives and housebuilding targets. Examples of net-outcome objectives relevant to health predominantly originated from Western economies and conservation, design, and development perspectives with a trend of increasing publications either emphasizing socioecological net outcomes or more directly addressing health and well-being.

Rationales for net-outcome objectives included prioritization of specified objectives and redress of accumulated environmental harms associated with unsustainable development. While the WHO constitution’s definition of health as a state of well-being was a common reference, sources also described biocentric objectives that applied to system-level socioecological capacities for life support. These included the use of conceptual frameworks and processes that emphasized ongoing identification and realization of opportunities for health creation rather than the application of quantitative accounting frameworks, which were a predominant feature of net-outcome objectives characterizing the effects of development projects. While there are opportunities to integrate both perspectives within existing spatial planning practice, future policymakers face value judgments associated with the focus, scope, and specification of health net-outcome objectives that deserve further exploration.

Uncertainties and issues related to valuation, assessment, and commensurability were challenges common to established environmental net-outcome objectives and consideration of well-being specifically; the development and use of existing methodologies (such as HIA) and indicators was seen as a related opportunity. Implementation principles included a general emphasis on participatory approaches and assessment, and this review found that it is important for policymakers to consider the distributional stances of net-outcome objectives, which can exacerbate or narrow health inequalities. Future evaluation of the impacts of established systems- and accounting-oriented net-outcome objectives could inform their future development.

### Implications for research

Knowledge gaps identified as potential focuses of future research are as follows:

- the compatibility of different net-outcome approaches and implementation principles with existing spatial planning systems, plan-making and development processes, and social and cultural values
- the implications of health net-outcome objectives and approaches for impact assessment theory and practice, particularly environmental and health impact assessment
- the evaluated effects on health of operationalized net-outcome objectives and approaches (at both policy and project level)
- the potential role of health net-outcome objectives and approaches in addressing mental health
- considerations posed for health net-outcome objectives and approaches in developing (as opposed to developed) economies
- the integration of socioecological and healthy planning frameworks to characterize interactions between the natural *and* built environments and health and well-being.

Further state-of-the-art or meta-narrative reviews could extend this scoping review’s preliminary characterization of the development of health net-objectives or explore differences in the interpretation of net-outcome objectives across the various disciplines and perspectives represented herein. The comprehensive dataset created by this scoping review (Supplemental File 2: http://links.lww.com/SRX/A147) invites further thematic analysis, differentiation, and elaboration of the underlying values and implementation and assessment principles of net-outcome objectives. This review’s preliminary characterization of existing net-outcome objectives and approaches provides a range of options for future country-specific development, exploration, and evaluation of health net-outcome objectives for spatial planning policy and practice.

## Acknowledgments

Patricia Lacey, UK Health Security Agency (UKHSA) Knowledge and Evidence Specialist, and Alison Ashmore, University of Nottingham Senior Research Librarian, for their assistance with the search strategy.

## Funding

JSE is the recipient of a PhD studentship funded by the UKHSA. Any views expressed in this review are those of the authors and not necessarily those of the UKHSA or the Department of Health and Social Care.

## Author contributions

JSE and JLB conceived the idea. JSE developed the research protocol and methods, conducted searches, and drafted and edited the manuscript. EW, TL, and AH helped to refine and develop the research protocol and methods. JSE and AH piloted inclusion and exclusion criteria in Covidence and screened sources. JSE carried out data extraction and large language model review exercises. AH checked extracted data and results. JLB provided methodological guidance, critical review, and editorial direction throughout the process. All authors approved the final manuscript submitted.

## Availability of data

See Supplemental File 2 (http://links.lww.com/SRX/A147) for the full data extraction dataset.

As 542 reports were assessed for eligibility, reasons for exclusion are summarized in the manuscript. Further details (ie, individual information source exclusions) are available on request.

## Appendix I: Search strategy

*Summary*

Nineteen databases were searched: databases, search dates, and total records retrieved are summarized below and then detailed per database.

**Table:**

Database	Platform	Nominal content	Search dates	Records retrieved (total)
Scopus	N/A	Academic	17/08/23; 12/07/24	4855
BASE (Bielefeld Academic Search Engine)	N/A	Gray	17–18/08/23; 17/07/24	4694
Embase	Ovid	Academic	17/08/23; 12/07/24	2430
MEDLINE	Ovid	Academic	17/08/23; 12/07/24	1728
TRIP Pro	N/A	Gray	17/08/23; 12/07/24	1586
IBSS (International Bibliography of the Social Sciences)	ProQuest	Academic	17/08/23; 18/07/24	720
Dissertations and Theses A&I	ProQuest	Gray	17/08/23; 12/07/24	571
APA PsycINFO	Ovid	Academic	17/08/23; 12/07/24	530
PAIS Index	ProQuest	Academic	17/08/23; 12/07/24	319
Social Science Database	ProQuest	Academic	17/08/23; 12/07/24	289
Sociological Abstracts	ProQuest	Academic	17/08/23; 12/07/24	218
ASSIA (Applied Social Sciences Index and Abstracts)	ProQuest	Academic	17/08/23; 12/07/24	155
Political Science Database	ProQuest	Academic	17/08/23; 12/07/24	133
Worldwide Political Science Abstracts	ProQuest	Academic	17/08/23; 12/07/24	120
Policy File	ProQuest	Academic	17/08/23; 12/07/24	95
Sociology Database	ProQuest	Academic	17/08/23; 12/07/24	80
Social Services Abstracts	ProQuest	Academic	17/08/23; 12/07/24	71
Google	N/A	Gray	30–31/08/23; 17/07/24	51
HMIC (Health Management Information Consortium)	Ovid	Academic	17/08/23; 12/07/24	39

**Table:**

Scopus		
**Search conducted: August 17, 2023**		
**Search**	**Query**	**Records retrieved**
#1	TITLE-ABS-KEY (“net outcome*” or “net gain*” or “net positive outcome*” or “net positive impact*” or “no net loss*” or “mitigation hierarch*” OR “regenerative design*” OR “regenerative development*” OR “regenerative sustainability”)	6003
#2	KEY (“net positive*” OR “net benefit*” OR “positive development*”)	1028
#3	1 or 2	7006
#4	KEY (theor* or concept* or principle* or policy or policies or plan or plans or planning or strategy or strategies or objective* or aim* or goal* or target* or requirement* or framework* or model* or approach* or practice*)	16,192,577
#5	TITLE-ABS-KEY (“health risk assessment*” OR “impact assessment*” OR “environmental assessment*” OR “assessment tool*” OR “assessment method*”)	285,311
#6	TITLE-ABS-KEY (hia OR eia OR “sustainability appraisal*”)	22,980
#7	TITLE-ABS-KEY (mitigation* OR “hierarchical system*” OR “response hierarch*” OR compensation* OR offset* OR “off-set*” OR tradeoff* OR “trade-off*” OR reparation* OR restitution*)	1,126,214
#8	4 or 5 or 6 or 7	17,161,336
#9	TITLE-ABS-KEY ((“net outcome*” OR “net gain*” OR “net benefit*” OR “net improve*” OR “net increase*” OR “net positive*” OR “positive outcome*” OR “net positive outcome*” OR “positive impact*” OR “net positive impact*” OR “positive development*” OR “regenerative design*” OR “regenerative development*” OR “regenerative sustainability” OR “no net loss*” OR “mitigation hierarch*”) PRE/0 (theor* or concept* or principle* or polic* or plan* or strateg* or objective* or aim* or goal* or target* or requirement* or framework* or model* or assessment* or appraisal* or evaluation* or approach* or practice* or tool*))	426
#10	3 and 8	2380
#11	10 or 9	2651
#12	TITLE-ABS-KEY (health* OR wellbeing OR “well-being” OR welfare)	7,511,144
#13	KEY (social* OR societal* OR society OR societies OR sociodemographic* OR socioeconomic* OR sociological*)	2,332,935
#14	KEY (human* OR population* OR stakeholder* OR community OR communities)	25,227,270
#15	12 or 13 or 14	28,401,228
#16	3 and 15	2040
#17	TITLE-ABS-KEY ((health* OR wellbeing OR “well-being” OR welfare OR soci* OR commun*) W/2 net W/2 (gain* OR benefit* OR improve* OR increase* OR positive*))	1645
#18	8 and 17	773
#19	TITLE-ABS-KEY (“health net gain*” OR “net health gain*” OR “wellbeing net gain*” OR “net wellbeing gain*” OR “well-being net gain*” OR “net well-being gain*”)	27
#20	11 or 16 or 18 or 19	4549

Note: as a single query (which represents the consolidated August 17, 2023 query #20 with an additional date delimiter) was used in the updated July 12, 2024 search; no breakdown of component queries is provided.

**Table:**

Scopus		
Search conducted: July 12, 2024		
Search	Query	Records retrieved
#1	((((TITLE-ABS-KEY(“net outcome*” or “net gain*” or “net positive outcome*” or “net positive impact*” or “no net loss*” or “mitigation hierarch*” OR “regenerative design*” OR “regenerative development*” OR “regenerative sustainability”)) OR (KEY (“net positive*” OR “net benefit*” OR “positive development*”))) AND ((KEY (theor* or concept* or principle* or policy or policies or plan or plans or planning or strategy or strategies or objective* or aim* or goal* or target* or requirement* or framework* or model* or approach* or practice*)) OR (TITLE-ABS-KEY (“health risk assessment*” OR “impact assessment*” OR “environmental assessment*” OR “assessment tool*” OR “assessment method*”)) OR (TITLE-ABS-KEY (hia OR eia OR “sustainability appraisal*”)) OR (TITLE-ABS-KEY (mitigation* OR “hierarchical system*” OR “response hierarch*” OR compensation* OR offset* OR “off-set*” OR tradeoff* OR “trade-off*” OR reparation* OR restitution*)))) OR (TITLE-ABS-KEY ((“net outcome*” OR “net gain*” OR “net benefit*” OR “net improve*” OR “net increase*” OR “net positive*” OR “positive outcome*” OR “net positive outcome*” OR “positive impact*” OR “net positive impact*” OR “positive development*” OR “regenerative design*” OR “regenerative development*” OR “regenerative sustainability” OR “no net loss*” OR “mitigation hierarch*”) PRE/0 (theor* or concept* or principle* or polic* or plan* or strateg* or objective* or aim* or goal* or target* or requirement* or framework* or model* or assessment* or appraisal* or evaluation* or approach* or practice* or tool*)))) OR (((TITLE-ABS-KEY (health* OR wellbeing OR “well-being” OR welfare)) OR (KEY (social* OR societal* OR society OR societies OR sociodemographic* OR socioeconomic* OR sociological*)) OR (KEY (human* OR population* OR stakeholder* OR community OR communities))) AND ((TITLE-ABS-KEY(“net outcome*” or “net gain*” or “net positive outcome*” or “net positive impact*” or “no net loss*” or “mitigation hierarch*” OR “regenerative design*” OR “regenerative development*” OR “regenerative sustainability”)) OR (KEY (“net positive*” OR “net benefit*” OR “positive development*”)))) OR ((TITLE-ABS-KEY ((health* OR wellbeing OR “well-being” OR welfare OR soci* OR commun*) W/2 net W/2 (gain* OR benefit* OR improve* OR increase* OR positive*))) AND ((KEY (theor* or concept* or principle* or policy or policies or plan or plans or planning or strategy or strategies or objective* or aim* or goal* or target* or requirement* or framework* or model* or approach* or practice*)) OR (TITLE-ABS-KEY (“health risk assessment*” OR “impact assessment*” OR “environmental assessment*” OR “assessment tool*” OR “assessment method*”)) OR (TITLE-ABS-KEY (hia OR eia OR “sustainability appraisal*”)) OR (TITLE-ABS-KEY (mitigation* OR “hierarchical system*” OR “response hierarch*” OR compensation* OR offset* OR “off-set*” OR tradeoff* OR “trade-off*” OR reparation* OR restitution*)))) OR (TITLE-ABS-KEY (“health net gain*” OR “net health gain*” OR “wellbeing net gain*” OR “net wellbeing gain*” OR “well-being net gain*” OR “net well-being gain*”)) AND ORIG-LOAD-DATE AFT 20230817	306

**Table:**

BASE			
**Search**	**Query**	**Records retrieved (17-18/08/23)**	**Records retrieved (17/07/24)**
#1	(“net outcome” “net gain” “net positive outcome” “net positive impact” “no net loss” “mitigation hierarchy” “regenerative design” “regenerative development” “regenerative sustainability”)	7613	9942
#2	subj:(theor* concept* principle* policy policies plan plans planning strategy strategies objective* aim* goal* target* requirement* framework* model* approach* practice*)	14,841,710	11,276,357
#3	subj:(“health risk assessment” “impact assessment” “environmental assessment” “assessment tool” “assessment method”)	51,463	64,818
#4	subj:(hia eia “sustainability appraisal”)	2329	2936
#5	subj:(mitigation* “hierarchical system” “response hierarchy” compensation* offset* “off-set” tradeoff* “trade-off” reparation* restitution*)	157,662	189,692
#6	subj:(theor* concept* principle* policy policies plan plans planning strategy strategies objective* aim* goal* target* requirement* framework* model* approach* practice* “health risk assessment” “impact assessment” “environmental assessment” “assessment tool” “assessment method” hia eia “sustainability appraisal” mitigation* “hierarchical system” “response hierarchy” compensation* offset* “off-set” tradeoff* “trade-off” reparation* restitution*)	14,998,555	11,469,712
#7	(“net gain” “net positive outcome” “net positive impact” “regenerative design” “regenerative development” “regenerative sustainability”) subj:(theor* concept* principle* policy policies plan plans planning strategy strategies objective* aim* goal* target* requirement* framework* model* approach* practice* “health risk assessment” “impact assessment” “environmental assessment” “assessment tool” “assessment method” hia eia “sustainability appraisal” mitigation* “hierarchical system” “response hierarchy” compensation* offset* “off-set” tradeoff* “trade-off” reparation* restitution*)	651	720
	#7 year:[2023 TO 2024]	N/A	96
#8	(“net outcome” “no net loss” “mitigation hierarchy”) subj:(theor* concept* principle* policy policies plan plans planning strategy strategies objective* aim* goal* target* requirement* framework* model* approach* practice* “health risk assessment” “impact assessment” “environmental assessment” “assessment tool” “assessment method” hia eia “sustainability appraisal” mitigation* “hierarchical system” “response hierarchy” compensation* offset* “off-set” tradeoff* “trade-off” reparation* restitution*)	846	1062
	#8 year:[2023 TO 2024]	N/A	178
#9	(health* wellbeing “well-being” welfare)	21,173,122	24,439,296
#10	subj:(health* wellbeing “well-being” welfare)	8,097,773	5,742,513
#11	subj:(social* societal* society societies sociodemographic* socioeconomic* sociological*)	9,428,700	9,780,586
#12	subj:(human* population* stakeholder* community communities)	8,795,125	9,162,466
#13	subj:(health* wellbeing “well-being” welfare social* societal* society societies sociodemographic* socioeconomic* sociological* human* population* stakeholder* community communities)	20,782,034	18,672,235
#14	(“net gain” “net positive outcome” “net positive impact” “regenerative design” “regenerative development” “regenerative sustainability”) subj:(health* wellbeing “well-being” welfare social* societal* society societies sociodemographic* socioeconomic* sociological* human* population* stakeholder* community communities)	667	751
	#14 year:[2023 TO 2024]	N/A	106
#15	(“net outcome” “no net loss” “mitigation hierarchy”) subj:(health* wellbeing “well-being” welfare social* societal* society societies sociodemographic* socioeconomic* sociological* human* population* stakeholder* community communities)	457	560
	#15 year:[2023 TO 2024]	N/A	53
#16	(health* wellbeing “well-being” welfare) subj:(“net outcome” “net gain” “net positive outcome” “net positive impact” “no net loss” “mitigation hierarchy” “regenerative design” “regenerative development” “regenerative sustainability” “net positive” “net benefit” “positive development”)	313	452
	#16 year:[2023 TO 2024]	N/A	113
#17	(“health net gain” “net health gain” “wellbeing net gain” “net wellbeing gain” “well-being net gain” “net well-being gain”)	24	39
	#17 year:[2023 TO 2024]	N/A	10
#18	(“net gain theory” “net gain concept” “net gain principle” “net gain policy” “net gain policies” “net gain strategy” “net gain objective” “net gain aim” “net gain goal” “net gain target” “net gain requirement” “net gain framework” “net gain model” “net gain assessment” “net gain appraisal” “net gain evaluation” “net gain approach” “net gain practice” “net gain tool” “net gain plan” “net gain planning”)	26	40
	#18 year:[2023 TO 2024]	N/A	12
#19	(“net outcome theory” “net outcome concept” “net outcome principle” “net outcome policy” “net outcome policies” “net outcome strategy” “net outcome objective” “net outcome aim” “net outcome goal” “net outcome target” “net outcome requirement” “net outcome framework” “net outcome model” “net outcome assessment” “net outcome appraisal” “net outcome evaluation” “net outcome approach” “net outcome practice” “net outcome tool” “net outcome plan” “net outcome planning”)	9	14
	#19 year:[2023 TO 2024]	N/A	1
#20	(“net benefit theory” “net benefit concept” “net benefit principle” “net benefit policy” “net benefit policies” “net benefit strategy” “net benefit objective” “net benefit aim” “net benefit goal” “net benefit target” “net benefit requirement” “net benefit framework” “net benefit model” “net benefit assessment” “net benefit appraisal” “net benefit evaluation” “net benefit approach” “net benefit practice” “net benefit tool” “net benefit plan” “net benefit planning”)	188	240
	#20 year:[2023 TO 2024]	N/A	20
#21	(“net improvement theory” “net improvement concept” “net improvement principle” “net improvement policy” “net improvement policies” “net improvement strategy” “net improvement objective” “net improvement aim” “net improvement goal” “net improvement target” “net improvement requirement” “net improvement framework” “net improvement model” “net improvement assessment” “net improvement appraisal” “net improvement evaluation” “net improvement approach” “net improvement practice” “net improvement tool” “net improvement plan” “net improvement planning”)	0	0
#22	(“net increase theory” “net increase concept” “net increase principle” “net increase policy” “net increase policies” “net increase strategy” “net increase objective” “net increase aim” “net increase goal” “net increase target” “net increase requirement” “net increase framework” “net increase model” “net increase assessment” “net increase appraisal” “net increase evaluation” “net increase approach” “net increase practice” “net increase tool” “net increase plan” “net increase planning”)	2	3
	#22 year:[2023 TO 2024]	N/A	0
#23	(“net positive theory” “net positive concept” “net positive principle” “net positive policy” “net positive policies” “net positive strategy” “net positive objective” “net positive aim” “net positive goal” “net positive target” “net positive requirement” “net positive framework” “net positive model” “net positive assessment” “net positive appraisal” “net positive evaluation” “net positive approach” “net positive practice” “net positive tool” “net positive plan” “net positive planning”)	4	5
	#23 year:[2023 TO 2024]	N/A	2
#24	(“positive outcome theory” “positive outcome concept” “positive outcome principle” “positive outcome policy” “positive outcome policies” “positive outcome strategy” “positive outcome objective” “positive outcome aim” “positive outcome goal” “positive outcome target” “positive outcome requirement” “positive outcome framework” “positive outcome model” “positive outcome assessment” “positive outcome appraisal” “positive outcome evaluation” “positive outcome approach” “positive outcome practice” “positive outcome tool” “positive outcome plan” “positive outcome planning”)	24	30
	#24 year:[2023 TO 2024]	N/A	3
#25	(“net positive outcome theory” “net positive outcome concept” “net positive outcome principle” “net positive outcome policy” “net positive outcome policies” “net positive outcome strategy” “net positive outcome objective” “net positive outcome aim” “net positive outcome goal” “net positive outcome target” “net positive outcome requirement” “net positive outcome framework” “net positive outcome model” “net positive outcome assessment” “net positive outcome appraisal” “net positive outcome evaluation” “net positive outcome approach” “net positive outcome practice” “net positive outcome tool” “net positive outcome plan” “net positive outcome planning”)	0	0
#26	(“positive impact theory” “positive impact concept” “positive impact principle” “positive impact policy” “positive impact policies” “positive impact strategy” “positive impact objective” “positive impact aim” “positive impact goal” “positive impact target” “positive impact requirement” “positive impact framework” “positive impact model” “positive impact assessment” “positive impact appraisal” “positive impact evaluation” “positive impact approach” “positive impact practice” “positive impact tool” “positive impact plan” “positive impact planning”)	25	34
	#26 year:[2023 TO 2024]	N/A	7
#27	(“net positive impact theory” “net positive impact concept” “net positive impact principle” “net positive impact policy” “net positive impact policies” “net positive impact strategy” “net positive impact objective” “net positive impact aim” “net positive impact goal” “net positive impact target” “net positive impact requirement” “net positive impact framework” “net positive impact model” “net positive impact assessment” “net positive impact appraisal” “net positive impact evaluation” “net positive impact approach” “net positive impact practice” “net positive impact tool” “net positive impact plan” “net positive impact planning”)	1	2
	#27 year:[2023 TO 2024]	N/A	0
#28	(“positive development theory” “positive development concept” “positive development principle” “positive development policy” “positive development policies” “positive development strategy” “positive development objective” “positive development aim” “positive development goal” “positive development target” “positive development requirement” “positive development framework” “positive development model” “positive development assessment” “positive development appraisal” “positive development evaluation” “positive development approach” “positive development practice” “positive development tool” “positive development plan” “positive development planning”)	35	43
	#28 year:[2023 TO 2024]	N/A	5
#29	(“regenerative design theory” “regenerative design concept” “regenerative design principle” “regenerative design policy” “regenerative design policies” “regenerative design strategy” “regenerative design objective” “regenerative design aim” “regenerative design goal” “regenerative design target” “regenerative design requirement” “regenerative design framework” “regenerative design model” “regenerative design assessment” “regenerative design appraisal” “regenerative design evaluation” “regenerative design approach” “regenerative design practice” “regenerative design tool” “regenerative design plan” “regenerative design planning”)	28	38
	#29 year:[2023 TO 2024]	N/A	16
#30	(“regenerative development theory” “regenerative development concept” “regenerative development principle” “regenerative development policy” “regenerative development policies” “regenerative development strategy” “regenerative development objective” “regenerative development aim” “regenerative development goal” “regenerative development target” “regenerative development requirement” “regenerative development framework” “regenerative development model” “regenerative development assessment” “regenerative development appraisal” “regenerative development evaluation” “regenerative development approach” “regenerative development practice” “regenerative development tool” “regenerative development plan” “regenerative development planning”)	8	12
	#30 year:[2023 TO 2024]	N/A	3
#31	(“regenerative sustainability theory” “regenerative sustainability concept” “regenerative sustainability principle” “regenerative sustainability policy” “regenerative sustainability policies” “regenerative sustainability strategy” “regenerative sustainability objective” “regenerative sustainability aim” “regenerative sustainability goal” “regenerative sustainability target” “regenerative sustainability requirement” “regenerative sustainability framework” “regenerative sustainability model” “regenerative sustainability assessment” “regenerative sustainability appraisal” “regenerative sustainability evaluation” “regenerative sustainability approach” “regenerative sustainability practice” “regenerative sustainability tool” “regenerative sustainability plan” “regenerative sustainability planning”)	12	15
	#31 year:[2023 TO 2024]	N/A	2
#32	(“no net loss theory” “no net loss concept” “no net loss principle” “no net loss policy” “no net loss policies” “no net loss strategy” “no net loss objective” “no net loss aim” “no net loss goal” “no net loss target” “no net loss requirement” “no net loss framework” “no net loss model” “no net loss assessment” “no net loss appraisal” “no net loss evaluation” “no net loss approach” “no net loss practice” “no net loss tool” “no net loss plan” “no net loss planning”)	185	226
	#32 year:[2023 TO 2024]	N/A	15
#33	(“mitigation hierarchy theory” “mitigation hierarchy concept” “mitigation hierarchy principle” “mitigation hierarchy policy” “mitigation hierarchy policies” “mitigation hierarchy strategy” “mitigation hierarchy objective” “mitigation hierarchy aim” “mitigation hierarchy goal” “mitigation hierarchy target” “mitigation hierarchy requirement” “mitigation hierarchy framework” “mitigation hierarchy model” “mitigation hierarchy assessment” “mitigation hierarchy appraisal” “mitigation hierarchy evaluation” “mitigation hierarchy approach” “mitigation hierarchy practice” “mitigation hierarchy tool” “mitigation hierarchy plan” “mitigation hierarchy planning”)	34	39
	#33 year:[2023 TO 2024]	N/A	6
#34	(“net gain theories” “net gain concepts” “net gain principles” “net gain strategies” “net gain objectives” “net gain aims” “net gain goals” “net gain targets” “net gain requirements” “net gain frameworks” “net gain models” “net gain assessments” “net gain appraisals” “net gain evaluations” “net gain approaches” “net gain practices” “net gain tools” “net gain plans”)	10	12
	#34 year:[2023 TO 2024]	N/A	7
#35	(“net outcome theories” “net outcome concepts” “net outcome principles” “net outcome strategies” “net outcome objectives” “net outcome aims” “net outcome goals” “net outcome targets” “net outcome requirements” “net outcome frameworks” “net outcome models” “net outcome assessments” “net outcome appraisals” “net outcome evaluations” “net outcome approaches” “net outcome practices” “net outcome tools” “net outcome plans”)	5	12
	#35 year:[2023 TO 2024]	N/A	4
#36	(“net benefit theories” “net benefit concepts” “net benefit principles” “net benefit strategies” “net benefit objectives” “net benefit aims” “net benefit goals” “net benefit targets” “net benefit requirements” “net benefit frameworks” “net benefit models” “net benefit assessments” “net benefit appraisals” “net benefit evaluations” “net benefit approaches” “net benefit practices” “net benefit tools” “net benefit plans”)	21	24
	#36 year:[2023 TO 2024]	N/A	2
#37	(“net improvement theories” “net improvement concepts” “net improvement principles” “net improvement strategies” “net improvement objectives” “net improvement aims” “net improvement goals” “net improvement targets” “net improvement requirements” “net improvement frameworks” “net improvement models” “net improvement assessments” “net improvement appraisals” “net improvement evaluations” “net improvement approaches” “net improvement practices” “net improvement tools” “net improvement plans”)	0	0
#38	(“net increase theories” “net increase concepts” “net increase principles” “net increase strategies” “net increase objectives” “net increase aims” “net increase goals” “net increase targets” “net increase requirements” “net increase frameworks” “net increase models” “net increase assessments” “net increase appraisals” “net increase evaluations” “net increase approaches” “net increase practices” “net increase tools” “net increase plans”)	0	0
#39	(“net positive theories” “net positive concepts” “net positive principles” “net positive strategies” “net positive objectives” “net positive aims” “net positive goals” “net positive targets” “net positive requirements” “net positive frameworks” “net positive models” “net positive assessments” “net positive appraisals” “net positive evaluations” “net positive approaches” “net positive practices” “net positive tools” “net positive plans”)	8	11
	#39 year:[2023 TO 2024]	N/A	1
#40	(“positive outcome theories” “positive outcome concepts” “positive outcome principles” “positive outcome strategies” “positive outcome objectives” “positive outcome aims” “positive outcome goals” “positive outcome targets” “positive outcome requirements” “positive outcome frameworks” “positive outcome models” “positive outcome assessments” “positive outcome appraisals” “positive outcome evaluations” “positive outcome approaches” “positive outcome practices” “positive outcome tools” “positive outcome plans”)	7	7
	#40 year:[2023 TO 2024]	N/A	0
#41	(“net positive outcome theories” “net positive outcome concepts” “net positive outcome principles” “net positive outcome strategies” “net positive outcome objectives” “net positive outcome aims” “net positive outcome goals” “net positive outcome targets” “net positive outcome requirements” “net positive outcome frameworks” “net positive outcome models” “net positive outcome assessments” “net positive outcome appraisals” “net positive outcome evaluations” “net positive outcome approaches” “net positive outcome practices” “net positive outcome tools” “net positive outcome plans”)	0	0
#42	(“positive impact theories” “positive impact concepts” “positive impact principles” “positive impact strategies” “positive impact objectives” “positive impact aims” “positive impact goals” “positive impact targets” “positive impact requirements” “positive impact frameworks” “positive impact models” “positive impact assessments” “positive impact appraisals” “positive impact evaluations” “positive impact approaches” “positive impact practices” “positive impact tools” “positive impact plans”)	11	12
	#42 year:[2023 TO 2024]	N/A	1
#43	(“net positive impact theories” “net positive impact concepts” “net positive impact principles” “net positive impact strategies” “net positive impact objectives” “net positive impact aims” “net positive impact goals” “net positive impact targets” “net positive impact requirements” “net positive impact frameworks” “net positive impact models” “net positive impact assessments” “net positive impact appraisals” “net positive impact evaluations” “net positive impact approaches” “net positive impact practices” “net positive impact tools” “net positive impact plans”)	0	0
#44	(“positive development theories” “positive development concepts” “positive development principles” “positive development strategies” “positive development objectives” “positive development aims” “positive development goals” “positive development targets” “positive development requirements” “positive development frameworks” “positive development models” “positive development assessments” “positive development appraisals” “positive development evaluations” “positive development approaches” “positive development practices” “positive development tools” “positive development plans”)	15	23
	#44 year:[2023 TO 2024]	N/A	6
#45	(“regenerative design theories” “regenerative design concepts” “regenerative design principles” “regenerative design strategies” “regenerative design objectives” “regenerative design aims” “regenerative design goals” “regenerative design targets” “regenerative design requirements” “regenerative design frameworks” “regenerative design models” “regenerative design assessments” “regenerative design appraisals” “regenerative design evaluations” “regenerative design approaches” “regenerative design practices” “regenerative design tools”“regenerative design plans”)	67	81
	#45 year:[2023 TO 2024]	N/A	26
#46	(“regenerative development theories” “regenerative development concepts” “regenerative development principles” “regenerative development strategies” “regenerative development objectives” “regenerative development aims” “regenerative development goals” “regenerative development targets” “regenerative development requirements” “regenerative development frameworks” “regenerative development models” “regenerative development assessments” “regenerative development appraisals” “regenerative development evaluations” “regenerative development approaches” “regenerative development practices” “regenerative development tools” “regenerative development plans”)	25	30
	#46 year:[2023 TO 2024]	N/A	6
#47	(“regenerative sustainability theories” “regenerative sustainability concepts” “regenerative sustainability principles” “regenerative sustainability strategies” “regenerative sustainability objectives” “regenerative sustainability aims” “regenerative sustainability goals” “regenerative sustainability targets” “regenerative sustainability requirements” “regenerative sustainability frameworks” “regenerative sustainability models” “regenerative sustainability assessments” “regenerative sustainability appraisals” “regenerative sustainability evaluations” “regenerative sustainability approaches” “regenerative sustainability practices” “regenerative sustainability tools” “regenerative sustainability plans”)	10	14
	#47 year:[2023 TO 2024]	N/A	5
#48	(“no net loss theories” “no net loss concepts” “no net loss principles” “no net loss strategies” “no net loss objectives” “no net loss aims” “no net loss goals” “no net loss targets” “no net loss requirements” “no net loss frameworks” “no net loss models” “no net loss assessments” “no net loss appraisals” “no net loss evaluations” “no net loss approaches” “no net loss practices” “no net loss tools” “no net loss plans”)	32	40
	#48 year:[2023 TO 2024]	N/A	5
#49	(“mitigation hierarchy theories” “mitigation hierarchy concepts” “mitigation hierarchy principles” “mitigation hierarchy strategies” “mitigation hierarchy objectives” “mitigation hierarchy aims” “mitigation hierarchy goals” “mitigation hierarchy targets” “mitigation hierarchy requirements” “mitigation hierarchy frameworks” “mitigation hierarchy models” “mitigation hierarchy assessments” “mitigation hierarchy appraisals” “mitigation hierarchy evaluations” “mitigation hierarchy approaches” “mitigation hierarchy practices” “mitigation hierarchy tools” “mitigation hierarchy plans”)	20	23
	#49 year:[2023 TO 2024]	N/A	3
#50	(“health net gain” “health net benefit” “health net improvement” “health net increase” “health net positive”)	6	16
#51	(“health net gain” “health net benefit” “health net improvement” “health net increase” “health net positive”) subj:(theor* concept* principle* policy policies plan plans planning strategy strategies objective* aim* goal* target* requirement* framework* model* approach* practice* “health risk assessment” “impact assessment” “environmental assessment” “assessment tool” “assessment method” hia eia “sustainability appraisal” mitigation* “hierarchical system” “response hierarchy” compensation* offset* “off-set” tradeoff* “trade-off” reparation* restitution*)	0	5
	#51 year:[2023 TO 2024]	N/A	5
#52	(“wellbeing net gain” “wellbeing net benefit” “wellbeing net improvement” “wellbeing net increase” “wellbeing net positive”)	0	0
#53	(“wellbeing net gain” “wellbeing net benefit” “wellbeing net improvement” “wellbeing net increase” “wellbeing net positive”) subj:(theor* concept* principle* policy policies plan plans planning strategy strategies objective* aim* goal* target* requirement* framework* model* approach* practice* “health risk assessment” “impact assessment” “environmental assessment” “assessment tool” “assessment method” hia eia “sustainability appraisal” mitigation* “hierarchical system” “response hierarchy” compensation* offset* “off-set” tradeoff* “trade-off” reparation* restitution*)	0	0
#54	(“well-being net gain” “well-being net benefit” “well-being net improvement” “well-being net increase” “well-being net positive”)	0	0
#55	(“well-being net gain” “well-being net benefit” “well-being net improvement” “well-being net increase” “well-being net positive”) subj:(theor* concept* principle* policy policies plan plans planning strategy strategies objective* aim* goal* target* requirement* framework* model* approach* practice* “health risk assessment” “impact assessment” “environmental assessment” “assessment tool” “assessment method” hia eia “sustainability appraisal” mitigation* “hierarchical system” “response hierarchy” compensation* offset* “off-set” tradeoff* “trade-off” reparation* restitution*)	0	0
#56	(“welfare net gain” “welfare net benefit” “welfare net improvement” “welfare net increase” “welfare net positive”)	2	2
#57	(“welfare net gain” “welfare net benefit” “welfare net improvement” “welfare net increase” “welfare net positive”) subj:(theor* concept* principle* policy policies plan plans planning strategy strategies objective* aim* goal* target* requirement* framework* model* approach* practice* “health risk assessment” “impact assessment” “environmental assessment” “assessment tool” “assessment method” hia eia “sustainability appraisal” mitigation* “hierarchical system” “response hierarchy” compensation* offset* “off-set” tradeoff* “trade-off” reparation* restitution*)	0	0
#58	(“social net gain” “social net benefit” “social net improvement” “social net increase” “social net positive”)	52	60
#59	(“social net gain” “social net benefit” “social net improvement” “social net increase” “social net positive”) subj:(theor* concept* principle* policy policies plan plans planning strategy strategies objective* aim* goal* target* requirement* framework* model* approach* practice* “health risk assessment” “impact assessment” “environmental assessment” “assessment tool” “assessment method” hia eia “sustainability appraisal” mitigation* “hierarchical system” “response hierarchy” compensation* offset* “off-set” tradeoff* “trade-off” reparation* restitution*)	9	8
	#59 year:[2023 TO 2024]	N/A	0
#60	(“societal net gain” “societal net benefit” “societal net improvement” “societal net increase” “societal net positive”)	10	12
#61	(“societal net gain” “societal net benefit” “societal net improvement” “societal net increase” “societal net positive”) subj:(theor* concept* principle* policy policies plan plans planning strategy strategies objective* aim* goal* target* requirement* framework* model* approach* practice* “health risk assessment” “impact assessment” “environmental assessment” “assessment tool” “assessment method” hia eia “sustainability appraisal” mitigation* “hierarchical system” “response hierarchy” compensation* offset* “off-set” tradeoff* “trade-off” reparation* restitution*)	1	2
	#61 year:[2023 TO 2024]	N/A	0
#62	(“society net gain” “society net benefit” “society net improvement” “society net increase” “society net positive”)	0	0
#63	(“society net gain” “society net benefit” “society net improvement” “society net increase” “society net positive”) subj:(theor* concept* principle* policy policies plan plans planning strategy strategies objective* aim* goal* target* requirement* framework* model* approach* practice* “health risk assessment” “impact assessment” “environmental assessment” “assessment tool” “assessment method” hia eia “sustainability appraisal” mitigation* “hierarchical system” “response hierarchy” compensation* offset* “off-set” tradeoff* “trade-off” reparation* restitution*)	0	0
#64	(“societies net gain” “societies net benefit” “societies net improvement” “societies net increase” “societies net positive”)	0	0
#65	(“societies net gain” “societies net benefit” “societies net improvement” “societies net increase” “societies net positive”) subj:(theor* concept* principle* policy policies plan plans planning strategy strategies objective* aim* goal* target* requirement* framework* model* approach* practice* “health risk assessment” “impact assessment” “environmental assessment” “assessment tool” “assessment method” hia eia “sustainability appraisal” mitigation* “hierarchical system” “response hierarchy” compensation* offset* “off-set” tradeoff* “trade-off” reparation* restitution*)	0	0
#66	(“sociodemographic net gain” “sociodemographic net benefit” “sociodemographic net improvement” “sociodemographic net increase” “sociodemographic net positive”)	0	0
#67	(“sociodemographic net gain” “sociodemographic net benefit” “sociodemographic net improvement” “sociodemographic net increase” “sociodemographic net positive”) subj:(theor* concept* principle* policy policies plan plans planning strategy strategies objective* aim* goal* target* requirement* framework* model* approach* practice* “health risk assessment” “impact assessment” “environmental assessment” “assessment tool” “assessment method” hia eia “sustainability appraisal” mitigation* “hierarchical system” “response hierarchy” compensation* offset* “off-set” tradeoff* “trade-off” reparation* restitution*)	0	0
#68	(“socioeconomic net gain” “socioeconomic net benefit” “socioeconomic net improvement” “socioeconomic net increase” “socioeconomic net positive”)	2	2
#69	(“socioeconomic net gain” “socioeconomic net benefit” “socioeconomic net improvement” “socioeconomic net increase” “socioeconomic net positive”) subj:(theor* concept* principle* policy policies plan plans planning strategy strategies objective* aim* goal* target* requirement* framework* model* approach* practice* “health risk assessment” “impact assessment” “environmental assessment” “assessment tool” “assessment method” hia eia “sustainability appraisal” mitigation* “hierarchical system” “response hierarchy” compensation* offset* “off-set” tradeoff* “trade-off” reparation* restitution*)	0	0
#70	(“sociological net gain” “sociological net benefit” “sociological net improvement” “sociological net increase” “sociological net positive”)	0	0
#71	(“sociological net gain” “sociological net benefit” “sociological net improvement” “sociological net increase” “sociological net positive”) subj:(theor* concept* principle* policy policies plan plans planning strategy strategies objective* aim* goal* target* requirement* framework* model* approach* practice* “health risk assessment” “impact assessment” “environmental assessment” “assessment tool” “assessment method” hia eia “sustainability appraisal” mitigation* “hierarchical system” “response hierarchy” compensation* offset* “off-set” tradeoff* “trade-off” reparation* restitution*)	0	0
#72	(“community net gain” “community net benefit” “community net improvement” “community net increase” “community net positive”)	1	1
#73	(“community net gain” “community net benefit” “community net improvement” “community net increase” “community net positive”) subj:(theor* concept* principle* policy policies plan plans planning strategy strategies objective* aim* goal* target* requirement* framework* model* approach* practice* “health risk assessment” “impact assessment” “environmental assessment” “assessment tool” “assessment method” hia eia “sustainability appraisal” mitigation* “hierarchical system” “response hierarchy” compensation* offset* “off-set” tradeoff* “trade-off” reparation* restitution*)	0	0
#74	(“net health gain” “net health benefit” “net health improvement” “net health increase” “net health positive”)	315	456
#75	(“net health gain” “net health benefit” “net health improvement” “net health increase” “net health positive”) subj:(theor* concept* principle* policy policies plan plans planning strategy strategies objective* aim* goal* target* requirement* framework* model* approach* practice* “health risk assessment” “impact assessment” “environmental assessment” “assessment tool” “assessment method” hia eia “sustainability appraisal” mitigation* “hierarchical system” “response hierarchy” compensation* offset* “off-set” tradeoff* “trade-off” reparation* restitution*)	59	55
	#75 year:[2023 TO 2024]	N/A	13
#76	(“net wellbeing gain” “net wellbeing benefit” “net wellbeing improvement” “net wellbeing increase” “net wellbeing positive”)	0	0
#77	(“net wellbeing gain” “net wellbeing benefit” “net wellbeing improvement” “net wellbeing increase” “net wellbeing positive”) subj:(theor* concept* principle* policy policies plan plans planning strategy strategies objective* aim* goal* target* requirement* framework* model* approach* practice* “health risk assessment” “impact assessment” “environmental assessment” “assessment tool” “assessment method” hia eia “sustainability appraisal” mitigation* “hierarchical system” “response hierarchy” compensation* offset* “off-set” tradeoff* “trade-off” reparation* restitution*)	0	0
#78	(“net well-being gain” “net well-being benefit” “net well-being improvement” “net well-being increase” “net well-being positive”)	0	0
#79	(“net well-being gain” “net well-being benefit” “net well-being improvement” “net well-being increase” “net well-being positive”) subj:(theor* concept* principle* policy policies plan plans planning strategy strategies objective* aim* goal* target* requirement* framework* model* approach* practice* “health risk assessment” “impact assessment” “environmental assessment” “assessment tool” “assessment method” hia eia “sustainability appraisal” mitigation* “hierarchical system” “response hierarchy” compensation* offset* “off-set” tradeoff* “trade-off” reparation* restitution*)	0	0
#80	(“net welfare gain” “net welfare benefit” “net welfare improvement” “net welfare increase” “net welfare positive”)	164	195
#81	(“net welfare gain” “net welfare benefit” “net welfare improvement” “net welfare increase” “net welfare positive”) subj:(theor* concept* principle* policy policies plan plans planning strategy strategies objective* aim* goal* target* requirement* framework* model* approach* practice* “health risk assessment” “impact assessment” “environmental assessment” “assessment tool” “assessment method” hia eia “sustainability appraisal” mitigation* “hierarchical system” “response hierarchy” compensation* offset* “off-set” tradeoff* “trade-off” reparation* restitution*)	29	32
	#81 year:[2023 TO 2024]	N/A	8
#82	(“net social gain” “net social benefit” “net social improvement” “net social increase” “net social positive”)	366	425
#83	(“net social gain” “net social benefit” “net social improvement” “net social increase” “net social positive”) subj:(theor* concept* principle* policy policies plan plans planning strategy strategies objective* aim* goal* target* requirement* framework* model* approach* practice* “health risk assessment” “impact assessment” “environmental assessment” “assessment tool” “assessment method” hia eia “sustainability appraisal” mitigation* “hierarchical system” “response hierarchy” compensation* offset* “off-set” tradeoff* “trade-off” reparation* restitution*)	71	75
	#83 year:[2023 TO 2024]	N/A	6
#84	(“net societal gain” “net societal benefit” “net societal improvement” “net societal increase” “net societal positive”)	39	60
#85	(“net societal gain” “net societal benefit” “net societal improvement” “net societal increase” “net societal positive”) subj:(theor* concept* principle* policy policies plan plans planning strategy strategies objective* aim* goal* target* requirement* framework* model* approach* practice* “health risk assessment” “impact assessment” “environmental assessment” “assessment tool” “assessment method” hia eia “sustainability appraisal” mitigation* “hierarchical system” “response hierarchy” compensation* offset* “off-set” tradeoff* “trade-off” reparation* restitution*)	6	6
	#85 year:[2023 TO 2024]	N/A	0
#86	(“net society gain” “net society benefit” “net society improvement” “net society increase” “net society positive”)	3	4
#87	(“net society gain” “net society benefit” “net society improvement” “net society increase” “net society positive”) subj:(theor* concept* principle* policy policies plan plans planning strategy strategies objective* aim* goal* target* requirement* framework* model* approach* practice* “health risk assessment” “impact assessment” “environmental assessment” “assessment tool” “assessment method” hia eia “sustainability appraisal” mitigation* “hierarchical system” “response hierarchy” compensation* offset* “off-set” tradeoff* “trade-off” reparation* restitution*)	0	0
#88	(“net societies gain” “net societies benefit” “net societies improvement” “net societies increase” “net societies positive”)	0	0
#89	(“net societies gain” “net societies benefit” “net societies improvement” “net societies increase” “net societies positive”) subj:(theor* concept* principle* policy policies plan plans planning strategy strategies objective* aim* goal* target* requirement* framework* model* approach* practice* “health risk assessment” “impact assessment” “environmental assessment” “assessment tool” “assessment method” hia eia “sustainability appraisal” mitigation* “hierarchical system” “response hierarchy” compensation* offset* “off-set” tradeoff* “trade-off” reparation* restitution*)	0	0
#90	(“net sociodemographic gain” “net sociodemographic benefit” “net sociodemographic improvement” “net sociodemographic increase” “net sociodemographic positive”)	0	0
#91	(“net sociodemographic gain” “net sociodemographic benefit” “net sociodemographic improvement” “net sociodemographic increase” “net sociodemographic positive”) subj:(theor* concept* principle* policy policies plan plans planning strategy strategies objective* aim* goal* target* requirement* framework* model* approach* practice* “health risk assessment” “impact assessment” “environmental assessment” “assessment tool” “assessment method” hia eia “sustainability appraisal” mitigation* “hierarchical system” “response hierarchy” compensation* offset* “off-set” tradeoff* “trade-off” reparation* restitution*)	0	0
#92	(“net socioeconomic gain” “net socioeconomic benefit” “net socioeconomic improvement” “net socioeconomic increase” “net socioeconomic positive”)	3	7
#93	(“net socioeconomic gain” “net socioeconomic benefit” “net socioeconomic improvement” “net socioeconomic increase” “net socioeconomic positive”) subj:(theor* concept* principle* policy policies plan plans planning strategy strategies objective* aim* goal* target* requirement* framework* model* approach* practice* “health risk assessment” “impact assessment” “environmental assessment” “assessment tool” “assessment method” hia eia “sustainability appraisal” mitigation* “hierarchical system” “response hierarchy” compensation* offset* “off-set” tradeoff* “trade-off” reparation* restitution*)	1	1
	#93 year:[2023 TO 2024]	N/A	0
#94	(“net sociological gain” “net sociological benefit” “net sociological improvement” “net sociological increase” “net sociological positive”)	0	0
#95	(“net sociological gain” “net sociological benefit” “net sociological improvement” “net sociological increase” “net sociological positive”) subj:(theor* concept* principle* policy policies plan plans planning strategy strategies objective* aim* goal* target* requirement* framework* model* approach* practice* “health risk assessment” “impact assessment” “environmental assessment” “assessment tool” “assessment method” hia eia “sustainability appraisal” mitigation* “hierarchical system” “response hierarchy” compensation* offset* “off-set” tradeoff* “trade-off” reparation* restitution*)	0	0
#96	(“net community gain” “net community benefit” “net community improvement” “net community increase” “net community positive”)	8	9
#97	(“net community gain” “net community benefit” “net community improvement” “net community increase” “net community positive”) subj:(theor* concept* principle* policy policies plan plans planning strategy strategies objective* aim* goal* target* requirement* framework* model* approach* practice* “health risk assessment” “impact assessment” “environmental assessment” “assessment tool” “assessment method” hia eia “sustainability appraisal” mitigation* “hierarchical system” “response hierarchy” compensation* offset* “off-set” tradeoff* “trade-off” reparation* restitution*)	2	1
	#97 year:[2023 TO 2024]	N/A	0
	**Total records retrieved (sum)**	**3,948**	**4,799**
	**Total records retrieved (sum) year:[2023 TO 2024]**	**N/A**	**746**

**Table:**

Embase (Ovid)			
**Searches conducted: August 17, 2023; July 12, 2024**			
**Search**	**Query**	**Records retrieved (17/08/23)**	**Records retrieved 12/07/24)**
#1	(“net outcome*” or “net gain*” or “net positive outcome*” or “net positive impact*” or “no net loss*” or “mitigation hierarch*” or “regenerative design*” or “regenerative development*” or “regenerative sustainability”).mp.	1830	1933
#2	(“net positive*” or “net benefit*” or “positive development*”).hw,kf.	276	295
#3	1 or 2	2102	2224
#4	exp theory/ or conceptual framework/ or theoretical model/ or concept formation/ or management/ or exp planning/ or professional practice/ or practice guideline/	1,475,080	1,537,197
#5	(theor* or concept* or principle* or policy or policies or plan or plans or planning or strategy or strategies or objective* or aim* or goal* or target* or requirement* or framework* or model* or approach* or practice*).hw,kf.	6,956,739	7,330,475
#6	health impact assessment/ or health risk assessment/ or environmental impact assessment/	27,775	30,093
#7	(“health risk assessment*” or “impact assessment*” or “environmental assessment*” or “assessment tool*” or “assessment method*”).mp.	145,721	158,001
#8	(HIA or HIAs or EIA or EIAs or “sustainability appraisal*”).mp.	17,012	17,437
#9	exp mitigation/ or compensation/	24,128	27,824
#10	(mitigation* or “hierarchical system*” or “response hierarch*” or compensation* or offset* or “off-set*” or tradeoff* or “trade-off*” or reparation* or restitution*).mp.	221,144	239,292
#11	4 or 5 or 6 or 7 or 8 or 9 or 10	7,403,432	7,803,079
#12	((“net outcome*” or “net gain*” or “net benefit*” or “net improve*” or “net increase*” or “net positive*” or “positive outcome*” or “net positive outcome*” or “positive impact*” or “net positive impact*” or “positive development*” or “regenerative design*” or “regenerative development*” or “regenerative sustainability” or “no net loss*” or “mitigation hierarch*”) adj (theor* or concept* or principle* or polic* or plan* or strateg* or objective* or aim* or goal* or target* or requirement* or framework* or model* or assessment* or appraisal* or evaluation* or approach* or practice* or tool*)).mp.	208	219
#13	(3 and 11) or 12	861	924
#14	(health* or wellbeing or “well-being” or welfare).mp.	6,669,052	7,109,638
#15	exp health/ or public health/ or exp health planning/ or health equity/ or exp wellbeing/ or quality of life/ or exp welfare/	1,841,266	1,975,779
#16	exp “social aspects and related phenomena”/ or exp social justice/ or restorative justice/ or sociodemographics/ or society/	4,419,779	4,654,458
#17	(social* or societal* or society or societies or sociodemographic* or socioeconomic* or sociological*).hw,kf.	2,124,671	2,212,470
#18	exp human/ or exp population/ or population health management/ or advocacy group/	25,543,606	26,885,557
#19	(human* or population* or stakeholder* or community or communities).hw,kf.	26,043,913	27,407,629
#20	14 or 15 or 16 or 17 or 18 or 19	27,512,434	28,927,447
#21	3 and 20	1455	1549
#22	((health* or wellbeing or “well-being” or welfare or soci* or commun*) adj3 net adj3 (gain* or benefit* or improve* or increase* or positive*)).mp.	933	998
#23	11 and 22	436	459
#24	(“health net gain*” or “net health gain*” or “wellbeing net gain*” or “net wellbeing gain*” or “well-being net gain*” or “net well-being gain*”).mp.	31	33
#25	13 or 21 or 23 or 24	2274	2412
#26	limit 25 to dc=20230817-20240712	N/A	156

**Table:**

MEDLINE (Ovid)			
**Searches conducted: August 17, 2023; July 12, 2024**			
**Search**	**Query**	**Records retrieved (17/08/23)**	Records retrieved (12/07/24)
#1	(“net outcome*” or “net gain*” or “net positive outcome*” or “net positive impact*” or “no net loss*” or “mitigation hierarch*” or “regenerative design*” or “regenerative development*” or “regenerative sustainability”).mp.	1674	1755
#2	(“net positive*” or “net benefit*” or “positive development*”).hw,kf.	166	183
#3	1 or 2	1839	1937
#4	models, theoretical/ or concept formation/ or exp principle-based ethics/ or exp policy/ or policy making/ or exp social planning/ or professional practice/ or practice guideline/	460,455	468,874
#5	(theor* or concept* or principle* or policy or policies or plan or plans or planning or strategy or strategies or objective* or aim* or goal* or target* or requirement* or framework* or model* or approach* or practice*).hw,kf.	3,441,537	3,552,190
#6	health impact assessment/	945	974
#7	(“health risk assessment*” or “impact assessment*” or “environmental assessment*” or “assessment tool*” or “assessment method*”).mp.	67,702	75,392
#8	(HIA or HIAs or EIA or EIAs or “sustainability appraisal*”).mp.	11,932	12,127
#9	“risk evaluation and mitigation”/ or “compensation and redress”/	3212	3232
#10	(mitigation* or “hierarchical system*” or “response hierarch*” or compensation* or offset* or “off-set*” or tradeoff* or “trade-off*” or reparation* or restitution*).mp.	193,777	208,783
#11	4 or 5 or 6 or 7 or 8 or 9 or 10	3,727,624	3,859,309
#12	((“net outcome*” or “net gain*” or “net benefit*” or “net improve*” or “net increase*” or “net positive*” or “positive outcome*” or “net positive outcome*” or “positive impact*” or “net positive impact*” or “positive development*” or “regenerative design*” or “regenerative development*” or “regenerative sustainability” or “no net loss*” or “mitigation hierarch*”) adj (theor* or concept* or principle* or polic* or plan* or strateg* or objective* or aim* or goal* or target* or requirement* or framework* or model* or assessment* or appraisal* or evaluation* or approach* or practice* or tool*)).mp.	123	130
#13	(3 and 11) or 12	581	615
#14	(health* or wellbeing or “well-being” or welfare).mp.	4,761,121	5,051,868
#15	exp health/ or exp public health/ or exp health status/ or exp health planning/ or health equity/ or exp social welfare/	9,618,315	9,953,263
#16	social problems/ or exp social justice/ or exp sociological factors/ or exp socioeconomic factors/	946,501	974,399
#17	(social* or societal* or society or societies or sociodemographic* or socioeconomic* or sociological*).hw,kf.	816,365	841,855
#18	humans/ or persons/ or exp population/ or population health management/	21,444,362	22,115,946
#19	(human* or population* or stakeholder* or community or communities).hw,kf.	21,645,926	22,331,263
#20	14 or 15 or 16 or 17 or 18 or 19	23,942,098	24,757,941
#21	3 and 20	1105	1163
#22	((health* or wellbeing or “well-being” or welfare or soci* or commun*) adj3 net adj3 (gain* or benefit* or improve* or increase* or positive*)).mp.	675	734
#23	11 and 22	270	283
#24	(“health net gain*” or “net health gain*” or “wellbeing net gain*” or “net wellbeing gain*” or “well-being net gain*” or “net well-being gain*”).mp.	25	28
#25	13 or 21 or 23 or 24	1647	1735
#26	limit 25 to dt=20230817-20240712	N/A	81

**Table:**

TRIP Pro			
**Searches conducted: August 17, 2023; July 12, 2024**			
**Search**	**Query**	**Records retrieved (17/08/23)**	**Records retrieved (12/07/24)**
#1	(“net outcome” OR “net gain” OR “net positive outcome” OR “net positive impact” OR “no net loss” OR “mitigation hierarchy” OR “regenerative design” OR “regenerative development” OR “regenerative sustainability”)	869	911
#2	(theor* OR concept* OR principle* OR policy OR policies OR plan OR plans OR planning OR strategy OR strategies OR objective* OR aim* OR goal* OR target* OR requirement* OR framework* OR model * OR approach* OR practice*)	2,645,459	3,036,636
#3	(“health risk assessment” OR “impact assessment” OR “environmental assessment” OR “assessment tool” OR “assessment method”)	34,230	42,396
#4	(hia OR eia OR “sustainability appraisal”)	2,795	2,844
#5	(mitigation* OR “hierarchical system” OR “response hierarchy” OR compensation* OR offset* OR “off set” OR tradeoff* OR “trade off” OR reparation* OR restitution*)	22,133	23,338
#6	(theor* OR concept* OR principle* OR policy OR policies OR plan OR plans OR planning OR strategy OR strategies OR objective* OR aim* OR goal* OR target* OR requirement* OR framework* OR model * OR approach* OR practice* OR “health risk assessment” OR “impact assessment” OR “environmental assessment” OR “assessment tool” OR “assessment method” OR hia OR eia OR “sustainability appraisal” OR mitigation* OR “hierarchical system” OR “response hierarchy” OR compensation* OR offset* OR “off set” OR tradeoff* OR “trade off” OR reparation* OR restitution*)	2,645,459	3,052,648
#7	(“net outcome” OR “net gain” OR “net positive outcome” OR “net positive impact” OR “no net loss” OR “mitigation hierarchy” OR “regenerative design” OR “regenerative development” OR “regenerative sustainability”) AND (theor* OR concept* OR principle* OR policy OR policies OR plan OR plans OR planning OR strategy OR strategies OR objective* OR aim* OR goal* OR target* OR requirement* OR framework* OR model * OR approach* OR practice* OR “health risk assessment” OR “impact assessment” OR “environmental assessment” OR “assessment tool” OR “assessment method” OR hia OR eia OR “sustainability appraisal” OR mitigation* OR “hierarchical system” OR “response hierarchy” OR compensation* OR offset* OR “off set” OR tradeoff* OR “trade off” OR reparation* OR restitution*)	529	588
	#7 from_date:2023 to_date:2024	N/A	43
#8	(health* OR wellbeing OR “well being” OR welfare)	1,364,296	1,461,297
#9	(social* OR societal* OR society OR societies OR sociodemographic* OR socioeconomic* OR sociological*)	364,192	391,476
#10	(human* OR population* OR stakeholder* OR community OR communities)	1,195,021	1,281,821
#11	(health* OR wellbeing OR “well being” OR welfare OR social* OR societal* OR society OR societies OR sociodemographic* OR socioeconomic* OR sociological* OR human* OR population* OR stakeholder* OR community OR communities)	2,074,864	2,228,198
#12	(“net outcome” OR “net gain” OR “net positive outcome” OR “net positive impact” OR “no net loss” OR “mitigation hierarchy” OR “regenerative design” OR “regenerative development” OR “regenerative sustainability”) AND (health* OR wellbeing OR “well being” OR welfare OR social* OR societal* OR society OR societies OR sociodemographic* OR socioeconomic* OR sociological* OR human* OR population* OR stakeholder* OR community OR communities)	473	503
	#12 from_date:2023 to_date:2024	N/A	37
#13	(“health net gain” or “net health gain” or “wellbeing net gain” or “net wellbeing gain” or “well being net gain” or “net well being gain”)	17	20
	#13 from_date:2023 to_date:2024	N/A	4
#14	(“net gain theory” or “net gain concept” or “net gain principle” or “net gain policy” or “net gain policies” or “net gain strategy” or “net gain objective” or “net gain aim” or “net gain goal” or “net gain target” or “net gain requirement” or “net gain framework” or “net gain model” or “net gain assessment” or “net gain appraisal” or “net gain evaluation” or “net gain approach” or “net gain practice” or “net gain tool” or “net gain theories” or “net gain concepts” or “net gain principles” or “net gain strategies” or “net gain objectives” or “net gain aims” or “net gain goals” or “net gain targets” or “net gain requirements” or “net gain frameworks” or “net gain models” or “net gain assessments” or “net gain appraisals” or “net gain evaluations” or “net gain approaches” or “net gain practices” or “net gain tools” or “net gain plan” or “net gain plans” or “net gain planning”)	2	3
	#14 from_date:2023 to_date:2024	N/A	1
#15	(“net outcome theory” or “net outcome concept” or “net outcome principle” or “net outcome policy” or “net outcome policies” or “net outcome strategy” or “net outcome objective” or “net outcome aim” or “net outcome goal” or “net outcome target” or “net outcome requirement” or “net outcome framework” or “net outcome model” or “net outcome assessment” or “net outcome appraisal” or “net outcome evaluation” or “net outcome approach” or “net outcome practice” or “net outcome tool” or “net outcome theories” or “net outcome concepts” or “net outcome principles” or “net outcome strategies” or “net outcome objectives” or “net outcome aims” or “net outcome goals” or “net outcome targets” or “net outcome requirements” or “net outcome frameworks” or “net outcome models” or “net outcome assessments” or “net outcome appraisals” or “net outcome evaluations” or “net outcome approaches” or “net outcome practices” or “net outcome tools” or “net outcome plan” or “net outcome plans” or “net outcome planning”)	1	1
	#15 from_date:2023 to_date:2024	N/A	0
#16	(“net benefit theory” or “net benefit concept” or “net benefit principle” or “net benefit policy” or “net benefit policies” or “net benefit strategy” or “net benefit objective” or “net benefit aim” or “net benefit goal” or “net benefit target” or “net benefit requirement” or “net benefit framework” or “net benefit model” or “net benefit assessment” or “net benefit appraisal” or “net benefit evaluation” or “net benefit approach” or “net benefit practice” or “net benefit tool” or “net benefit theories” or “net benefit concepts” or “net benefit principles” or “net benefit strategies” or “net benefit objectives” or “net benefit aims” or “net benefit goals” or “net benefit targets” or “net benefit requirements” or “net benefit frameworks” or “net benefit models” or “net benefit assessments” or “net benefit appraisals” or “net benefit evaluations” or “net benefit approaches” or “net benefit practices” or “net benefit tools” or “net benefit plan” or “net benefit plans” or “net benefit planning”)	70	74
	#16 from_date:2023 to_date:2024	N/A	4
#17	(“net improvement theory” or “net improvement concept” or “net improvement principle” or “net improvement policy” or “net improvement policies” or “net improvement strategy” or “net improvement objective” or “net improvement aim” or “net improvement goal” or “net improvement target” or “net improvement requirement” or “net improvement framework” or “net improvement model” or “net improvement assessment” or “net improvement appraisal” or “net improvement evaluation” or “net improvement approach” or “net improvement practice” or “net improvement tool” or “net improvement theories” or “net improvement concepts” or “net improvement principles” or “net improvement strategies” or “net improvement objectives” or “net improvement aims” or “net improvement goals” or “net improvement targets” or “net improvement requirements” or “net improvement frameworks” or “net improvement models” or “net improvement assessments” or “net improvement appraisals” or “net improvement evaluations” or “net improvement approaches” or “net improvement practices” or “net improvement tools” or “net improvement plan” or “net improvement plans” or “net improvement planning”)	1	1
	#17 from_date:2023 to_date:2024	N/A	0
#18	(“net increase theory” or “net increase concept” or “net increase principle” or “net increase policy” or “net increase policies” or “net increase strategy” or “net increase objective” or “net increase aim” or “net increase goal” or “net increase target” or “net increase requirement” or “net increase framework” or “net increase model” or “net increase assessment” or “net increase appraisal” or “net increase evaluation” or “net increase approach” or “net increase practice” or “net increase tool” or “net increase theories” or “net increase concepts” or “net increase principles” or “net increase strategies” or “net increase objectives” or “net increase aims” or “net increase goals” or “net increase targets” or “net increase requirements” or “net increase frameworks” or “net increase models” or “net increase assessments” or “net increase appraisals” or “net increase evaluations” or “net increase approaches” or “net increase practices” or “net increase tools” or “net increase plan” or “net increase plans” or “net increase planning”)	0	0
#19	(“net positive theory” or “net positive concept” or “net positive principle” or “net positive policy” or “net positive policies” or “net positive strategy” or “net positive objective” or “net positive aim” or “net positive goal” or “net positive target” or “net positive requirement” or “net positive framework” or “net positive model” or “net positive assessment” or “net positive appraisal” or “net positive evaluation” or “net positive approach” or “net positive practice” or “net positive tool” or “net positive theories” or “net positive concepts” or “net positive principles” or “net positive strategies” or “net positive objectives” or “net positive aims” or “net positive goals” or “net positive targets” or “net positive requirements” or “net positive frameworks” or “net positive models” or “net positive assessments” or “net positive appraisals” or “net positive evaluations” or “net positive approaches” or “net positive practices” or “net positive tools” or “net positive plan” or “net positive plans” or “net positive planning”)	1	1
	#19 from_date:2023 to_date:2024	N/A	0
#20	(“positive outcome theory” or “positive outcome concept” or “positive outcome principle” or “positive outcome policy” or “positive outcome policies” or “positive outcome strategy” or “positive outcome objective” or “positive outcome aim” or “positive outcome goal” or “positive outcome target” or “positive outcome requirement” or “positive outcome framework” or “positive outcome model” or “positive outcome assessment” or “positive outcome appraisal” or “positive outcome evaluation” or “positive outcome approach” or “positive outcome practice” or “positive outcome tool” or “positive outcome theories” or “positive outcome concepts” or “positive outcome principles” or “positive outcome strategies” or “positive outcome objectives” or “positive outcome aims” or “positive outcome goals” or “positive outcome targets” or “positive outcome requirements” or “positive outcome frameworks” or “positive outcome models” or “positive outcome assessments” or “positive outcome appraisals” or “positive outcome evaluations” or “positive outcome approaches” or “positive outcome practices” or “positive outcome tools” or “positive outcome plan” or “positive outcome plans” or “positive outcome planning”)	24	24
	#20 from_date:2023 to_date:2024	N/A	1
#21	(“net positive outcome theory” or “net positive outcome concept” or “net positive outcome principle” or “net positive outcome policy” or “net positive outcome policies” or “net positive outcome strategy” or “net positive outcome objective” or “net positive outcome aim” or “net positive outcome goal” or “net positive outcome target” or “net positive outcome requirement” or “net positive outcome framework” or “net positive outcome model” or “net positive outcome assessment” or “net positive outcome appraisal” or “net positive outcome evaluation” or “net positive outcome approach” or “net positive outcome practice” or “net positive outcome tool” or “net positive outcome theories” or “net positive outcome concepts” or “net positive outcome principles” or “net positive outcome strategies” or “net positive outcome objectives” or “net positive outcome aims” or “net positive outcome goals” or “net positive outcome targets” or “net positive outcome requirements” or “net positive outcome frameworks” or “net positive outcome models” or “net positive outcome assessments” or “net positive outcome appraisals” or “net positive outcome evaluations” or “net positive outcome approaches” or “net positive outcome practices” or “net positive outcome tools” or “net positive outcome plan” or “net positive outcome plans” or “net positive outcome planning”)	0	0
#22	(“positive impact theory” or “positive impact concept” or “positive impact principle” or “positive impact policy” or “positive impact policies” or “positive impact strategy” or “positive impact objective” or “positive impact aim” or “positive impact goal” or “positive impact target” or “positive impact requirement” or “positive impact framework” or “positive impact model” or “positive impact assessment” or “positive impact appraisal” or “positive impact evaluation” or “positive impact approach” or “positive impact practice” or “positive impact tool” or “positive impact theories” or “positive impact concepts” or “positive impact principles” or “positive impact strategies” or “positive impact objectives” or “positive impact aims” or “positive impact goals” or “positive impact targets” or “positive impact requirements” or “positive impact frameworks” or “positive impact models” or “positive impact assessments” or “positive impact appraisals” or “positive impact evaluations” or “positive impact approaches” or “positive impact practices” or “positive impact tools” or “positive impact plan” or “positive impact plans” or “positive impact planning”)	7	7
	#22 from_date:2023 to_date:2024	N/A	0
#23	(“net positive impact theory” or “net positive impact concept” or “net positive impact principle” or “net positive impact policy” or “net positive impact policies” or “net positive impact strategy” or “net positive impact objective” or “net positive impact aim” or “net positive impact goal” or “net positive impact target” or “net positive impact requirement” or “net positive impact framework” or “net positive impact model” or “net positive impact assessment” or “net positive impact appraisal” or “net positive impact evaluation” or “net positive impact approach” or “net positive impact practice” or “net positive impact tool” or “net positive impact theories” or “net positive impact concepts” or “net positive impact principles” or “net positive impact strategies” or “net positive impact objectives” or “net positive impact aims” or “net positive impact goals” or “net positive impact targets” or “net positive impact requirements” or “net positive impact frameworks” or “net positive impact models” or “net positive impact assessments” or “net positive impact appraisals” or “net positive impact evaluations” or “net positive impact approaches” or “net positive impact practices” or “net positive impact tools” or “net positive impact plan” or “net positive impact plans” or “net positive impact planning”)	0	0
#24	(“positive development theory” or “positive development concept” or “positive development principle” or “positive development policy” or “positive development policies” or “positive development strategy” or “positive development objective” or “positive development aim” or “positive development goal” or “positive development target” or “positive development requirement” or “positive development framework” or “positive development model” or “positive development assessment” or “positive development appraisal” or “positive development evaluation” or “positive development approach” or “positive development practice” or “positive development tool” or “positive development theories” or “positive development concepts” or “positive development principles” or “positive development strategies” or “positive development objectives” or “positive development aims” or “positive development goals” or “positive development targets” or “positive development requirements” or “positive development frameworks” or “positive development models” or “positive development assessments” or “positive development appraisals” or “positive development evaluations” or “positive development approaches” or “positive development practices” or “positive development tools” or “positive development plan” or “positive development plans” or “positive development planning”)	3	4
	#24 from_date:2023 to_date:2024	N/A	1
#25	(“regenerative design theory” or “regenerative design concept” or “regenerative design principle” or “regenerative design policy” or “regenerative design policies” or “regenerative design strategy” or “regenerative design objective” or “regenerative design aim” or “regenerative design goal” or “regenerative design target” or “regenerative design requirement” or “regenerative design framework” or “regenerative design model” or “regenerative design assessment” or “regenerative design appraisal” or “regenerative design evaluation” or “regenerative design approach” or “regenerative design practice” or “regenerative design tool” or “regenerative design theories” or “regenerative design concepts” or “regenerative design principles” or “regenerative design strategies” or “regenerative design objectives” or “regenerative design aims” or “regenerative design goals” or “regenerative design targets” or “regenerative design requirements” or “regenerative design frameworks” or “regenerative design models” or “regenerative design assessments” or “regenerative design appraisals” or “regenerative design evaluations” or “regenerative design approaches” or “regenerative design practices” or “regenerative design tools” or “regenerative design plan” or “regenerative design plans” or “regenerative design planning”)	0	0
#26	(“regenerative development theory” or “regenerative development concept” or “regenerative development principle” or “regenerative development policy” or “regenerative development policies” or “regenerative development strategy” or “regenerative development objective” or “regenerative development aim” or “regenerative development goal” or “regenerative development target” or “regenerative development requirement” or “regenerative development framework” or “regenerative development model” or “regenerative development assessment” or “regenerative development appraisal” or “regenerative development evaluation” or “regenerative development approach” or “regenerative development practice” or “regenerative development tool” or “regenerative development theories” or “regenerative development concepts” or “regenerative development principles” or “regenerative development strategies” or “regenerative development objectives” or “regenerative development aims” or “regenerative development goals” or “regenerative development targets” or “regenerative development requirements” or “regenerative development frameworks” or “regenerative development models” or “regenerative development assessments” or “regenerative development appraisals” or “regenerative development evaluations” or “regenerative development approaches” or “regenerative development practices” or “regenerative development tools”or “regenerative development plan” or “regenerative development plans” or “regenerative development planning”)	0	0
#27	(“regenerative sustainability theory” OR “regenerative sustainability concept” OR “regenerative sustainability principle” OR “regenerative sustainability policy” OR “regenerative sustainability policies” OR “regenerative sustainability strategy” OR “regenerative sustainability objective” OR “regenerative sustainability aim” OR “regenerative sustainability goal” OR “regenerative sustainability target” OR “regenerative sustainability requirement” OR “regenerative sustainability framework” OR “regenerative sustainability model” OR “regenerative sustainability assessment” OR “regenerative sustainability appraisal” OR “regenerative sustainability evaluation” OR “regenerative sustainability approach” OR “regenerative sustainability practice” OR “regenerative sustainability tool” OR “regenerative sustainability theories” OR “regenerative sustainability concepts” OR “regenerative sustainability principles” OR “regenerative sustainability strategies” OR “regenerative sustainability objectives” OR “regenerative sustainability aims” OR “regenerative sustainability goals” OR “regenerative sustainability targets” OR “regenerative sustainability requirements” OR “regenerative sustainability frameworks” OR “regenerative sustainability models” OR “regenerative sustainability assessments” OR “regenerative sustainability appraisals” OR “regenerative sustainability evaluations” OR “regenerative sustainability approaches” OR “regenerative sustainability practices” OR “regenerative sustainability tools” OR “regenerative sustainability plan” OR “regenerative sustainability plans” or “regenerative sustainability planning”)	0	0
#28	(“no net loss theory” or “no net loss concept” or “no net loss principle” or “no net loss policy” or “no net loss policies” or “no net loss strategy” or “no net loss objective” or “no net loss aim” or “no net loss goal” or “no net loss target” or “no net loss requirement” or “no net loss framework” or “no net loss model” or “no net loss assessment” or “no net loss appraisal” or “no net loss evaluation” or “no net loss approach” or “no net loss practice” or “no net loss tool” or “no net loss theories” or “no net loss concepts” or “no net loss principles” or “no net loss strategies” or “no net loss objectives” or “no net loss aims” or “no net loss goals” or “no net loss targets” or “no net loss requirements” or “no net loss frameworks” or “no net loss models” or “no net loss assessments” or “no net loss appraisals” or “no net loss evaluations” or “no net loss approaches” or “no net loss practices” or “no net loss tools” or “no net loss plan” or “no net loss plans” or “no net loss planning”)	2	2
	#28 from_date:2023 to_date:2024	N/A	0
#29	(“mitigation hierarchy theory” or “mitigation hierarchy concept” or “mitigation hierarchy principle” or “mitigation hierarchy policy” or “mitigation hierarchy policies” or “mitigation hierarchy strategy” or “mitigation hierarchy objective” or “mitigation hierarchy aim” or “mitigation hierarchy goal” or “mitigation hierarchy target” or “mitigation hierarchy requirement” or “mitigation hierarchy framework” or “mitigation hierarchy model” or “mitigation hierarchy assessment” or “mitigation hierarchy appraisal” or “mitigation hierarchy evaluation” or “mitigation hierarchy approach” or “mitigation hierarchy practice” or “mitigation hierarchy tool” or “mitigation hierarchy theories” or “mitigation hierarchy concepts” or “mitigation hierarchy principles” or “mitigation hierarchy strategies” or “mitigation hierarchy objectives” or “mitigation hierarchy aims” or “mitigation hierarchy goals” or “mitigation hierarchy targets” or “mitigation hierarchy requirements” or “mitigation hierarchy frameworks” or “mitigation hierarchy models” or “mitigation hierarchy assessments” or “mitigation hierarchy appraisals” or “mitigation hierarchy evaluations” or “mitigation hierarchy approaches” or “mitigation hierarchy practices” or “mitigation hierarchy tools” or “mitigation hierarchy plan” or “mitigation hierarchy plans” or “mitigation hierarchy planning”)	0	0
#30	(“health net gain” or “health net benefit” or “health net improvement” or “health net increase” or “health net positive”)	2	3
#31	(“health net gain” or “health net benefit” or “health net improvement” or “health net increase” or “health net positive”) and (theor* OR concept* OR principle* OR policy OR policies OR plan OR plans OR planning OR strategy OR strategies OR objective* OR aim* OR goal* OR target* OR requirement* OR framework* OR model * OR approach* OR practice* OR “health risk assessment” OR “impact assessment” OR “environmental assessment” OR “assessment tool” OR “assessment method” OR hia OR eia OR “sustainability appraisal” OR mitigation* OR “hierarchical system” OR “response hierarchy” OR compensation* OR offset* OR “off set” OR tradeoff* OR “trade off” OR reparation* OR restitution*)	2	3
	#31 from_date:2023 to_date:2024	N/A	1
#32	(“wellbeing net gain” or “wellbeing net benefit” or “wellbeing net improvement” or “wellbeing net increase” or “wellbeing net positive”)	0	0
#33	(“wellbeing net gain” or “wellbeing net benefit” or “wellbeing net improvement” or “wellbeing net increase” or “wellbeing net positive”) and (theor* OR concept* OR principle* OR policy OR policies OR plan OR plans OR planning OR strategy OR strategies OR objective* OR aim* OR goal* OR target* OR requirement* OR framework* OR model * OR approach* OR practice* OR “health risk assessment” OR “impact assessment” OR “environmental assessment” OR “assessment tool” OR “assessment method” OR hia OR eia OR “sustainability appraisal” OR mitigation* OR “hierarchical system” OR “response hierarchy” OR compensation* OR offset* OR “off set” OR tradeoff* OR “trade off” OR reparation* OR restitution*)	0	0
#34	(“well being net gain” or “well being net benefit” or “well being net improvement” or “well being net increase” or “well being net positive”)	0	0
#35	(“well being net gain” or “well being net benefit” or “well being net improvement” or “well being net increase” or “well being net positive”) and (theor* OR concept* OR principle* OR policy OR policies OR plan OR plans OR planning OR strategy OR strategies OR objective* OR aim* OR goal* OR target* OR requirement* OR framework* OR model * OR approach* OR practice* OR “health risk assessment” OR “impact assessment” OR “environmental assessment” OR “assessment tool” OR “assessment method” OR hia OR eia OR “sustainability appraisal” OR mitigation* OR “hierarchical system” OR “response hierarchy” OR compensation* OR offset* OR “off set” OR tradeoff* OR “trade off” OR reparation* OR restitution*)	0	0
#36	(“welfare net gain” or “welfare net benefit” or “welfare net improvement” or “welfare net increase” or “welfare net positive”)	0	0
#37	(“welfare net gain” or “welfare net benefit” or “welfare net improvement” or “welfare net increase” or “welfare net positive”) and (theor* OR concept* OR principle* OR policy OR policies OR plan OR plans OR planning OR strategy OR strategies OR objective* OR aim* OR goal* OR target* OR requirement* OR framework* OR model * OR approach* OR practice* OR “health risk assessment” OR “impact assessment” OR “environmental assessment” OR “assessment tool” OR “assessment method” OR hia OR eia OR “sustainability appraisal” OR mitigation* OR “hierarchical system” OR “response hierarchy” OR compensation* OR offset* OR “off set” OR tradeoff* OR “trade off” OR reparation* OR restitution*)	0	0
#38	(“social net gain” or “social net benefit” or “social net improvement” or “social net increase” or “social net positive”)	3	3
#39	(“social net gain” or “social net benefit” or “social net improvement” or “social net increase” or “social net positive”) and (theor* OR concept* OR principle* OR policy OR policies OR plan OR plans OR planning OR strategy OR strategies OR objective* OR aim* OR goal* OR target* OR requirement* OR framework* OR model * OR approach* OR practice* OR “health risk assessment” OR “impact assessment” OR “environmental assessment” OR “assessment tool” OR “assessment method” OR hia OR eia OR “sustainability appraisal” OR mitigation* OR “hierarchical system” OR “response hierarchy” OR compensation* OR offset* OR “off set” OR tradeoff* OR “trade off” OR reparation* OR restitution*)	2	2
	#39 from_date:2023 to_date:2024	N/A	0
#40	(“societal net gain” or “societal net benefit” or “societal net improvement” or “societal net increase” or “societal net positive”)	6	6
#41	(“societal net gain” or “societal net benefit” or “societal net improvement” or “societal net increase” or “societal net positive”) and (theor* OR concept* OR principle* OR policy OR policies OR plan OR plans OR planning OR strategy OR strategies OR objective* OR aim* OR goal* OR target* OR requirement* OR framework* OR model * OR approach* OR practice* OR “health risk assessment” OR “impact assessment” OR “environmental assessment” OR “assessment tool” OR “assessment method” OR hia OR eia OR “sustainability appraisal” OR mitigation* OR “hierarchical system” OR “response hierarchy” OR compensation* OR offset* OR “off set” OR tradeoff* OR “trade off” OR reparation* OR restitution*)	4	4
	#41 from_date:2023 to_date:2024	N/A	0
#42	(“society net gain” or “society net benefit” or “society net improvement” or “society net increase” or “society net positive”)	6	6
#43	(“society net gain” or “society net benefit” or “society net improvement” or “society net increase” or “society net positive”) and (theor* OR concept* OR principle* OR policy OR policies OR plan OR plans OR planning OR strategy OR strategies OR objective* OR aim* OR goal* OR target* OR requirement* OR framework* OR model * OR approach* OR practice* OR “health risk assessment” OR “impact assessment” OR “environmental assessment” OR “assessment tool” OR “assessment method” OR hia OR eia OR “sustainability appraisal” OR mitigation* OR “hierarchical system” OR “response hierarchy” OR compensation* OR offset* OR “off set” OR tradeoff* OR “trade off” OR reparation* OR restitution*)	4	4
	#43 from_date:2023 to_date:2024	N/A	0
#44	(“societies net gain” or “societies net benefit” or “societies net improvement” or “societies net increase” or “societies net positive”)	6	6
#45	(“societies net gain” or “societies net benefit” or “societies net improvement” or “societies net increase” or “societies net positive”) and (theor* OR concept* OR principle* OR policy OR policies OR plan OR plans OR planning OR strategy OR strategies OR objective* OR aim* OR goal* OR target* OR requirement* OR framework* OR model * OR approach* OR practice* OR “health risk assessment” OR “impact assessment” OR “environmental assessment” OR “assessment tool” OR “assessment method” OR hia OR eia OR “sustainability appraisal” OR mitigation* OR “hierarchical system” OR “response hierarchy” OR compensation* OR offset* OR “off set” OR tradeoff* OR “trade off” OR reparation* OR restitution*)	4	4
	#45 from_date:2023 to_date:2024	N/A	0
#46	(“sociodemographic net gain” or “sociodemographic net benefit” or “sociodemographic net improvement” or “sociodemographic net increase” or “sociodemographic net positive”)	0	0
#47	(“sociodemographic net gain” or “sociodemographic net benefit” or “sociodemographic net improvement” or “sociodemographic net increase” or “sociodemographic net positive”) and (theor* OR concept* OR principle* OR policy OR policies OR plan OR plans OR planning OR strategy OR strategies OR objective* OR aim* OR goal* OR target* OR requirement* OR framework* OR model * OR approach* OR practice* OR “health risk assessment” OR “impact assessment” OR “environmental assessment” OR “assessment tool” OR “assessment method” OR hia OR eia OR “sustainability appraisal” OR mitigation* OR “hierarchical system” OR “response hierarchy” OR compensation* OR offset* OR “off set” OR tradeoff* OR “trade off” OR reparation* OR restitution*)	0	0
#48	(“socioeconomic net gain” or “socioeconomic net benefit” or “socioeconomic net improvement” or “socioeconomic net increase” or “socioeconomic net positive”)	1	1
#49	(“socioeconomic net gain” or “socioeconomic net benefit” or “socioeconomic net improvement” or “socioeconomic net increase” or “socioeconomic net positive”) and (theor* OR concept* OR principle* OR policy OR policies OR plan OR plans OR planning OR strategy OR strategies OR objective* OR aim* OR goal* OR target* OR requirement* OR framework* OR model * OR approach* OR practice* OR “health risk assessment” OR “impact assessment” OR “environmental assessment” OR “assessment tool” OR “assessment method” OR hia OR eia OR “sustainability appraisal” OR mitigation* OR “hierarchical system” OR “response hierarchy” OR compensation* OR offset* OR “off set” OR tradeoff* OR “trade off” OR reparation* OR restitution*)	1	1
	#49 from_date:2023 to_date:2024	N/A	0
#50	(“sociological net gain” or “sociological net benefit” or “sociological net improvement” or “sociological net increase” or “sociological net positive”)	0	0
#51	(“sociological net gain” or “sociological net benefit” or “sociological net improvement” or “sociological net increase” or “sociological net positive”) and (theor* OR concept* OR principle* OR policy OR policies OR plan OR plans OR planning OR strategy OR strategies OR objective* OR aim* OR goal* OR target* OR requirement* OR framework* OR model * OR approach* OR practice* OR “health risk assessment” OR “impact assessment” OR “environmental assessment” OR “assessment tool” OR “assessment method” OR hia OR eia OR “sustainability appraisal” OR mitigation* OR “hierarchical system” OR “response hierarchy” OR compensation* OR offset* OR “off set” OR tradeoff* OR “trade off” OR reparation* OR restitution*)	0	0
#52	(“community net gain” or “community net benefit” or “community net improvement” or “community net increase” or “community net positive”)	0	0
#53	(“community net gain” or “community net benefit” or “community net improvement” or “community net increase” or “community net positive”) and (theor* OR concept* OR principle* OR policy OR policies OR plan OR plans OR planning OR strategy OR strategies OR objective* OR aim* OR goal* OR target* OR requirement* OR framework* OR model * OR approach* OR practice* OR “health risk assessment” OR “impact assessment” OR “environmental assessment” OR “assessment tool” OR “assessment method” OR hia OR eia OR “sustainability appraisal” OR mitigation* OR “hierarchical system” OR “response hierarchy” OR compensation* OR offset* OR “off set” OR tradeoff* OR “trade off” OR reparation* OR restitution*)	0	0
#54	Amended to bring in reparation/restitution		311
#55	(“net health gain” or “net health benefit” or “net health improvement” or “net health increase” or “net health positive”)	281	283
	#55 from_date:2023 to_date:2024	N/A	45
#56	(“net health gain” or “net health benefit” or “net health improvement” or “net health increase” or “net health positive”) and (theor* OR concept* OR principle* OR policy OR policies OR plan OR plans OR planning OR strategy OR strategies OR objective* OR aim* OR goal* OR target* OR requirement* OR framework* OR model * OR approach* OR practice* OR “health risk assessment” OR “impact assessment” OR “environmental assessment” OR “assessment tool” OR “assessment method” OR hia OR eia OR “sustainability appraisal” OR mitigation* OR “hierarchical system” OR “response hierarchy” OR compensation* OR offset* OR “off set” OR tradeoff* OR “trade off” OR reparation* OR restitution*)	254	0
#57	(“net wellbeing gain” or “net wellbeing benefit” or “net wellbeing improvement” or “net wellbeing increase” or “net wellbeing positive”)	0	0
#58	(“net wellbeing gain” or “net wellbeing benefit” or “net wellbeing improvement” or “net wellbeing increase” or “net wellbeing positive”) and (theor* OR concept* OR principle* OR policy OR policies OR plan OR plans OR planning OR strategy OR strategies OR objective* OR aim* OR goal* OR target* OR requirement* OR framework* OR model * OR approach* OR practice* OR “health risk assessment” OR “impact assessment” OR “environmental assessment” OR “assessment tool” OR “assessment method” OR hia OR eia OR “sustainability appraisal” OR mitigation* OR “hierarchical system” OR “response hierarchy” OR compensation* OR offset* OR “off set” OR tradeoff* OR “trade off” OR reparation* OR restitution*)	0	0
#59	(“net well being gain” or “net well being benefit” or “net well being improvement” or “net well being increase” or “net well being positive”)	0	0
#60	(“net well being gain” or “net well being benefit” or “net well being improvement” or “net well being increase” or “net well being positive”) and (theor* OR concept* OR principle* OR policy OR policies OR plan OR plans OR planning OR strategy OR strategies OR objective* OR aim* OR goal* OR target* OR requirement* OR framework* OR model * OR approach* OR practice* OR “health risk assessment” OR “impact assessment” OR “environmental assessment” OR “assessment tool” OR “assessment method” OR hia OR eia OR “sustainability appraisal” OR mitigation* OR “hierarchical system” OR “response hierarchy” OR compensation* OR offset* OR “off set” OR tradeoff* OR “trade off” OR reparation* OR restitution*)	0	2
#61	(“net welfare gain” or “net welfare benefit” or “net welfare improvement” or “net welfare increase” or “net welfare positive”)	2	2
	#61 from_date:2023 to_date:2024	N/A	0
#62	(“net welfare gain” or “net welfare benefit” or “net welfare improvement” or “net welfare increase” or “net welfare positive”) and (theor* OR concept* OR principle* OR policy OR policies OR plan OR plans OR planning OR strategy OR strategies OR objective* OR aim* OR goal* OR target* OR requirement* OR framework* OR model * OR approach* OR practice* OR “health risk assessment” OR “impact assessment” OR “environmental assessment” OR “assessment tool” OR “assessment method” OR hia OR eia OR “sustainability appraisal” OR mitigation* OR “hierarchical system” OR “response hierarchy” OR compensation* OR offset* OR “off set” OR tradeoff* OR “trade off” OR reparation* OR restitution*)	2	21
#63	(“net social gain” or “net social benefit” or “net social improvement” or “net social increase” or “net social positive”)	21	19
	#63 from_date:2023 to_date:2024	N/A	1
#64	(“net social gain” or “net social benefit” or “net social improvement” or “net social increase” or “net social positive”) and (theor* OR concept* OR principle* OR policy OR policies OR plan OR plans OR planning OR strategy OR strategies OR objective* OR aim* OR goal* OR target* OR requirement* OR framework* OR model * OR approach* OR practice* OR “health risk assessment” OR “impact assessment” OR “environmental assessment” OR “assessment tool” OR “assessment method” OR hia OR eia OR “sustainability appraisal” OR mitigation* OR “hierarchical system” OR “response hierarchy” OR compensation* OR offset* OR “off set” OR tradeoff* OR “trade off” OR reparation* OR restitution*)	19	24
#65	(“net societal gain” or “net societal benefit” or “net societal improvement” or “net societal increase” or “net societal positive”)	24	17
	#65 from_date:2023 to_date:2024	N/A	0
#66	(“net societal gain” or “net societal benefit” or “net societal improvement” or “net societal increase” or “net societal positive”) and (theor* OR concept* OR principle* OR policy OR policies OR plan OR plans OR planning OR strategy OR strategies OR objective* OR aim* OR goal* OR target* OR requirement* OR framework* OR model * OR approach* OR practice* OR “health risk assessment” OR “impact assessment” OR “environmental assessment” OR “assessment tool” OR “assessment method” OR hia OR eia OR “sustainability appraisal” OR mitigation* OR “hierarchical system” OR “response hierarchy” OR compensation* OR offset* OR “off set” OR tradeoff* OR “trade off” OR reparation* OR restitution*)	17	24
#67	(“net society gain” or “net society benefit” or “net society improvement” or “net society increase” or “net society positive”)	24	4
	#67 from_date:2023 to_date:2024	N/A	0
#68	(“society net gain” or “society net benefit” or “society net improvement” or “society net increase” or “society net positive”) and (theor* OR concept* OR principle* OR policy OR policies OR plan OR plans OR planning OR strategy OR strategies OR objective* OR aim* OR goal* OR target* OR requirement* OR framework* OR model * OR approach* OR practice* OR “health risk assessment” OR “impact assessment” OR “environmental assessment” OR “assessment tool” OR “assessment method” OR hia OR eia OR “sustainability appraisal” OR mitigation* OR “hierarchical system” OR “response hierarchy” OR compensation* OR offset* OR “off set” OR tradeoff* OR “trade off” OR reparation* OR restitution*)	4	24
#69	(“net societies gain” or “net societies benefit” or “net societies improvement” or “net societies increase” or “net societies positive”)	24	4
	#69 from_date:2023 to_date:2024	N/A	0
#70	(“societies net gain” or “societies net benefit” or “societies net improvement” or “societies net increase” or “societies net positive”) and (theor* OR concept* OR principle* OR policy OR policies OR plan OR plans OR planning OR strategy OR strategies OR objective* OR aim* OR goal* OR target* OR requirement* OR framework* OR model * OR approach* OR practice* OR “health risk assessment” OR “impact assessment” OR “environmental assessment” OR “assessment tool” OR “assessment method” OR hia OR eia OR “sustainability appraisal” OR mitigation* OR “hierarchical system” OR “response hierarchy” OR compensation* OR offset* OR “off set” OR tradeoff* OR “trade off” OR reparation* OR restitution*)	4	0
#71	(“net sociodemographic gain” or “net sociodemographic benefit” or “net sociodemographic improvement” or “net sociodemographic increase” or “net sociodemographic positive”)	0	0
#72	(“sociodemographic net gain” or “sociodemographic net benefit” or “sociodemographic net improvement” or “sociodemographic net increase” or “sociodemographic net positive”) and (theor* OR concept* OR principle* OR policy OR policies OR plan OR plans OR planning OR strategy OR strategies OR objective* OR aim* OR goal* OR target* OR requirement* OR framework* OR model * OR approach* OR practice* OR “health risk assessment” OR “impact assessment” OR “environmental assessment” OR “assessment tool” OR “assessment method” OR hia OR eia OR “sustainability appraisal” OR mitigation* OR “hierarchical system” OR “response hierarchy” OR compensation* OR offset* OR “off set” OR tradeoff* OR “trade off” OR reparation* OR restitution*)	0	1
#73	(“net socioeconomic gain” or “net socioeconomic benefit” or “net socioeconomic improvement” or “net socioeconomic increase” or “net socioeconomic positive”)	1	1
	#73 from_date:2023 to_date:2024	N/A	0
#74	(“socioeconomic net gain” or “socioeconomic net benefit” or “socioeconomic net improvement” or “socioeconomic net increase” or “socioeconomic net positive”) and (theor* OR concept* OR principle* OR policy OR policies OR plan OR plans OR planning OR strategy OR strategies OR objective* OR aim* OR goal* OR target* OR requirement* OR framework* OR model * OR approach* OR practice* OR “health risk assessment” OR “impact assessment” OR “environmental assessment” OR “assessment tool” OR “assessment method” OR hia OR eia OR “sustainability appraisal” OR mitigation* OR “hierarchical system” OR “response hierarchy” OR compensation* OR offset* OR “off set” OR tradeoff* OR “trade off” OR reparation* OR restitution*)	1	0
#75	(“net sociological gain” or “net sociological benefit” or “net sociological improvement” or “net sociological increase” or “net sociological positive”)	0	0
#76	(“sociological net gain” or “sociological net benefit” or “sociological net improvement” or “sociological net increase” or “sociological net positive”) and (theor* OR concept* OR principle* OR policy OR policies OR plan OR plans OR planning OR strategy OR strategies OR objective* OR aim* OR goal* OR target* OR requirement* OR framework* OR model * OR approach* OR practice* OR “health risk assessment” OR “impact assessment” OR “environmental assessment” OR “assessment tool” OR “assessment method” OR hia OR eia OR “sustainability appraisal” OR mitigation* OR “hierarchical system” OR “response hierarchy” OR compensation* OR offset* OR “off set” OR tradeoff* OR “trade off” OR reparation* OR restitution*)	0	0
#77	(“net community gain” or “net community benefit” or “net community improvement” or “net community increase” or “net community positive”)	0	0
	**Total records retrieved (sum)**	**1448**	**1576**
	**Total records retrieved (sum) from_date:2023 to_date:2024**	**N/A**	**138**

**Table:**

IBSS (ProQuest)			
**Searches conducted: August 17, 2023; July 18, 2024**			
**Search**	**Query**	**Records retrieved (17/08/23)**	**Records retrieved (18/07/24)**
#1	TITLE,ABSTRACT,SUBJECT(“net outcome*” or “net gain*” or “net positive outcome*” or “net positive impact*” or “no net loss*” or “mitigation hierarch*” or “regenerative design*” or “regenerative development*” or “regenerative sustainability”)	515	509
#2	SUBJECT(“net positive*” or “net benefit*” or “positive development*”)	15	8
#3	[S1] or [S2]	529	517
#4	(MAINSUBJECT.EXACT.EXPLODE(“Theory” OR “Public policy” OR “Strategies” OR “Planning”) OR MAINSUBJECT.EXACT(“Conceptual models” OR “Concepts” OR “Concept formation” OR “Principles” OR “Standards” OR “Policy making” OR “Policy implementation” OR “Plans” OR “Professional practices” OR “Practice” OR “Approaches”))	385,056	413,817
#5	SUBJECT(theor* or concept* or principle* or policy or policies or plan or plans or planning or strategy or strategies or objective* or aim* or goal* or target* or requirement* or framework* or model* or approach* or practice*)	1,407,370	1,464,605
#6	MAINSUBJECT.EXACT(“Strategic impact assessment” OR “Health impact assessment” OR “Environmental impact assessment” OR “Strategic environmental assessment”)	436	477
#7	TITLE,ABSTRACT,SUBJECT(“health risk assessment*” OR “impact assessment*” OR “environmental assessment*” OR “assessment tool*” OR “assessment method*”)	10,682	11,151
#8	TITLE,ABSTRACT,SUBJECT(HIA OR HIAs OR EIA OR EIAs OR “sustainability appraisal*”)	1404	1425
#9	(MAINSUBJECT.EXACT.EXPLODE(“Compensation”) OR MAINSUBJECT.EXACT(“Mitigation” OR “Reparations”))	17,734	19,310
#10	TITLE,ABSTRACT,SUBJECT(mitigation* OR “hierarchical system*” OR “response hierarch*” OR compensation* OR offset* OR “off-set*” OR tradeoff* OR “trade-off*” OR reparation* OR restitution*)	69,129	71,184
#11	[S4] or [S5] or [S6] or [S7] or [S8] or [S9] or [S10]	1,457,583	1,516,365
#12	[S3] and [S11]	311	304
#13	TITLE,ABSTRACT,SUBJECT(health* OR wellbeing OR “well-being” OR welfare)	420,497	437,382
#14	(MAINSUBJECT.EXACT.EXPLODE(“Well being”) OR MAINSUBJECT.EXACT(“Health” OR “Public health” OR “Health status” OR “Health disparities” OR “Quality of life” OR “Welfare” OR “Social welfare”))	152,665	159,649
#15	(MAINSUBJECT.EXACT.EXPLODE(“Social justice” OR “Social processes”) OR MAINSUBJECT.EXACT(“Social factors” OR “Social conditions” OR “Social issues” OR “Social problems” OR “Distributive justice” OR “Restorative justice” OR “Social environment” OR “Social impact” OR “Social inequality” OR “Sociological aspects” OR “Socioeconomic factors” OR “Sociodemographics” OR “Society”))	256,081	268,947
#16	SUBJECT(social* OR societal* OR society OR societies OR sociodemographic* OR socioeconomic* OR sociological*)	1,055,481	1,101,429
#17	(MAINSUBJECT.EXACT.EXPLODE(“Population”) OR MAINSUBJECT.EXACT(“Humans” OR “Residents” OR “Local population” OR “Stakeholders” OR “Interest groups” OR “Community” OR “Local communities” OR “Master planned communities”))	131,599	137,304
#18	SUBJECT(human* OR population* OR stakeholder* OR community or communities)	456,362	490,157
#19	[S13] or [S14] or [S15] or [S16] or [S17] or [S18]	1,649,620	1,721,291
#20	[S3] and [S19]	198	192
#21	TITLE,ABSTRACT,SUBJECT((health* OR wellbeing OR “well-being” OR welfare OR soci* OR commun*) NEAR/2 net NEAR/2 (gain? OR benefit? OR improve* OR increase? OR positive?))	437	442
#22	[S21] and [S11]	279	288
	([S21] and [S11]) AND pd(20230101-20240718)	N/A	9
#23	TITLE,ABSTRACT,SUBJECT(“health net gain*” OR “net health gain*” OR “wellbeing net gain*” OR “net wellbeing gain*” OR “well-being net gain*” OR “net well-being gain*”)	2	2
#24	[S12] or [S20] or [S23]	384	376
	([S12] or [S20] or [S23]) AND pd(20230101-20240718)	N/A	9
#25	TITLE,ABSTRACT,SUBJECT((“net outcome?” OR “net gain?” OR “net benefit?” OR “net improve*” OR “net increase?” OR “net positive?”) PRE/0 (theor* OR concept* OR principle? OR polic* OR plan* OR strateg* OR objective? OR aim? OR goal? OR target? OR requirement? OR framework? OR model? OR assessment? OR appraisal? OR evaluation? OR approach? OR practice? OR tool?))	13	13
	(TITLE,ABSTRACT,SUBJECT((“net outcome?” OR “net gain?” OR “net benefit?” OR “net improve*” OR “net increase?” OR “net positive?”) PRE/0 (theor* OR concept* OR principle? OR polic* OR plan* OR strateg* OR objective? OR aim? OR goal? OR target? OR requirement? OR framework? OR model? OR assessment? OR appraisal? OR evaluation? OR approach? OR practice? OR tool?))) AND pd(20230101-20240718)	N/A	0
#26	TITLE,ABSTRACT,SUBJECT((“positive outcome?” OR “net positive outcome?” OR “positive impact?” OR “net positive impact?” OR “positive development?”) PRE/0 (theor* OR concept* OR principle? OR polic* OR plan* OR strateg* OR objective? OR aim? OR goal? OR target? OR requirement? OR framework? OR model? OR assessment? OR appraisal? OR evaluation? OR approach? OR practice? OR tool?))	12	12
	(TITLE,ABSTRACT,SUBJECT((“positive outcome?” OR “net positive outcome?” OR “positive impact?” OR “net positive impact?” OR “positive development?”) PRE/0 (theor* OR concept* OR principle? OR polic* OR plan* OR strateg* OR objective? OR aim? OR goal? OR target? OR requirement? OR framework? OR model? OR assessment? OR appraisal? OR evaluation? OR approach? OR practice? OR tool?))) AND pd(20230101-20240718)	N/A	2
#27	TITLE,ABSTRACT,SUBJECT((“regenerative design?” OR “regenerative development?” OR “regenerative sustainability” OR “no net loss*” OR “mitigation hierarch*”) PRE/0 (theor* OR concept* OR principle? OR polic* OR plan* OR strateg* OR objective? OR aim? OR goal? OR target? OR requirement? OR framework? OR model? OR assessment? OR appraisal? OR evaluation? OR approach? OR practice? OR tool?))	11	11
	(TITLE,ABSTRACT,SUBJECT((“regenerative design?” OR “regenerative development?” OR “regenerative sustainability” OR “no net loss*” OR “mitigation hierarch*”) PRE/0 (theor* OR concept* OR principle? OR polic* OR plan* OR strateg* OR objective? OR aim? OR goal? OR target? OR requirement? OR framework? OR model? OR assessment? OR appraisal? OR evaluation? OR approach? OR practice? OR tool?))) AND pd(20230101-20240718)	N/A	1
	**Total records retrieved (sum #22, #24-27)**	**699**	**700**
	**Total records retrieved (sum #22, #24-27 for date range 20230101-20240718)**	**N/A**	**21**

**Table:**

Dissertations and Theses A&I (ProQuest)			
**Searches conducted: August 17, 2023; July 12, 2024**			
**Search**	**Query**	**Records retrieved (17/08/23)**	**Records retrieved (12/07/24)**
#1	TITLE,ABSTRACT,SUBJECT(“net outcome*” or “net gain*” or “net positive outcome*” or “net positive impact*” or “no net loss*” or “mitigation hierarch*” or “regenerative design*” or “regenerative development*” or “regenerative sustainability”)	614	655
#2	SUBJECT(“net positive*” or “net benefit*” or “positive development*”)	43	48
#3	[S1] or [S2]	657	703
#4	MAINSUBJECT.EXACT(“Standards” OR “Guidelines”)	922	1241
#5	SUBJECT(theor* or concept* or principle* or policy or policies or plan or plans or planning or strategy or strategies or objective* or aim* or goal* or target* or requirement* or framework* or model* or approach* or practice*)	344,322	412,917
#6	MAINSUBJECT.EXACT(“Environmental impact statements”)	12	15
#7	TITLE,ABSTRACT,SUBJECT(“health risk assessment*” OR “impact assessment*” OR “environmental assessment*” OR “assessment tool*” OR “assessment method*”)	17,038	18,753
#8	TITLE,ABSTRACT,SUBJECT(HIA OR HIAs OR EIA OR EIAs OR “sustainability appraisal*”)	1245	1352
#9	TITLE,ABSTRACT,SUBJECT(mitigation* OR “hierarchical system*” OR “response hierarch*” OR compensation* OR offset* OR “off-set*” OR tradeoff* OR “trade-off*” OR reparation* OR restitution*)	93,367	99,841
#10	[S4] or [S5] or [S6] or [S7] or [S8] or [S9]	440,717	515,668
#11	[S3] and [S10]	170	184
#12	TITLE,ABSTRACT,SUBJECT(health* OR wellbeing OR “well-being” OR welfare)	796,355	857,968
#13	MAINSUBJECT.EXACT(“Quality of life”)	3679	5112
#14	MAINSUBJECT.EXACT(“Distributive justice” OR “Restorative justice”)	259	359
#15	SUBJECT(social* OR societal* OR society OR societies OR sociodemographic* OR socioeconomic* OR sociological*)	1,566,558	1,592,180
#16	MAINSUBJECT.EXACT(“Residents” OR “Interest groups”)	543	617
#17	SUBJECT(human* OR population* OR stakeholder* OR community or communities)	124,338	141,231
#18	[S12] or [S13] or [S14] or [S15] or [S16] or [S17]	2,222,130	2,311,061
#19	[S3] and [S18]	315	335
#20	TITLE,ABSTRACT,SUBJECT((health* OR wellbeing OR “well-being” OR welfare OR soci* OR commun*) NEAR/2 net NEAR/2 (gain? OR benefit? OR improve* OR increase? OR positive?))	291	304
#21	[S20] and [S10]	115	122
	([S20] and [S10]) AND pd(20230101-20240712)	N/A	6
#22	[S11] or [S19]	373	398
	([S11] or [S19]) AND pd(20230101-20240712)	N/A	18
#23	TITLE,ABSTRACT,SUBJECT((“net outcome?” OR “net gain?” OR “net benefit?” OR “net improve*” OR “net increase?” OR “net positive?”) PRE/0 (theor* OR concept* OR principle? OR polic* OR plan* OR strateg* OR objective? OR aim? OR goal? OR target? OR requirement? OR framework? OR model? OR assessment? OR appraisal? OR evaluation? OR approach? OR practice? OR tool?))	17	18
	TITLE,ABSTRACT,SUBJECT((“net outcome?” OR “net gain?” OR “net benefit?” OR “net improve*” OR “net increase?” OR “net positive?”) PRE/0 (theor* OR concept* OR principle? OR polic* OR plan* OR strateg* OR objective? OR aim? OR goal? OR target? OR requirement? OR framework? OR model? OR assessment? OR appraisal? OR evaluation? OR approach? OR practice? OR tool?)) AND pd(20230101-20240712)	N/A	0
#24	TITLE,ABSTRACT,SUBJECT((“positive outcome?” OR “net positive outcome?” OR “positive impact?” OR “net positive impact?” OR “positive development?”) PRE/0 (theor* OR concept* OR principle? OR polic* OR plan* OR strateg* OR objective? OR aim? OR goal? OR target? OR requirement? OR framework? OR model? OR assessment? OR appraisal? OR evaluation? OR approach? OR practice? OR tool?))	9	11
	TITLE,ABSTRACT,SUBJECT((“positive outcome?” OR “net positive outcome?” OR “positive impact?” OR “net positive impact?” OR “positive development?”) PRE/0 (theor* OR concept* OR principle? OR polic* OR plan* OR strateg* OR objective? OR aim? OR goal? OR target? OR requirement? OR framework? OR model? OR assessment? OR appraisal? OR evaluation? OR approach? OR practice? OR tool?)) AND pd(20230101-20240712)	N/A	1
#25	TITLE,ABSTRACT,SUBJECT((“regenerative design?” OR “regenerative development?” OR “regenerative sustainability” OR “no net loss*” OR “mitigation hierarch*”) PRE/0 (theor* OR concept* OR principle? OR polic* OR plan* OR strateg* OR objective? OR aim? OR goal? OR target? OR requirement? OR framework? OR model? OR assessment? OR appraisal? OR evaluation? OR approach? OR practice? OR tool?))	29	33
	TITLE,ABSTRACT,SUBJECT((“regenerative design?” OR “regenerative development?” OR “regenerative sustainability” OR “no net loss*” OR “mitigation hierarch*”) PRE/0 (theor* OR concept* OR principle? OR polic* OR plan* OR strateg* OR objective? OR aim? OR goal? OR target? OR requirement? OR framework? OR model? OR assessment? OR appraisal? OR evaluation? OR approach? OR practice? OR tool?)) AND pd(20230101-20240712)	N/A	3
	**Total records retrieved (sum #21, #22-25)**	**543**	**582**
	**Total records retrieved (sum #21, #22-25 for date range 20230101-20240712)**	**N/A**	**28**

**Table:**

APA PsycINFO (Ovid)			
**Searches conducted: August 17, 2023; July 12, 2024**			
**Search**	**Query**	**Records retrieved (17/08/23)**	**Records retrieved (12/07/24)**
#1	(“net outcome*” or “net gain*” or “net positive outcome*” or “net positive impact*” or “no net loss*” or “mitigation hierarch*” or “regenerative design*” or “regenerative development*” or “regenerative sustainability”).mp.	234	244
#2	(“net positive*” or “net benefit*” or “positive development*”).hw,id,mf,mh.	284	298
#3	1 or 2	518	542
#4	exp theories/ or exp concepts/ or exp policy making/ or strategies/ or exp environmental planning/ or practice/	526,623	544,130
#5	(theor* or concept* or principle* or policy or policies or plan or plans or planning or strategy or strategies or objective* or aim* or goal* or target* or requirement* or framework* or model* or approach* or practice*).hw,id,mf,mh.	1,166,457	1,203,975
#6	(“health risk assessment*” or “impact assessment*” or “environmental assessment*” or “assessment tool*” or “assessment method*”).mp.	23,992	25,651
#7	(HIA or HIAs or EIA or EIAs or “sustainability appraisal*”).mp.	327	338
#8	(mitigation* or “hierarchical system*” or “response hierarch*” or compensation* or offset* or “off-set*” or tradeoff* or “trade-off*” or reparation* or restitution*).mp.	44,908	47,383
#9	4 or 5 or 6 or 7 or 8	1,288,905	1,331,924
#10	((“net outcome*” or “net gain*” or “net benefit*” or “net improve*” or “net increase*” or “net positive*” or “positive outcome*” or “net positive outcome*” or “positive impact*” or “net positive impact*” or “positive development*” or “regenerative design*” or “regenerative development*” or “regenerative sustainability” or “no net loss*” or “mitigation hierarch*”) adj (theor* or concept* or principle* or polic* or plan* or strateg* or objective* or aim* or goal* or target* or requirement* or framework* or model* or assessment* or appraisal* or evaluation* or approach* or practice* or tool*)).mp.	72	77
#11	(3 and 9) or 10	224	234
#12	(health* or wellbeing or “well-being” or welfare).mp.	1,173,400	1,243,613
#13	exp health/	427,408	491,253
#14	exp social issues/ or exp social environments/ or exp socioeconomic factors/ or exp social processes/ or society/	850,062	893,256
#15	(social* or societal* or society or societies or sociodemographic* or socioeconomic* or sociological*).hw,id,mf,mh.	777,343	806,303
#16	exp population/ or stakeholder/	221,152	231,110
#17	(human* or population* or stakeholder* or community or communities).hw,id,mf,mh.	1,795,709	1,838,284
#18	12 or 13 or 14 or 15 or 16 or 17	3,050,881	3,174,550
#19	3 and 18	329	347
#20	((health* or wellbeing or “well-being” or welfare or soci* or commun*) adj3 net adj3 (gain* or benefit* or improve* or increase* or positive*)).mp.	123	130
#21	9 and 20	58	59
#22	(“health net gain*” or “net health gain*” or “wellbeing net gain*” or “net wellbeing gain*” or “well-being net gain*” or “net well-being gain*”).mp.	3	3
#23	11 or 19 or 21 or 22	505	530
#24	limit 23 to up=20230801-20240712	N/A	25

**Table:**

PAIS Index (ProQuest)			
**Searches conducted: August 17, 2023; July 12, 2024**			
**Search**	**Query**	**Records retrieved (17/08/23)**	**Records retrieved (12/07/24)**
#1	TITLE,ABSTRACT,SUBJECT(“net outcome*” or “net gain*” or “net positive outcome*” or “net positive impact*” or “no net loss*” or “mitigation hierarch*” or “regenerative design*” or “regenerative development*” or “regenerative sustainability”)	178	193
#2	SUBJECT(“net positive*” or “net benefit*” or “positive development*”)	2	1
#3	[S1] or [S2]	180	194
#4	(MAINSUBJECT.EXACT.EXPLODE(“Theories” OR “Theory formation” OR “Public policy” OR “Policy making” OR “Policy implementation” OR “Strategies” OR “Planning”) OR MAINSUBJECT.EXACT(“Concepts” OR “Concept formation” OR “Principles” OR “Standards” OR “Methodological approaches”))	109,709	147,438
#5	SUBJECT(theor* or concept* or principle* or policy or policies or plan or plans or planning or strategy or strategies or objective* or aim* or goal* or target* or requirement* or framework* or model* or approach* or practice*)	536,161	605,177
#6	MAINSUBJECT.EXACT(“Environmental impact assessment” OR “Social impact assessment”)	122	283
#7	TITLE,ABSTRACT,SUBJECT(“health risk assessment*” OR “impact assessment*” OR “environmental assessment*” OR “assessment tool*” OR “assessment method*”)	4479	5428
#8	TITLE,ABSTRACT,SUBJECT(HIA OR HIAs OR EIA OR EIAs OR “sustainability appraisal*”)	413	553
#9	(MAINSUBJECT.EXACT.EXPLODE(“Compensation”) OR MAINSUBJECT.EXACT(“Restitution”))	15,635	17,488
#10	TITLE,ABSTRACT,SUBJECT(mitigation* OR “hierarchical system*” OR “response hierarch*” OR compensation* OR offset* OR “off-set*” OR tradeoff* OR “trade-off*” OR reparation* OR restitution*)	32,921	36,410
#11	[S4] or [S5] or [S6] or [S7] or [S8] or [S9] or [S10]	563,296	633,609
#12	[S3] and [S11]	125	136
#13	TITLE,ABSTRACT,SUBJECT(health* OR wellbeing OR “well-being” OR welfare)	206,712	232,978
#14	(MAINSUBJECT.EXACT.EXPLODE(“Health” OR “Quality of life” OR “Social welfare”) OR MAINSUBJECT.EXACT(“Health planning” OR “Health disparities” OR “Welfare”))	62,611	83,581
#15	(MAINSUBJECT.EXACT.EXPLODE(“Social justice” OR “Social processes”) OR MAINSUBJECT.EXACT(“Social factors” OR “Social conditions” OR “Social problems” OR “Restorative justice” OR “Social environment” OR “Social impact” OR “Social inequality” OR “Socioeconomic factors” OR “Sociodemographics” OR “Society”))	39,555	51,572
#16	SUBJECT(social* OR societal* OR society OR societies OR sociodemographic* OR socioeconomic* OR sociological*)	262,679	287,469
#17	(MAINSUBJECT.EXACT.EXPLODE(“Population”) OR MAINSUBJECT.EXACT(“Residents” OR “Interest groups” OR “Community”))	33,619	40,809
#18	SUBJECT(human* OR population* OR stakeholder* OR community or communities)	186,739	207,708
#19	[S13] or [S14] or [S15] or [S16] or [S17] or [S18]	505,686	557,135
#20	[S3] and [S19]	83	92
#21	TITLE,ABSTRACT,SUBJECT((health* OR wellbeing OR “well-being” OR welfare OR soci* OR commun*) NEAR/2 net NEAR/2 (gain? OR benefit? OR improve* OR increase? OR positive?))	185	209
#22	[S21] and [S11]	149	171
	([S21] and [S11]) AND pd(20230101-20240712)	N/A	5
#23	TITLE,ABSTRACT,SUBJECT(“health net gain*” OR “net health gain*” OR “wellbeing net gain*” OR “net wellbeing gain*” OR “well-being net gain*” OR “net well-being gain*”)	2	3
#24	[S12] or [S20] or [S23]	149	161
	([S12] or [S20] or [S23]) AND pd(20230101-20240712)	N/A	1
#25	TITLE,ABSTRACT,SUBJECT((“net outcome?” OR “net gain?” OR “net benefit?” OR “net improve*” OR “net increase?” OR “net positive?”) PRE/0 (theor* OR concept* OR principle? OR polic* OR plan* OR strateg* OR objective? OR aim? OR goal? OR target? OR requirement? OR framework? OR model? OR assessment? OR appraisal? OR evaluation? OR approach? OR practice? OR tool?))	3	3
	TITLE,ABSTRACT,SUBJECT((“net outcome?” OR “net gain?” OR “net benefit?” OR “net improve*” OR “net increase?” OR “net positive?”) PRE/0 (theor* OR concept* OR principle? OR polic* OR plan* OR strateg* OR objective? OR aim? OR goal? OR target? OR requirement? OR framework? OR model? OR assessment? OR appraisal? OR evaluation? OR approach? OR practice? OR tool?)) AND pd(20230101-20240712)	N/A	0
#26	TITLE,ABSTRACT,SUBJECT((“positive outcome?” OR “net positive outcome?” OR “positive impact?” OR “net positive impact?” OR “positive development?”) PRE/0 (theor* OR concept* OR principle? OR polic* OR plan* OR strateg* OR objective? OR aim? OR goal? OR target? OR requirement? OR framework? OR model? OR assessment? OR appraisal? OR evaluation? OR approach? OR practice? OR tool?))	3	5
	TITLE,ABSTRACT,SUBJECT((“positive outcome?” OR “net positive outcome?” OR “positive impact?” OR “net positive impact?” OR “positive development?”) PRE/0 (theor* OR concept* OR principle? OR polic* OR plan* OR strateg* OR objective? OR aim? OR goal? OR target? OR requirement? OR framework? OR model? OR assessment? OR appraisal? OR evaluation? OR approach? OR practice? OR tool?)) AND pd(20230101-20240712)	N/A	1
#27	TITLE,ABSTRACT,SUBJECT((“regenerative design?” OR “regenerative development?” OR “regenerative sustainability” OR “no net loss*” OR “mitigation hierarch*”) PRE/0 (theor* OR concept* OR principle? OR polic* OR plan* OR strateg* OR objective? OR aim? OR goal? OR target? OR requirement? OR framework? OR model? OR assessment? OR appraisal? OR evaluation? OR approach? OR practice? OR tool?))	8	8
	TITLE,ABSTRACT,SUBJECT((“regenerative design?” OR “regenerative development?” OR “regenerative sustainability” OR “no net loss*” OR “mitigation hierarch*”) PRE/0 (theor* OR concept* OR principle? OR polic* OR plan* OR strateg* OR objective? OR aim? OR goal? OR target? OR requirement? OR framework? OR model? OR assessment? OR appraisal? OR evaluation? OR approach? OR practice? OR tool?)) AND pd(20230101-20240712)	N/A	0
	**Total records retrieved (sum #22, #24-27)**	**312**	**348**
	**Total records retrieved (sum #22, #24-27 for date range 20230101-20240712)**	**N/A**	**7**

**Table:**

Social Science Database (ProQuest)			
**Searches conducted: August 17, 2023; July 12, 2024**			
**Search**	**Query**	**Records retrieved (17/08/23)**	**Records retrieved (12/07/24)**
#1	TITLE,ABSTRACT,SUBJECT(“net outcome*” or “net gain*” or “net positive outcome*” or “net positive impact*” or “no net loss*” or “mitigation hierarch*” or “regenerative design*” or “regenerative development*” or “regenerative sustainability”)	225	228
#2	SUBJECT(“net positive*” or “net benefit*” or “positive development*”)	11	17
#3	[S1] or [S2]	236	245
#4	(MAINSUBJECT.EXACT(“Theory” OR “Principles” OR “Standards” OR “Public policy” OR “Policy making” OR “Planning” OR “Professional practice” OR “Guidelines”))	85,759	92,371
#5	SUBJECT(theor* or concept* or principle* or policy or policies or plan or plans or planning or strategy or strategies or objective* or aim* or goal* or target* or requirement* or framework* or model* or approach* or practice*)	498,648	553,512
#6	MAINSUBJECT.EXACT(“Health risk assessment” OR “Environmental impact statements”)	5917	6042
#7	TITLE,ABSTRACT,SUBJECT(“health risk assessment*” OR “impact assessment*” OR “environmental assessment*” OR “assessment tool*” OR “assessment method*”)	10,178	10,665
#8	TITLE,ABSTRACT,SUBJECT(HIA OR HIAs OR EIA OR EIAs OR “sustainability appraisal*”)	333	345
#9	(MAINSUBJECT.EXACT(“Compensation” OR “Reparations” OR “Restitution”))	5872	6373
#10	TITLE,ABSTRACT,SUBJECT(mitigation* OR “hierarchical system*” OR “response hierarch*” OR compensation* OR offset* OR “off-set*” OR tradeoff* OR “trade-off*” OR reparation* OR restitution*)	32,140	33,522
#11	[S4] or [S5] or [S6] or [S7] or [S8] or [S9] or [S10]	531,858	588,195
#12	[S3] and [S11]	110	115
#13	TITLE,ABSTRACT,SUBJECT(health* OR wellbeing OR “well-being” OR welfare)	390,098	415,658
#14	(MAINSUBJECT.EXACT(“Health” OR “Public health” OR “Health disparities” OR “Quality of life” OR “Welfare”))	90,832	103,863
#15	(MAINSUBJECT.EXACT(“Social conditions & trends” OR “Social justice” OR “Distributive justice” OR “Restorative justice” OR “Social impact” OR “Socioeconomic factors” OR “Sociodemographics” OR “Society”))	104,735	110,582
#16	SUBJECT(social* OR societal* OR society OR societies OR sociodemographic* OR socioeconomic* OR sociological*)	475,041	525,047
#17	(MAINSUBJECT.EXACT(“Population” OR “Residents” OR “Stakeholders” OR “Interest groups” OR “Community” OR “Master planned communities”))	61,413	66,045
#18	SUBJECT(human* OR population* OR stakeholder* OR community or communities)	301,137	336,196
#19	[S13] or [S14] or [S15] or [S16] or [S17] or [S18]	906,004	980,484
#20	[S3] and [S19]	101	108
#21	TITLE,ABSTRACT,SUBJECT((health* OR wellbeing OR “well-being” OR welfare OR soci* OR commun*) NEAR/2 net NEAR/2 (gain? OR benefit? OR improve* OR increase? OR positive?))	170	169
#22	[S21] and [S11]	102	102
	([S21] and [S11]) AND pd(20230101-20240712)	N/A	3
#23	[S12] or [S20]	152	163
	([S12] or [S20]) AND pd(20230101-20240712)	N/A	8
#24	TITLE,ABSTRACT,SUBJECT((“net outcome?” OR “net gain?” OR “net benefit?” OR “net improve*” OR “net increase?” OR “net positive?”) PRE/0 (theor* OR concept* OR principle? OR polic* OR plan* OR strateg* OR objective? OR aim? OR goal? OR target? OR requirement? OR framework? OR model? OR assessment? OR appraisal? OR evaluation? OR approach? OR practice? OR tool?))	6	6
	TITLE,ABSTRACT,SUBJECT((“net outcome?” OR “net gain?” OR “net benefit?” OR “net improve*” OR “net increase?” OR “net positive?”) PRE/0 (theor* OR concept* OR principle? OR polic* OR plan* OR strateg* OR objective? OR aim? OR goal? OR target? OR requirement? OR framework? OR model? OR assessment? OR appraisal? OR evaluation? OR approach? OR practice? OR tool?)) AND pd(20230101-20240712)	N/A	0
#25	TITLE,ABSTRACT,SUBJECT((“positive outcome?” OR “net positive outcome?” OR “positive impact?” OR “net positive impact?” OR “positive development?”) PRE/0 (theor* OR concept* OR principle? OR polic* OR plan* OR strateg* OR objective? OR aim? OR goal? OR target? OR requirement? OR framework? OR model? OR assessment? OR appraisal? OR evaluation? OR approach? OR practice? OR tool?))	10	10
	TITLE,ABSTRACT,SUBJECT((“positive outcome?” OR “net positive outcome?” OR “positive impact?” OR “net positive impact?” OR “positive development?”) PRE/0 (theor* OR concept* OR principle? OR polic* OR plan* OR strateg* OR objective? OR aim? OR goal? OR target? OR requirement? OR framework? OR model? OR assessment? OR appraisal? OR evaluation? OR approach? OR practice? OR tool?)) AND pd(20230101-20240712)	N/A	2
#26	TITLE,ABSTRACT,SUBJECT((“regenerative design?” OR “regenerative development?” OR “regenerative sustainability” OR “no net loss*” OR “mitigation hierarch*”) PRE/0 (theor* OR concept* OR principle? OR polic* OR plan* OR strateg* OR objective? OR aim? OR goal? OR target? OR requirement? OR framework? OR model? OR assessment? OR appraisal? OR evaluation? OR approach? OR practice? OR tool?))	5	5
	TITLE,ABSTRACT,SUBJECT((“regenerative design?” OR “regenerative development?” OR “regenerative sustainability” OR “no net loss*” OR “mitigation hierarch*”) PRE/0 (theor* OR concept* OR principle? OR polic* OR plan* OR strateg* OR objective? OR aim? OR goal? OR target? OR requirement? OR framework? OR model? OR assessment? OR appraisal? OR evaluation? OR approach? OR practice? OR tool?)) AND pd(20230101-20240712)	N/A	1
	**Total records retrieved (sum #22-26)**	**275**	**286**
	**Total records retrieved (sum #22-26 for date range 20230101-20240712)**	**N/A**	**14**

**Table:**

Sociological Abstracts (ProQuest)			
Searches conducted: August 17, 2023; July 12, 2024			
**Search**	**Query**	**Records retrieved (17/08/23)**	**Records retrieved (12/07/24)**
#1	TITLE,ABSTRACT,SUBJECT(“net outcome*” or “net gain*” or “net positive outcome*” or “net positive impact*” or “no net loss*” or “mitigation hierarch*” or “regenerative design*” or “regenerative development*” or “regenerative sustainability”)	157	159
#2	SUBJECT(“net positive*” or “net benefit*” or “positive development*”)	19	20
#3	[S1] or [S2]	176	179
#4	(MAINSUBJECT.EXACT.EXPLODE(“Theories” OR “Theory formation” OR “Public policy” OR “Policy implementation” OR “Strategies” OR “Planning”) OR MAINSUBJECT.EXACT(“Concepts” OR “Concept formation” OR “Principles” OR “Standards” OR “Policy making” OR “Methodological approaches”))	128,189	134,020
#5	SUBJECT(theor* or concept* or principle* or policy or policies or plan or plans or planning or strategy or strategies or objective* or aim* or goal* or target* or requirement* or framework* or model* or approach* or practice*)	487,149	508,793
#6	MAINSUBJECT.EXACT(“Social impact assessment”)	576	566
#7	TITLE,ABSTRACT,SUBJECT(“health risk assessment*” OR “impact assessment*” OR “environmental assessment*” OR “assessment tool*” OR “assessment method*”)	4149	4320
#8	TITLE,ABSTRACT,SUBJECT(HIA OR HIAs OR EIA OR EIAs OR “sustainability appraisal*”)	114	117
#9	(MAINSUBJECT.EXACT.EXPLODE(“Compensation”) OR MAINSUBJECT.EXACT(“Reparations” OR “Restitution”))	6274	6736
#10	TITLE,ABSTRACT,SUBJECT(mitigation* OR “hierarchical system*” OR “response hierarch*” OR compensation* OR offset* OR “off-set*” OR tradeoff* OR “trade-off*” OR reparation* OR restitution*)	17,016	17,522
#11	[S4] or [S5] or [S6] or [S7] or [S8] or [S9] or [S10]	509,442	531,592
#12	[S3] and [S11]	60	62
#13	TITLE,ABSTRACT,SUBJECT(health* OR wellbeing OR “well-being” OR welfare)	346,517	357,516
#14	(MAINSUBJECT.EXACT.EXPLODE(“Health” OR “Quality of life”) OR MAINSUBJECT.EXACT(“Health status” OR “Health planning” OR “Health disparities” OR “Well being” OR “Social welfare”))	108,390	115,704
#15	(MAINSUBJECT.EXACT.EXPLODE(“Social justice” OR “Social environment” OR “Social processes”) OR MAINSUBJECT.EXACT(“Social factors” OR “Social conditions” OR “Social problems” OR “Restorative justice” OR “Social impact” OR “Social inequality” OR “Socioeconomic factors” OR “Sociodemographics” OR “Society”))	167,975	179,572
#16	SUBJECT(social* OR societal* OR society OR societies OR sociodemographic* OR socioeconomic* OR sociological*)	895,673	927,572
#17	(MAINSUBJECT.EXACT.EXPLODE(“Population”) OR MAINSUBJECT.EXACT(“Residents” OR “Interest groups” OR “Community”))	37,006	39,920
#18	SUBJECT(human* OR population* OR stakeholder* OR community or communities)	215,498	233,506
#19	[S13] or [S14] or [S15] or [S16] or [S17] or [S18]	1,166,222	1,209,413
#20	[S3] and [S19]	125	128
#21	TITLE,ABSTRACT,SUBJECT((health* OR wellbeing OR “well-being” OR welfare OR soci* OR commun*) NEAR/2 net NEAR/2 (gain? OR benefit? OR improve* OR increase? OR positive?))	100	94
#22	[S21] and [S11]	52	48
	([S21] and [S11]) AND pd(20230101-20240712)	N/A	0
#23	[S12] or [S20]	140	144
	([S12] or [S20]) AND pd(20230101-20240712)	N/A	4
#24	TITLE,ABSTRACT,SUBJECT((“net outcome?” OR “net gain?” OR “net benefit?” OR “net improve*” OR “net increase?” OR “net positive?”) PRE/0 (theor* OR concept* OR principle? OR polic* OR plan* OR strateg* OR objective? OR aim? OR goal? OR target? OR requirement? OR framework? OR model? OR assessment? OR appraisal? OR evaluation? OR approach? OR practice? OR tool?))	2	2
	TITLE,ABSTRACT,SUBJECT((“net outcome?” OR “net gain?” OR “net benefit?” OR “net improve*” OR “net increase?” OR “net positive?”) PRE/0 (theor* OR concept* OR principle? OR polic* OR plan* OR strateg* OR objective? OR aim? OR goal? OR target? OR requirement? OR framework? OR model? OR assessment? OR appraisal? OR evaluation? OR approach? OR practice? OR tool?)) AND pd(20230101-20240712)	N/A	0
#25	TITLE,ABSTRACT,SUBJECT((“positive outcome?” OR “net positive outcome?” OR “positive impact?” OR “net positive impact?” OR “positive development?”) PRE/0 (theor* OR concept* OR principle? OR polic* OR plan* OR strateg* OR objective? OR aim? OR goal? OR target? OR requirement? OR framework? OR model? OR assessment? OR appraisal? OR evaluation? OR approach? OR practice? OR tool?))	14	16
	TITLE,ABSTRACT,SUBJECT((“positive outcome?” OR “net positive outcome?” OR “positive impact?” OR “net positive impact?” OR “positive development?”) PRE/0 (theor* OR concept* OR principle? OR polic* OR plan* OR strateg* OR objective? OR aim? OR goal? OR target? OR requirement? OR framework? OR model? OR assessment? OR appraisal? OR evaluation? OR approach? OR practice? OR tool?)) AND pd(20230101-20240712)	N/A	1
#26	TITLE,ABSTRACT,SUBJECT((“regenerative design?” OR “regenerative development?” OR “regenerative sustainability” OR “no net loss*” OR “mitigation hierarch*”) PRE/0 (theor* OR concept* OR principle? OR polic* OR plan* OR strateg* OR objective? OR aim? OR goal? OR target? OR requirement? OR framework? OR model? OR assessment? OR appraisal? OR evaluation? OR approach? OR practice? OR tool?))	4	4
	TITLE,ABSTRACT,SUBJECT((“regenerative design?” OR “regenerative development?” OR “regenerative sustainability” OR “no net loss*” OR “mitigation hierarch*”) PRE/0 (theor* OR concept* OR principle? OR polic* OR plan* OR strateg* OR objective? OR aim? OR goal? OR target? OR requirement? OR framework? OR model? OR assessment? OR appraisal? OR evaluation? OR approach? OR practice? OR tool?)) AND pd(20230101-20240712)	N/A	1
	**Total records retrieved (sum #22-26)**	**212**	**214**
	**Total records retrieved (sum #22-26 for date range 20230101-20240712)**	**N/A**	**6**

**Table:**

ASSIA (ProQuest)			
**Searches conducted: August 17, 2023; July 12, 2024**			
**Search**	**Query**	**Records retrieved (17/08/23)**	**Records retrieved (12/07/24)**
#1	TITLE,ABSTRACT,SUBJECT(“net outcome*” or “net gain*” or “net positive outcome*” or “net positive impact*” or “no net loss*” or “mitigation hierarch*” or “regenerative design*” or “regenerative development*” or “regenerative sustainability”)	71	72
#2	SUBJECT(“net positive*” or “net benefit*” or “positive development*”)	26	25
#3	[S1] or [S2]	97	97
#4	(MAINSUBJECT.EXACT.EXPLODE(“Theory” OR “Public policy” OR “Strategies” OR “Planning”) OR MAINSUBJECT.EXACT(“Conceptual models” OR “Concepts” OR “Concept formation” OR “Principles” OR “Standards” OR “Policy making” OR “Policy implementation” OR “Plans” OR “Professional practices” OR “Practice” OR “Approaches”))	57,821	62,376
#5	SUBJECT(theor* or concept* or principle* or policy or policies or plan or plans or planning or strategy or strategies or objective* or aim* or goal* or target* or requirement* or framework* or model* or approach* or practice*)	240,401	267,343
#6	MAINSUBJECT.EXACT(“Strategic impact assessment” OR “Health impact assessment” OR “Environmental impact assessment” OR “Strategic environmental assessment”)	156	159
#7	TITLE,ABSTRACT,SUBJECT(“health risk assessment*” OR “impact assessment*” OR “environmental assessment*” OR “assessment tool*” OR “assessment method*”)	11,529	12,044
#8	TITLE,ABSTRACT,SUBJECT(HIA OR HIAs OR EIA OR EIAs OR “sustainability appraisal*”)	175	178
#9	(MAINSUBJECT.EXACT.EXPLODE(“Compensation”) OR MAINSUBJECT.EXACT(“Mitigation” OR “Reparations”))	4013	4297
#10	TITLE,ABSTRACT,SUBJECT(mitigation* OR “hierarchical system*” OR “response hierarch*” OR compensation* OR offset* OR “off-set*” OR tradeoff* OR “trade-off*” OR reparation* OR restitution*)	10,293	10,624
#11	[S4] or [S5] or [S6] or [S7] or [S8] or [S9] or [S10]	260,571	288,200
#12	[S3] and [S11]	34	32
#13	TITLE,ABSTRACT,SUBJECT(health* OR wellbeing OR “well-being” OR welfare)	467,333	491,550
#14	(MAINSUBJECT.EXACT.EXPLODE(“Well being”) OR MAINSUBJECT.EXACT(“Health” OR “Public health” OR “Health status” OR “Health disparities” OR “Quality of life” OR “Welfare” OR “Social welfare”))	103,240	113,834
#15	(MAINSUBJECT.EXACT.EXPLODE(“Social justice” OR “Social processes”) OR MAINSUBJECT.EXACT(“Social factors” OR “Social conditions” OR “Social issues” OR “Social problems” OR “Distributive justice” OR “Restorative justice” OR “Social environment” OR “Social impact” OR “Social inequality” OR “Sociological aspects” OR “Socioeconomic factors” OR “Sociodemographics” OR “Society”))	49,102	56,584
#16	SUBJECT(social* OR societal* OR society OR societies OR sociodemographic* OR socioeconomic* OR sociological*)	230,317	254,262
#17	(MAINSUBJECT.EXACT.EXPLODE(“Population”) OR MAINSUBJECT.EXACT(“Humans” OR “Residents” OR “Local population” OR “Stakeholders” OR “Interest groups” OR “Community” OR “Local communities” OR “Master planned communities”))	66,045	68,498
#18	SUBJECT(human* OR population* OR stakeholder* OR community or communities)	174,765	187,926
#19	[S13] or [S14] or [S15] or [S16] or [S17] or [S18]	687,611	726,699
#20	[S3] and [S19]	65	62
#21	TITLE,ABSTRACT,SUBJECT((health* OR wellbeing OR “well-being” OR welfare OR soci* OR commun*) NEAR/2 net NEAR/2 (gain? OR benefit? OR improve* OR increase? OR positive?))	110	113
#22	[S21] and [S11]	36	38
	([S21] and [S11]) AND pd(20230101-20240712)	N/A	0
#23	TITLE,ABSTRACT,SUBJECT(“health net gain*” OR “net health gain*” OR “wellbeing net gain*” OR “net wellbeing gain*” OR “well-being net gain*” OR “net well-being gain*”)	5	5
#24	[S12] or [S20] or [S23]	78	74
	([S12] or [S20] or [S23]) AND pd(20230101-20240712)	N/A	1
#25	TITLE,ABSTRACT,SUBJECT((“net outcome?” OR “net gain?” OR “net benefit?” OR “net improve*” OR “net increase?” OR “net positive?”) PRE/0 (theor* OR concept* OR principle? OR polic* OR plan* OR strateg* OR objective? OR aim? OR goal? OR target? OR requirement? OR framework? OR model? OR assessment? OR appraisal? OR evaluation? OR approach? OR practice? OR tool?))	20	21
	TITLE,ABSTRACT,SUBJECT((“net outcome?” OR “net gain?” OR “net benefit?” OR “net improve*” OR “net increase?” OR “net positive?”) PRE/0 (theor* OR concept* OR principle? OR polic* OR plan* OR strateg* OR objective? OR aim? OR goal? OR target? OR requirement? OR framework? OR model? OR assessment? OR appraisal? OR evaluation? OR approach? OR practice? OR tool?)) AND pd(20230101-20240712)	N/A	1
#26	TITLE,ABSTRACT,SUBJECT((“positive outcome?” OR “net positive outcome?” OR “positive impact?” OR “net positive impact?” OR “positive development?”) PRE/0 (theor* OR concept* OR principle? OR polic* OR plan* OR strateg* OR objective? OR aim? OR goal? OR target? OR requirement? OR framework? OR model? OR assessment? OR appraisal? OR evaluation? OR approach? OR practice? OR tool?))	17	18
	TITLE,ABSTRACT,SUBJECT((“positive outcome?” OR “net positive outcome?” OR “positive impact?” OR “net positive impact?” OR “positive development?”) PRE/0 (theor* OR concept* OR principle? OR polic* OR plan* OR strateg* OR objective? OR aim? OR goal? OR target? OR requirement? OR framework? OR model? OR assessment? OR appraisal? OR evaluation? OR approach? OR practice? OR tool?)) AND pd(20230101-20240712)	N/A	2
#27	TITLE,ABSTRACT,SUBJECT((“regenerative design?” OR “regenerative development?” OR “regenerative sustainability” OR “no net loss*” OR “mitigation hierarch*”) PRE/0 (theor* OR concept* OR principle? OR polic* OR plan* OR strateg* OR objective? OR aim? OR goal? OR target? OR requirement? OR framework? OR model? OR assessment? OR appraisal? OR evaluation? OR approach? OR practice? OR tool?))	0	0
	**Total records retrieved (sum #22, #24-27)**	**151**	**151**
	**Total records retrieved (sum #22, #24-27 for date range 20230101-20240712)**	**N/A**	**4**

**Table:**

Political Science Database (ProQuest)			
**Searches conducted: August 17, 2023; July 12, 2024**			
**Search**	**Query**	**Records retrieved (17/08/23)**	**Records retrieved (12/07/24)**
#1	TITLE,ABSTRACT,SUBJECT(“net outcome*” or “net gain*” or “net positive outcome*” or “net positive impact*” or “no net loss*” or “mitigation hierarch*” or “regenerative design*” or “regenerative development*” or “regenerative sustainability”)	103	105
#2	(MAINSUBJECT.EXACT(“Theory” OR “Principles” OR “Standards” OR “Public policy” OR “Policy making” OR “Planning” OR “Professional practice” OR “Guidelines”))	99,832	105,557
#3	SUBJECT(theor* or concept* or principle* or policy or policies or plan or plans or planning or strategy or strategies or objective* or aim* or goal* or target* or requirement* or framework* or model* or approach* or practice*)	527,036	557,288
#4	MAINSUBJECT.EXACT(“Health risk assessment” OR “Environmental impact statements”)	4220	4380
#5	TITLE,ABSTRACT,SUBJECT(“health risk assessment*” OR “impact assessment*” OR “environmental assessment*” OR “assessment tool*” OR “assessment method*”)	4407	4627
#6	TITLE,ABSTRACT,SUBJECT(HIA OR HIAs OR EIA OR EIAs OR “sustainability appraisal*”)	382	385
#7	(MAINSUBJECT.EXACT(“Compensation” OR “Reparations” OR “Restitution”))	9379	9888
#8	TITLE,ABSTRACT,SUBJECT(mitigation* OR “hierarchical system*” OR “response hierarch*” OR compensation* OR offset* OR “off-set*” OR tradeoff* OR “trade-off*” OR reparation* OR restitution*)	28,108	29,328
#9	[S2] or [S3] or [S4] or [S5] or [S6] or [S7] or [S8]	557,837	589,303
#10	[S1] and [S9]	47	49
#11	TITLE,ABSTRACT,SUBJECT(health* OR wellbeing OR “well-being” OR welfare)	387,764	409,356
#12	(MAINSUBJECT.EXACT(“Health” OR “Public health” OR “Health disparities” OR “Quality of life” OR “Welfare”))	78,613	89,401
#13	(MAINSUBJECT.EXACT(“Social conditions & trends” OR “Social justice” OR “Distributive justice” OR “Restorative justice” OR “Social impact” OR “Socioeconomic factors” OR “Sociodemographics” OR “Society”))	65,901	68,819
#14	SUBJECT(social* OR societal* OR society OR societies OR sociodemographic* OR socioeconomic* OR sociological*)	304,742	326,162
#15	(MAINSUBJECT.EXACT(“Population” OR “Residents” OR “Stakeholders” OR “Interest groups” OR “Community” OR “Master planned communities”))	63,351	67,611
#16	SUBJECT(human* OR population* OR stakeholder* OR community or communities)	265,594	287,240
#17	[S11] or [S12] or [S13] or [S14] or [S15] or [S16]	792,922	842,361
#18	[S1] and [S17]	37	39
#19	TITLE,ABSTRACT,SUBJECT((health* OR wellbeing OR “well-being” OR welfare OR soci* OR commun*) NEAR/2 net NEAR/2 (gain? OR benefit? OR improve* OR increase? OR positive?))	84	87
#20	[S19] and [S9]	59	60
	([S19] and [S9]) AND pd(20230101-20240712)	N/A	2
#21	[S10] or [S18]	58	61
	([S10] or [S18]) AND pd(20230101-20240712)	N/A	4
#22	TITLE,ABSTRACT,SUBJECT((“net outcome?” OR “net gain?” OR “net benefit?” OR “net improve*” OR “net increase?” OR “net positive?”) PRE/0 (theor* OR concept* OR principle? OR polic* OR plan* OR strateg* OR objective? OR aim? OR goal? OR target? OR requirement? OR framework? OR model? OR assessment? OR appraisal? OR evaluation? OR approach? OR practice? OR tool?))	3	3
	TITLE,ABSTRACT,SUBJECT((“net outcome?” OR “net gain?” OR “net benefit?” OR “net improve*” OR “net increase?” OR “net positive?”) PRE/0 (theor* OR concept* OR principle? OR polic* OR plan* OR strateg* OR objective? OR aim? OR goal? OR target? OR requirement? OR framework? OR model? OR assessment? OR appraisal? OR evaluation? OR approach? OR practice? OR tool?)) AND pd(20230101-20240712)	N/A	0
#23	TITLE,ABSTRACT,SUBJECT((“positive outcome?” OR “net positive outcome?” OR “positive impact?” OR “net positive impact?” OR “positive development?”) PRE/0 (theor* OR concept* OR principle? OR polic* OR plan* OR strateg* OR objective? OR aim? OR goal? OR target? OR requirement? OR framework? OR model? OR assessment? OR appraisal? OR evaluation? OR approach? OR practice? OR tool?))	3	3
	TITLE,ABSTRACT,SUBJECT((“positive outcome?” OR “net positive outcome?” OR “positive impact?” OR “net positive impact?” OR “positive development?”) PRE/0 (theor* OR concept* OR principle? OR polic* OR plan* OR strateg* OR objective? OR aim? OR goal? OR target? OR requirement? OR framework? OR model? OR assessment? OR appraisal? OR evaluation? OR approach? OR practice? OR tool?)) AND pd(20230101-20240712)	N/A	1
#24	TITLE,ABSTRACT,SUBJECT((“regenerative design?” OR “regenerative development?” OR “regenerative sustainability” OR “no net loss*” OR “mitigation hierarch*”) PRE/0 (theor* OR concept* OR principle? OR polic* OR plan* OR strateg* OR objective? OR aim? OR goal? OR target? OR requirement? OR framework? OR model? OR assessment? OR appraisal? OR evaluation? OR approach? OR practice? OR tool?))	2	2
	TITLE,ABSTRACT,SUBJECT((“regenerative design?” OR “regenerative development?” OR “regenerative sustainability” OR “no net loss*” OR “mitigation hierarch*”) PRE/0 (theor* OR concept* OR principle? OR polic* OR plan* OR strateg* OR objective? OR aim? OR goal? OR target? OR requirement? OR framework? OR model? OR assessment? OR appraisal? OR evaluation? OR approach? OR practice? OR tool?)) AND pd(20230101-20240712)	N/A	1
	**Total records retrieved (sum #20-24)**	**125**	**129**
	**Total records retrieved (sum #20-24 for date range 20230101-20240712)**	**N/A**	**8**

**Table:**

Worldwide Political Science Abstracts (ProQuest)			
**Searches conducted: August 17, 2023; July 12, 2024**			
**Search**	**Query**	**Records retrieved (17/08/23)**	**Records retrieved (12/07/24)**
#1	TITLE,ABSTRACT,SUBJECT(“net outcome*” or “net gain*” or “net positive outcome*” or “net positive impact*” or “no net loss*” or “mitigation hierarch*” or “regenerative design*” or “regenerative development*” or “regenerative sustainability”)	110	114
#2	SUBJECT(“net positive*” or “net benefit*” or “positive development*”)	2	1
#3	[S1] or [S2]	112	115
#4	(MAINSUBJECT.EXACT(“Theory” OR “Principles” OR “Standards” OR “Public policy” OR “Policy making” OR “Planning” OR “Professional practice” OR “Guidelines”))	97,441	104,369
#5	SUBJECT(theor* or concept* or principle* or policy or policies or plan or plans or planning or strategy or strategies or objective* or aim* or goal* or target* or requirement* or framework* or model* or approach* or practice*)	476,609	504,065
#6	MAINSUBJECT.EXACT(“Health risk assessment” OR “Environmental impact statements”)	164	177
#7	TITLE,ABSTRACT,SUBJECT(“health risk assessment*” OR “impact assessment*” OR “environmental assessment*” OR “assessment tool*” OR “assessment method*”)	1178	1270
#8	TITLE,ABSTRACT,SUBJECT(HIA OR HIAs OR EIA OR EIAs OR “sustainability appraisal*”)	153	168
#9	(MAINSUBJECT.EXACT(“Compensation” OR “Reparations” OR “Restitution”))	3903	4386
#10	TITLE,ABSTRACT,SUBJECT(mitigation* OR “hierarchical system*” OR “response hierarch*” OR compensation* OR offset* OR “off-set*” OR tradeoff* OR “trade-off*” OR reparation* OR restitution*)	14,609	15,704
#11	[S4] or [S5] or [S6] or [S7] or [S8] or [S9] or [S10]	489,507	517,835
#12	[S3] and [S11]	46	51
#13	TITLE,ABSTRACT,SUBJECT(health* OR wellbeing OR “well-being” OR welfare)	80,690	87,558
#14	(MAINSUBJECT.EXACT(“Health” OR “Public health” OR “Health disparities” OR “Quality of life” OR “Welfare”))	16,819	18,966
#15	(MAINSUBJECT.EXACT(“Social conditions & trends” OR “Social justice” OR “Distributive justice” OR “Restorative justice” OR “Social impact” OR “Socioeconomic factors” OR “Sociodemographics” OR “Society”))	40,613	45,641
#16	SUBJECT(social* OR societal* OR society OR societies OR sociodemographic* OR socioeconomic* OR sociological*)	307,115	338,432
#17	(MAINSUBJECT.EXACT(“Population” OR “Residents” OR “Stakeholders” OR “Interest groups” OR “Community” OR “Master planned communities”))	23,335	26,570
#18	SUBJECT(human* OR population* OR stakeholder* OR community or communities)	98,276	113,626
#19	[S13] or [S14] or [S15] or [S16] or [S17] or [S18]	427,770	470,706
#20	[S3] and [S19]	34	36
#21	TITLE,ABSTRACT,SUBJECT((health* OR wellbeing OR “well-being” OR welfare OR soci* OR commun*) NEAR/2 net NEAR/2 (gain? OR benefit? OR improve* OR increase? OR positive?))	78	79
#22	[S21] and [S11]	51	51
	([S21] and [S11]) AND pd(20230101-20240712)	N/A	1
#23	[S12] or [S20]	59	63
	([S12] or [S20]) AND pd(20230101-20240712)	N/A	2
#24	TITLE,ABSTRACT,SUBJECT((“net outcome?” OR “net gain?” OR “net benefit?” OR “net improve*” OR “net increase?” OR “net positive?”) PRE/0 (theor* OR concept* OR principle? OR polic* OR plan* OR strateg* OR objective? OR aim? OR goal? OR target? OR requirement? OR framework? OR model? OR assessment? OR appraisal? OR evaluation? OR approach? OR practice? OR tool?))	2	2
	TITLE,ABSTRACT,SUBJECT((“net outcome?” OR “net gain?” OR “net benefit?” OR “net improve*” OR “net increase?” OR “net positive?”) PRE/0 (theor* OR concept* OR principle? OR polic* OR plan* OR strateg* OR objective? OR aim? OR goal? OR target? OR requirement? OR framework? OR model? OR assessment? OR appraisal? OR evaluation? OR approach? OR practice? OR tool?)) AND pd(20230101-20240712)	N/A	0
#25	TITLE,ABSTRACT,SUBJECT((“positive outcome?” OR “net positive outcome?” OR “positive impact?” OR “net positive impact?” OR “positive development?”) PRE/0 (theor* OR concept* OR principle? OR polic* OR plan* OR strateg* OR objective? OR aim? OR goal? OR target? OR requirement? OR framework? OR model? OR assessment? OR appraisal? OR evaluation? OR approach? OR practice? OR tool?))	2	2
	TITLE,ABSTRACT,SUBJECT((“positive outcome?” OR “net positive outcome?” OR “positive impact?” OR “net positive impact?” OR “positive development?”) PRE/0 (theor* OR concept* OR principle? OR polic* OR plan* OR strateg* OR objective? OR aim? OR goal? OR target? OR requirement? OR framework? OR model? OR assessment? OR appraisal? OR evaluation? OR approach? OR practice? OR tool?)) AND pd(20230101-20240712)	N/A	0
#26	TITLE,ABSTRACT,SUBJECT((“regenerative design?” OR “regenerative development?” OR “regenerative sustainability” OR “no net loss*” OR “mitigation hierarch*”) PRE/0 (theor* OR concept* OR principle? OR polic* OR plan* OR strateg* OR objective? OR aim? OR goal? OR target? OR requirement? OR framework? OR model? OR assessment? OR appraisal? OR evaluation? OR approach? OR practice? OR tool?))	2	2
	TITLE,ABSTRACT,SUBJECT((“regenerative design?” OR “regenerative development?” OR “regenerative sustainability” OR “no net loss*” OR “mitigation hierarch*”) PRE/0 (theor* OR concept* OR principle? OR polic* OR plan* OR strateg* OR objective? OR aim? OR goal? OR target? OR requirement? OR framework? OR model? OR assessment? OR appraisal? OR evaluation? OR approach? OR practice? OR tool?)) AND pd(20230101-20240712)	N/A	1
	**Total records retrieved (sum #22-26)**	**116**	**120**
	**Total records retrieved (sum #22-26 for date range 20230101-20240712)**	**N/A**	**4**

**Table:**

Policy File (ProQuest)			
**Searches conducted: August 17, 2023; July 12, 2024**			
**Search**	**Query**	**Records retrieved (17/08/23)**	**Records retrieved (12/07/24)**
#1	TITLE,ABSTRACT,SUMMARY(“net outcome*” or “net gain*” or “net positive outcome*” or “net positive impact*” or “no net loss*” or “mitigation hierarch*” or “regenerative design*” or “regenerative development*” or “regenerative sustainability”)	73	74
#2	SUBJECT(theor* or concept* or principle* or policy or policies or plan or plans or planning or strategy or strategies or objective* or aim* or goal* or target* or requirement* or framework* or model* or approach* or practice*)	125,785	126,796
#3	TITLE,ABSTRACT,SUMMARY(“health risk assessment*” OR “impact assessment*” OR “environmental assessment*” OR “assessment tool*” OR “assessment method*”)	491	508
#4	TITLE,ABSTRACT,SUMMARY(HIA OR HIAs OR EIA OR EIAs OR “sustainability appraisal*”)	94	95
#5	TITLE,ABSTRACT,SUMMARY(mitigation* OR “hierarchical system*” OR “response hierarch*” OR compensation* OR offset* OR “off-set*” OR tradeoff* OR “trade-off*” OR reparation* OR restitution*)	7483	7650
#6	[S2] or [S3] or [S4] or [S5]	129,939	131,082
#7	[S1] and [S6]	50	51
#8	TITLE,ABSTRACT,SUMMARY(health* OR wellbeing OR “well-being” OR welfare)	37,740	38,254
#9	SUBJECT(social* OR societal* OR society OR societies OR sociodemographic* OR socioeconomic* OR sociological*)	56,144	14,328
#10	SUBJECT(human* OR population* OR stakeholder* OR community or communities)	9,061	9,280
#11	[S8] or [S9] or [S10]	86,899	58,197
#12	[S1] and [S11]	22	16
#13	TITLE,ABSTRACT,SUMMARY((health* OR wellbeing OR “well-being” OR welfare OR soci* OR commun*) NEAR/2 net NEAR/2 (gain? OR benefit? OR improve* OR increase? OR positive?))	62	57
#14	[S13] and [S6]	35	31
	([S13] and [S6]) AND pd(20230101-20240712)	N/A	1
#15	[S7] or [S12]	58	55
	([S7] or [S12]) AND pd(20230101-20240712)	N/A	1
#16	TITLE,ABSTRACT,SUMMARY((“net outcome?” OR “net gain?” OR “net benefit?” OR “net improve*” OR “net increase?” OR “net positive?”) PRE/0 (theor* OR concept* OR principle? OR polic* OR plan* OR strateg* OR objective? OR aim? OR goal? OR target? OR requirement? OR framework? OR model? OR assessment? OR appraisal? OR evaluation? OR approach? OR practice? OR tool?))	0	0
#17	TITLE,ABSTRACT,SUMMARY((“positive outcome?” OR “net positive outcome?” OR “positive impact?” OR “net positive impact?” OR “positive development?”) PRE/0 (theor* OR concept* OR principle? OR polic* OR plan* OR strateg* OR objective? OR aim? OR goal? OR target? OR requirement? OR framework? OR model? OR assessment? OR appraisal? OR evaluation? OR approach? OR practice? OR tool?))	0	0
#18	TITLE,ABSTRACT,SUMMARY((“regenerative design?” OR “regenerative development?” OR “regenerative sustainability” OR “no net loss*” OR “mitigation hierarch*”) PRE/0 (theor* OR concept* OR principle? OR polic* OR plan* OR strateg* OR objective? OR aim? OR goal? OR target? OR requirement? OR framework? OR model? OR assessment? OR appraisal? OR evaluation? OR approach? OR practice? OR tool?))	0	0
	**Total records retrieved (sum #14-18)**	**93**	**86**
	**Total records retrieved (sum #14-18 for date range 20230101-20240712)**	**N/A**	**2**

**Table:**

Sociology Database (ProQuest)			
**Searches conducted: August 17, 2023; July 12, 2024**			
**Search**	**Query**	**Records retrieved (17/08/23)**	**Records retrieved (12/07/24)**
#1	TITLE,ABSTRACT,SUBJECT(“net outcome*” or “net gain*” or “net positive outcome*” or “net positive impact*” or “no net loss*” or “mitigation hierarch*” or “regenerative design*” or “regenerative development*” or “regenerative sustainability”)	46	45
#2	SUBJECT(“net positive*” or “net benefit*” or “positive development*”)	12	6
#3	[S1] or [S2]	58	51
#4	(MAINSUBJECT.EXACT(“Theory” OR “Principles” OR “Standards” OR “Public policy” OR “Policy making” OR “Planning” OR “Professional practice” OR “Guidelines”))	32,078	36,076
#5	SUBJECT(theor* or concept* or principle* or policy or policies or plan or plans or planning or strategy or strategies or objective* or aim* or goal* or target* or requirement* or framework* or model* or approach* or practice*)	159,639	185,964
#6	MAINSUBJECT.EXACT(“Health risk assessment” OR “Environmental impact statements”)	2752	2908
#7	TITLE,ABSTRACT,SUBJECT(“health risk assessment*” OR “impact assessment*” OR “environmental assessment*” OR “assessment tool*” OR “assessment method*”)	5016	5378
#8	TITLE,ABSTRACT,SUBJECT(HIA OR HIAs OR EIA OR EIAs OR “sustainability appraisal*”)	88	91
#9	(MAINSUBJECT.EXACT(“Compensation” OR “Reparations” OR “Restitution”))	1548	1740
#10	TITLE,ABSTRACT,SUBJECT(mitigation* OR “hierarchical system*” OR “response hierarch*” OR compensation* OR offset* OR “off-set*” OR tradeoff* OR “trade-off*” OR reparation* OR restitution*)	7019	7396
#11	[S4] or [S5] or [S6] or [S7] or [S8] or [S9] or [S10]	169,455	196,803
#12	[S3] and [S11]	22	21
#13	TITLE,ABSTRACT,SUBJECT(health* OR wellbeing OR “well-being” OR welfare)	197,621	216,573
#14	(MAINSUBJECT.EXACT(“Health” OR “Public health” OR “Health disparities” OR “Quality of life” OR “Welfare”))	39,891	46,407
#15	(MAINSUBJECT.EXACT(“Social conditions & trends” OR “Social justice” OR “Distributive justice” OR “Restorative justice” OR “Social impact” OR “Socioeconomic factors” OR “Sociodemographics” OR “Society”))	52,234	57,025
#16	SUBJECT(social* OR societal* OR society OR societies OR sociodemographic* OR socioeconomic* OR sociological*)	225,380	256,513
#17	(MAINSUBJECT.EXACT(“Population” OR “Residents” OR “Stakeholders” OR “Interest groups” OR “Community” OR “Master planned communities”))	26,857	30,055
#18	SUBJECT(human* OR population* OR stakeholder* OR community or communities)	150,610	170,557
#19	[S13] or [S14] or [S15] or [S16] or [S17] or [S18]	414,741	457,483
#20	[S3] and [S19]	34	30
#21	TITLE,ABSTRACT,SUBJECT((health* OR wellbeing OR “well-being” OR welfare OR soci* OR commun*) NEAR/2 net NEAR/2 (gain? OR benefit? OR improve* OR increase? OR positive?))	30	27
#22	[S21] and [S11]	14	12
	([S21] and [S11]) AND pd(20230101-20240712)	N/A	1
#23	[S12] or [S20]	42	36
	([S12] or [S20]) AND pd(20230101-20240712)	N/A	3
#24	TITLE,ABSTRACT,SUBJECT((“net outcome?” OR “net gain?” OR “net benefit?” OR “net improve*” OR “net increase?” OR “net positive?”) PRE/0 (theor* OR concept* OR principle? OR polic* OR plan* OR strateg* OR objective? OR aim? OR goal? OR target? OR requirement? OR framework? OR model? OR assessment? OR appraisal? OR evaluation? OR approach? OR practice? OR tool?))	8	8
	TITLE,ABSTRACT,SUBJECT((“net outcome?” OR “net gain?” OR “net benefit?” OR “net improve*” OR “net increase?” OR “net positive?”) PRE/0 (theor* OR concept* OR principle? OR polic* OR plan* OR strateg* OR objective? OR aim? OR goal? OR target? OR requirement? OR framework? OR model? OR assessment? OR appraisal? OR evaluation? OR approach? OR practice? OR tool?)) AND pd(20230101-20240712)	N/A	0
#25	TITLE,ABSTRACT,SUBJECT((“positive outcome?” OR “net positive outcome?” OR “positive impact?” OR “net positive impact?” OR “positive development?”) PRE/0 (theor* OR concept* OR principle? OR polic* OR plan* OR strateg* OR objective? OR aim? OR goal? OR target? OR requirement? OR framework? OR model? OR assessment? OR appraisal? OR evaluation? OR approach? OR practice? OR tool?))	8	9
	TITLE,ABSTRACT,SUBJECT((“positive outcome?” OR “net positive outcome?” OR “positive impact?” OR “net positive impact?” OR “positive development?”) PRE/0 (theor* OR concept* OR principle? OR polic* OR plan* OR strateg* OR objective? OR aim? OR goal? OR target? OR requirement? OR framework? OR model? OR assessment? OR appraisal? OR evaluation? OR approach? OR practice? OR tool?)) AND pd(20230101-20240712)	N/A	1
#26	TITLE,ABSTRACT,SUBJECT((“regenerative design?” OR “regenerative development?” OR “regenerative sustainability” OR “no net loss*” OR “mitigation hierarch*”) PRE/0 (theor* OR concept* OR principle? OR polic* OR plan* OR strateg* OR objective? OR aim? OR goal? OR target? OR requirement? OR framework? OR model? OR assessment? OR appraisal? OR evaluation? OR approach? OR practice? OR tool?))	2	2
	TITLE,ABSTRACT,SUBJECT((“regenerative design?” OR “regenerative development?” OR “regenerative sustainability” OR “no net loss*” OR “mitigation hierarch*”) PRE/0 (theor* OR concept* OR principle? OR polic* OR plan* OR strateg* OR objective? OR aim? OR goal? OR target? OR requirement? OR framework? OR model? OR assessment? OR appraisal? OR evaluation? OR approach? OR practice? OR tool?)) AND pd(20230101-20240712)	N/A	1
	**Total records retrieved (sum #22-26)**	**74**	**67**
	**Total records retrieved (sum #22-26 for date range 20230101-20240712)**	**N/A**	**6**

**Table:**

Social Services Abstracts (ProQuest)			
**Searches conducted: August 17, 2023; July 12, 2024**			
**Search**	**Query**	**Records retrieved (17/08/23)**	**Records retrieved (12/07/24)**
#1	TITLE,ABSTRACT,SUBJECT(“net outcome*” or “net gain*” or “net positive outcome*” or “net positive impact*” or “no net loss*” or “mitigation hierarch*” or “regenerative design*” or “regenerative development*” or “regenerative sustainability”)	24	24
#2	SUBJECT(“net positive*” or “net benefit*” or “positive development*”)	13	15
#3	[S1] or [S2]	37	39
#4	(MAINSUBJECT.EXACT.EXPLODE(“Theories” OR “Theory formation” OR “Public policy” OR “Policy implementation” OR “Strategies” OR “Planning”) OR MAINSUBJECT.EXACT(“Concepts” OR “Concept formation” OR “Principles” OR “Standards” OR “Policy making” OR “Methodological approaches”))	35,091	37,247
#5	SUBJECT(theor* or concept* or principle* or policy or policies or plan or plans or planning or strategy or strategies or objective* or aim* or goal* or target* or requirement* or framework* or model* or approach* or practice*)	140,785	148,870
#6	MAINSUBJECT.EXACT(“Social impact assessment”)	248	248
#7	TITLE,ABSTRACT,SUBJECT(“health risk assessment*” OR “impact assessment*” OR “environmental assessment*” OR “assessment tool*” OR “assessment method*”)	3671	3822
#8	TITLE,ABSTRACT,SUBJECT(HIA OR HIAs OR EIA OR EIAs OR “sustainability appraisal*”)	65	65
#9	(MAINSUBJECT.EXACT.EXPLODE(“Compensation”) OR MAINSUBJECT.EXACT(“Reparations” OR “Restitution”))	2207	2351
#10	TITLE,ABSTRACT,SUBJECT(mitigation* OR “hierarchical system*” OR “response hierarch*” OR compensation* OR offset* OR “off-set*” OR tradeoff* OR “trade-off*” OR reparation* OR restitution*)	3431	3541
#11	[S4] or [S5] or [S6] or [S7] or [S8] or [S9] or [S10]	149,183	157,551
#12	[S3] and [S11]	18	21
#13	TITLE,ABSTRACT,SUBJECT(health* OR wellbeing OR “well-being” OR welfare)	249,257	258,708
#14	(MAINSUBJECT.EXACT.EXPLODE(“Health” OR “Quality of life”) OR MAINSUBJECT.EXACT(“Health status” OR “Health planning” OR “Health disparities” OR “Well being” OR “Social welfare”))	72,704	79,263
#15	(MAINSUBJECT.EXACT.EXPLODE(“Social justice” OR “Social environment” OR “Social processes”) OR MAINSUBJECT.EXACT(“Social factors” OR “Social conditions” OR “Social problems” OR “Restorative justice” OR “Social impact” OR “Social inequality” OR “Socioeconomic factors” OR “Sociodemographics” OR “Society”))	32,098	35,180
#16	SUBJECT(social* OR societal* OR society OR societies OR sociodemographic* OR socioeconomic* OR sociological*)	257,857	268,902
#17	(MAINSUBJECT.EXACT.EXPLODE(“Population”) OR MAINSUBJECT.EXACT(“Residents” OR “Interest groups” OR “Community”))	11,345	12,613
#18	SUBJECT(human* OR population* OR stakeholder* OR community or communities)	73,420	78,604
#19	[S13] or [S14] or [S15] or [S16] or [S17] or [S18]	385,838	400,569
#20	[S3] and [S19]	33	32
#21	TITLE,ABSTRACT,SUBJECT((health* OR wellbeing OR “well-being” OR welfare OR soci* OR commun*) NEAR/2 net NEAR/2 (gain? OR benefit? OR improve* OR increase? OR positive?))	41	42
#22	[S21] and [S11]	23	25
	([S21] and [S11]) AND pd(20230101-20240712)	N/A	0
#23	[S12] or [S20]	34	36
	([S12] or [S20]) AND pd(20230101-20240712)	N/A	2
#24	TITLE,ABSTRACT,SUBJECT((“net outcome?” OR “net gain?” OR “net benefit?” OR “net improve*” OR “net increase?” OR “net positive?”) PRE/0 (theor* OR concept* OR principle? OR polic* OR plan* OR strateg* OR objective? OR aim? OR goal? OR target? OR requirement? OR framework? OR model? OR assessment? OR appraisal? OR evaluation? OR approach? OR practice? OR tool?))	3	3
	TITLE,ABSTRACT,SUBJECT((“net outcome?” OR “net gain?” OR “net benefit?” OR “net improve*” OR “net increase?” OR “net positive?”) PRE/0 (theor* OR concept* OR principle? OR polic* OR plan* OR strateg* OR objective? OR aim? OR goal? OR target? OR requirement? OR framework? OR model? OR assessment? OR appraisal? OR evaluation? OR approach? OR practice? OR tool?)) AND pd(20230101-20240712)	N/A	0
#25	TITLE,ABSTRACT,SUBJECT((“positive outcome?” OR “net positive outcome?” OR “positive impact?” OR “net positive impact?” OR “positive development?”) PRE/0 (theor* OR concept* OR principle? OR polic* OR plan* OR strateg* OR objective? OR aim? OR goal? OR target? OR requirement? OR framework? OR model? OR assessment? OR appraisal? OR evaluation? OR approach? OR practice? OR tool?))	9	9
	TITLE,ABSTRACT,SUBJECT((“positive outcome?” OR “net positive outcome?” OR “positive impact?” OR “net positive impact?” OR “positive development?”) PRE/0 (theor* OR concept* OR principle? OR polic* OR plan* OR strateg* OR objective? OR aim? OR goal? OR target? OR requirement? OR framework? OR model? OR assessment? OR appraisal? OR evaluation? OR approach? OR practice? OR tool?)) AND pd(20230101-20240712)	N/A	0
#26	TITLE,ABSTRACT,SUBJECT((“regenerative design?” OR “regenerative development?” OR “regenerative sustainability” OR “no net loss*” OR “mitigation hierarch*”) PRE/0 (theor* OR concept* OR principle? OR polic* OR plan* OR strateg* OR objective? OR aim? OR goal? OR target? OR requirement? OR framework? OR model? OR assessment? OR appraisal? OR evaluation? OR approach? OR practice? OR tool?))	0	0
	**Total records retrieved (sum #22-26)**	**69**	**73**
	**Total records retrieved (sum #22-26 for date range 20230101-20240712)**	**N/A**	**2**

**Table:**

Google					
**Searches conducted: August 30-31, 2023; July 17, 2024**					
**Search**	**Query**	**Records reviewed (30-31/08/23)**	**Records retrieved (30-31/08/23)**	**Records reviewed (17/07/24)**	**Records retrieved (17/07/24)**
#1	(“net outcome” OR “net gain” OR “net positive outcome” OR “net positive impact” OR “no net loss” OR “mitigation hierarchy” OR “regenerative design” OR “regenerative development” OR “regenerative sustainability”) AND (theor* OR concept* OR principle* OR policy OR policies OR plan OR plans OR planning OR strategy OR strategies OR objective* OR aim* OR goal* OR target* OR requirement* OR framework* OR model * OR approach* OR practice* OR “health risk assessment” OR “impact assessment” OR “environmental assessment” OR “assessment tool” OR “assessment method” OR hia OR eia OR “sustainability appraisal” OR mitigation* OR “hierarchical system” OR “response hierarchy” OR compensation* OR offset* OR “off set” OR tradeoff* OR “trade off” OR reparation* OR restitution*)	8	1	10	0
#2	(“net outcome” OR “net gain” OR “net positive outcome” OR “net positive impact” OR “no net loss” OR “mitigation hierarchy” OR “regenerative design” OR “regenerative development” OR “regenerative sustainability”) AND (health OR wellbeing OR “well being” OR welfare)	247	11	327	2
#3	(health OR wellbeing OR “well being” OR welfare OR social OR societal OR society OR societies OR sociodemographic OR socioeconomic OR sociological OR community) AROUND(2) net AROUND(2) (gain OR benefit OR improvement OR increase OR positive)	56	3	63	0
#4	(“health net gain” OR “health net benefit” OR “health net improvement” OR “health net increase” OR “health net positive”)	100	5	23	0
#5	(“wellbeing net gain” OR “wellbeing net benefit” OR “wellbeing net improvement” OR “wellbeing net increase” OR “wellbeing net positive”)	7	2	2	0
#6	(“well being net gain” OR “well being net benefit” OR “well being net improvement” OR “well being net increase” OR “well being net positive”)	18	1	2	1
#7	(“welfare net gain” OR “welfare net benefit” OR “welfare net improvement” OR “welfare net increase” OR “welfare net positive”)	72	0	1	0
#8	(“social net gain” OR “social net benefit” OR “social net improvement” OR “social net increase” OR “social net positive”)	130	2	58	1
#9	(“societal net gain” OR “societal net benefit” OR “societal net improvement” OR “societal net increase” OR “societal net positive”)	120	0	25	0
#10	(“society net gain” OR “society net benefit” OR “society net improvement” OR “society net increase” OR “society net positive”)	116	0	15	0
#11	(“societies net gain” OR “societies net benefit” OR “societies net improvement” OR “societies net increase” OR “societies net positive”)	9	0	0	0
#12	(“sociodemographic net gain” OR “sociodemographic net benefit” OR “sociodemographic net improvement” OR “sociodemographic net increase” OR “sociodemographic net positive”)	0	0	0	0
#13	(“socioeconomic net gain” OR “socioeconomic net benefit” OR “socioeconomic net improvement” OR “socioeconomic net increase” OR “socioeconomic net positive”)	32	0	1	0
#14	(“sociological net gain” OR “sociological net benefit” OR “sociological net improvement” OR “sociological net increase” OR “sociological net positive”)	0	0	0	0
#15	(“community net gain” OR “community net benefit” OR “community net improvement” OR “community net increase” OR “community net positive”)	109	1	6	0
#16	(“net health gain” OR “net health benefit” OR “net health improvement” OR “net health increase” OR “net health positive”)	112	0	144	0
#17	(“net wellbeing gain” OR “net wellbeing benefit” OR “net wellbeing improvement” OR “net wellbeing increase” OR “net wellbeing positive”)	14	0	2	0
#18	(“net well being gain” OR “net well being benefit” OR “net well being improvement” OR “net well being increase” OR “net well being positive”)	18	0	1	0
#19	(“net welfare gain” OR “net welfare benefit” OR “net welfare improvement” OR “net welfare increase” OR “net welfare positive”)	123	0	104	0
#20	(“net social gain” OR “net social benefit” OR “net social improvement” OR “net social increase” OR “net social positive”)	138	0	161	0
#21	(“net societal gain” OR “net societal benefit” OR “net societal improvement” OR “net societal increase” OR “net societal positive”)	162	0	105	0
#22	(“net society gain” OR “net society benefit” OR “net society improvement” OR “net society increase” OR “net society positive”)	25	0	0	0
#23	(“net societies gain” OR “net societies benefit” OR “net societies improvement” OR “net societies increase” OR “net societies positive”)	1	0	0	0
#24	(“net sociodemographic gain” OR “net sociodemographic benefit” OR “net sociodemographic improvement” OR “net sociodemographic increase” OR “net sociodemographic positive”)	0	0	0	0
#25	(“net socioeconomic gain” OR “net socioeconomic benefit” OR “net socioeconomic improvement” OR “net socioeconomic increase” OR “net socioeconomic positive”)	84	0	0	0
#26	(“net sociological gain” OR “net sociological benefit” OR “net sociological improvement” OR “net sociological increase” OR “net sociological positive”)	0	0	0	0
#27	(“net community gain” OR “net community benefit” OR “net community improvement” OR “net community increase” OR “net community positive”)	128	6	170	4
	**Total records retrieved (sum)**	**1,829**	**43**	**1,220**	**8**

Note: Google search results presented over multiple pages often contained fewer records than Google’s headline search result numbers; therefore, records reviewed correspond to the actual total number of records returned within search result pages and reviewed. Records retrieved correspond to information sources selected for screening. A date filter (Tools: Past year) was applied to all search query results for the searches conducted on July 17, 2024.

**Table:**

HMIC (Ovid)			
**Searches conducted: August 17, 2023; July 12, 2024**			
**Search**	**Query**	**Records retrieved (17/08/23)**	**Records retrieved (12/07/24)**
#1	(“net outcome*” or “net gain*” or “net positive outcome*” or “net positive impact*” or “no net loss*” or “mitigation hierarch*” or “regenerative design*” or “regenerative development*” or “regenerative sustainability”).mp.	24	24
#2	exp theory/ or exp frames of reference/ or conceptualisation/ or exp policy/ or exp policy formulation/ or policy development/ or implementation/ or strategy/ or strategy formulation/ or exp plans/ or exp planning/ or professional practice/	63,515	64,504
#3	(theor* or concept* or principle* or policy or policies or plan or plans or planning or strategy or strategies or objective* or aim* or goal* or target* or requirement* or framework* or model* or approach* or practice*).hw.	63,610	64,401
#4	environmental assessment/ or assessment methods/ or health impact assessment/ or health risk assessment/	897	943
#5	(“health risk assessment*” or “impact assessment*” or “environmental assessment*” or “assessment tool*” or “assessment method*”).mp.	2246	2315
#6	(HIA or HIAs or EIA or EIAs or “sustainability appraisal*”).mp.	122	125
#7	compensation/ or restitution/	428	438
#8	(mitigation* or “hierarchical system*” or “response hierarch*” or compensation* or offset* or “off-set*” or tradeoff* or “trade-off*” or reparation* or restitution*).mp.	2098	2146
#9	2 or 3 or 4 or 5 or 6 or 7 or 8	85,885	87,146
#10	((“net outcome*” or “net gain*” or “net benefit*” or “net improve*” or “net increase*” or “net positive*” or “positive outcome*” or “net positive outcome*” or “positive impact*” or “net positive impact*” or “positive development*” or “regenerative design*” or “regenerative development*” or “regenerative sustainability” or “no net loss*” or “mitigation hierarch*”) adj (theor* or concept* or principle* or polic* or plan* or strateg* or objective* or aim* or goal* or target* or requirement* or framework* or model* or assessment* or appraisal* or evaluation* or approach* or practice* or tool*)).mp.	5	5
#11	(1 and 9) or 10	9	9
#12	(health* or wellbeing or “well-being” or welfare).mp.	209,186	212,093
#13	exp health/ or health effects/ or exp health outcomes/ or exp health planning/ or quality of life/ or exp social welfare/	35,766	36,567
#14	exp social factors/ or exp social problems/ or social justice/ or exp socioeconomic factors/ or exp sociodemographic factors/ or exp social processes/ or society/	67,928	69,205
#15	(social* or societal* or society or societies or sociodemographic* or socioeconomic* or sociological*).hw.	45,871	46,444
#16	people/ or exp population/ or stakeholders/ or pressure groups/ or exp communities/	12,215	12,246
#17	(human* or population* or stakeholder* or community or communities).hw.	27,347	27,632
#18	12 or 13 or 14 or 15 or 16 or 17	267,290	270,625
#19	1 and 18	20	20
#20	((health* or wellbeing or “well-being” or welfare or soci* or commun*) adj3 net adj3 (gain* or benefit* or improve* or increase* or positive*)).mp.	41	41
#21	9 and 20	13	13
#22	(“health net gain*” or “net health gain*” or “wellbeing net gain*” or “net wellbeing gain*” or “well-being net gain*” or “net well-being gain*”).mp.	3	3
#23	11 or 19 or 21 or 22	39	39
#24	limit 23 to yr=2023-2024	N/A	0

## Appendix II: Evidence sources ineligible following full-text review

As 542 evidence sources were assessed for eligibility, individual evidence sources were not listed. Reasons for exclusion and the number of evidence sources excluded are summarized below. Further details are available on request.

**Table:**

PRISMA label	Exclusion reason hierarchy (Covidence)	From database searches	From citation searches	Total
Sources sought for retrieval	N/A	486	70	556
Sources not retrieved	1 Source unavailable	12	2	14
Sources assessed for eligibility	N/A	474	68	542
Sources excluded:	2 Source removed at pre-screen (full-text irrelevant)	4	0	4
Ineligible context				
Sources excluded:	3 Does not describe an objective or features of an approach	11	0	11
Ineligible context				
Sources excluded:	4 No explicit net-outcome objective	143	41	184
No NOO				
Sources excluded:	5 No explicit health net-outcome objective	95	13	108
No health NOO				
Sources excluded:	6 Not unambiguously transferable	100	5	105
Untransferable				
Sources excluded:	7 Inapplicable to spatial planning and development	2	0	2
Ineligible context				
Sources excluded:	8 Excludes data extraction target information	2	0	2
Insufficient content				
Sources excluded:	9 Short form of an included source (effective duplicate)	5	0	5
Effective duplicate				
Sources excluded:	N/A (manually assigned duplicate)	0	2	2
Duplicate				
Sources ineligible	N/A	362	61	423
Sources included in review	N/A	112	7	119

N/A, not applicable; NOO, net-outcome objective.

## Appendix III: Data extraction instrument

*Italicized* text in the right-hand column summarizes reviewers’ data extraction guidance and categories of categorical data. Items that are amendments or additions to the original protocol are denoted in the left-hand column.

**Table:**

Evidence source details and characteristics	
Source #	
Citation details	Author(s) *(Vancouver reference style)*
	Publication date
	Title
	Journal (or other publication source)
	Volume
	Issue
	Pages
	Author (or other*) keywords (Addition to original protocol)
	**If no author keywords were specified, any listed keywords were added from i) publisher’s websites or ii) Scopus (for journal articles and books)*
	**If no author keywords were specified, any listed key or subject words were added from i) institutional repositories or ii) ProQuest (for theses and gray literature)*
Source perspective	*A single category was assigned based on source title, journal, keywords, contributing authors’ institutional disciplines, and source content*
(Amendment to original protocol)	
	*Final categories:*
	*Assessment, conservation, corporate, design and development, extractive industries, philosophy, public health, regulation, security, spatial planning*
Country of origin	*A single country was assigned based on the location of the first author’s primary institution or, if not listed, the source’s place of publication*
Type of evidence source (document type)	*Final categories:*
	*Audiovisual material, book, book chapter, gray literature, journal article, thesis*
Document aims/purpose	
Type of net-outcome objective	*Final categories:*
(Addition to original protocol)	i) *A stand-alone health or well-being net-outcome objective (eg, health NG)*
	ii) *A net-outcome objective with a subsidiary/secondary health or well-being net-outcome objective (eg, biodiversity NG and well-being NWO)*
	iii) *A composite/multi-outcome net-outcome objective incorporating a specific health or well-being net-outcome objective*
	iv) *A stand-alone social or community net-outcome objective (eg, social NG, community NG)*
	v) *A net-outcome objective with a subsidiary/secondary social or community net-outcome objective (eg, BNG and social NWO)*
	vi) *A composite/multi-outcome net-outcome objective relevant to or encompassing health (eg, environmental NG, socioecological NG)*
	vii) *A generic net-outcome objective (derived from a non-health objective; eg, NG/NNL)*
	viii) *Universal features of or considerations for net-outcome objectives (eg, equity, offsetting, mitigation hierarchy)*
Health net-outcome objective, target, aim or goal	*Specified “net” objective, extracted short form, wherever stated as such (extracted for all sources that include a specific health net-outcome objective and any other [eg, biodiversity or nature-oriented] primary net-outcome objectives that present a secondary community, health, social, or well-being objective)*
Derived health net-outcome objective	*Coded shorthand for target and outcome based on preceding field above and source content*
(Addition to original protocol)	*Final targets: B=Biodiversity, H=Health, N=Nature, S=Social, W=Well-being*
	*Final outcomes: DNH+=do no harm or better off, NG=net gain, NWO=no worse off, NWO+=no worse off or better off*
Primary net-outcome objective, target, aim or goal	*Specified “net” objective, extracted short form, wherever stated as such (extracted for all sources)*
Derived primary net-outcome objective	*Coded shorthand for target and outcome based on preceding field above and source content*
(Addition to original protocol)	*Final targets: *=multi-objective, B=biodiversity, C=community, E=environmental, H=health, N=nature, S=social, W=well-being*
	*Final outcomes: NG=net gain, NNL=no-net-loss, NO=net-outcome*
	*Note: outcome precedes target (in brackets) for generic net-outcome objectives and universal features (refer to field: Type of net-outcome objective)*
Country/countries of application	*The nation(s) the objective (or related findings) applied to/were contextualized by, if not universal (noting that sources were considered to have universal applicability by default)*
Scale(s) of application	*Considering both:*
(Addition to original protocol)	i) *the scale(s) the source applied the objective to*
	ii) *the scale(s) the source discussed when exploring the objective*
	*Final categories: building, project, city, policy (local/regional), policy (national), policy (international), organization, system*
**Details/results extracted from source of evidence**	
Net-objective and approach: characteristics	
(Amendment to original protocol)	
Rationale(s) for net-outcome objective(s)	*Rationale for the net-outcome objective (specifically), if stated*
	*Rationale for the broader objective, if relevant and if no rationale was given for the net-outcome objective specifically*
(Amendment to original protocol)	
	*For net-outcome objectives that presented secondary community, health, social, or well-being objectives (refer to field: type of net-outcome objective), multiple objectives could be listed on separate lines*
Description(s) of net-outcome objective(s)	*Description/elaboration of the net-outcome objective that data extraction (subsequent fields) focused on*
(Amendment to original protocol)	*For net-outcome objectives that presented secondary community, health, social, or well-being objectives (refer to field: type of net-outcome objective), multiple objectives could be listed on separate lines*
	*The default approach was to focus extraction on community, health, social, or well-being objectives when specified (refer to field: type of net-outcome objective)*
Definition of objective’s health term(s)	*Definition of “health” and/or health term(s), if given*
Net-outcome definition/characterization and/or metric(s)	*Included extraction of summary commentary on composite/generic “net-outcome” measurement (but excluded extraction of full indicator breakdowns for composite objectives, for which only a summary precis and/or selected health/planning indicators were extracted; see following)*
(Addition to original protocol)	
	*Included key health or well-being indicators if a net-outcome objective was specifically applied to health or well-being (but excluded extraction of such indicators when they were mentioned more generally without reference to health net outcomes)*
	*If net-outcome definitions were extracted, supplementary elaborations of measurement requirements and procedural steps were recorded in the “Implementation principles and/or steps” field*
Net-outcome level emphasized	*The level of operationalization that extracted source data presented, emphasized or focused on*
(Addition to original protocol)	
	*Categorical levels of operationalization of net-outcome objectives were assigned post-extraction after reviewing the data extracted for each source*
	*Final categories:*
	1. *1**A Overarching requirement, 1B Overarching requirement (locally defined)*
	2. *Enhanced process*
	3. *3**A Assessed outcome, 3B Assessed outcome (locally defined)*
Specific metric(s) or broader framework(s)	*Specific metric(s) or broader framework(s), where specified (ie, beyond generic indicator themes (eg, environmental, economic, health) or methodological approaches (eg, qualitative, quantitative))*
(Addition to original protocol)	
	*Specific metric(s) or broader framework(s) described by each source were identified post-extraction after reviewing the data extracted for each source*
Implementation principle(s) and/or steps	*The concept of “implementation principles” of an objective and its associated approach was that implementation principles encompassed:*
	*• Principles, rules or requirements that governed implementation*
	*• Items framed as prerequisites, absolutes, essentials, necessary steps, imperatives, or propositions*
	*• Implementation frameworks, “how to” guides, “steps to take,” and action lists*
	*• Descriptions of how an objective was implemented in practice or would be implemented in practice*
	*• Descriptions of how an objective worked or operated in practice or how it would work or operate in practice*
	*• Descriptions of how an objective was or would be operationalized*
	*If a given implementation principle was framed as a possibility or one of a range of options, it was within the concept of “opportunities” (and not extracted as an “implementation principle”)*
	*The concept of “implementation principles” of an objective and its associated approach was that implementation principles generally excluded:*
	*i) Items that were not clearly linked to net-outcome objectives or approaches (eg, excluding principles related to broader/multi-outcome objectives, such as regenerative design and development, unless specific “net” principles were disaggregated*)*
	*ii) Items unambiguously unrelated or untransferable to health, community, social, broad environmental, or generic net-outcome contexts (eg, text addressing stand-alone ecological or resource-oriented implementation principles)*
	**If, however, a broader objective was defined solely by explicit net outcomes (such as the “positive development” objective), its principles were included*
	**If, however, the source was categorized as elaborating universal principles/implementation steps (ie, category in field: Type of net-outcome objective = 8), its principles were included*
Objective and approach: contextual positioning and effects (strengths, weaknesses, opportunities, threats [SWOT])	
(Amendment to original protocol)	
Positive effects or implications (strengths)	*The concept of “strengths” of an objective and its associated approach was that strengths:*
	*• were internal factors (ie, inherent to the objective and its associated approach)*
	*• were positive effects or implications*
	*• included observed, demonstrated, or proven positive effects or implications*
	*• included advantages versus the status quo or an existing comparator*
	*The concept of “strengths” of an objective and its associated approach was that strengths generally excluded:*
	*i) items that were not clearly linked to net-outcome objectives or approaches*
	*ii) items unambiguously unrelated or untransferable to health, community, social, broad environmental, or generic net-outcome contexts (eg, text addressing strengths related to stand-alone ecological or resource-oriented objectives)*
	*Extraction for composite and generic net-outcome objectives (refer to field: Type of net-outcome objective) focused on items contextualized by references to “net” phrases or concepts)*
	*Entries of “unstated” or “aggregated” were used if the concept was not addressed within the source or the source presented content not specific to net-outcome aspects of an objective or approach, respectively*
Negative effects or implications (weaknesses)	*The concept of “weaknesses” of an objective and its associated approach was that weaknesses:*
	*• were internal factors (ie, inherent to the objective and its associated approach)*
	*• were negative effects or implications*
	*• included observed, demonstrated, or proven negative effects or implications*
	*• included disadvantages versus the status quo or an existing comparator*
	*The concept of “weaknesses” of an objective and its associated approach was that weaknesses generally excluded:*
	*i) items that were not clearly linked to net-outcome objectives or approaches*
	*ii) items unambiguously unrelated or untransferable to health, community, social, broad environmental, or generic net-outcome contexts (eg, text addressing weaknesses related to stand-alone ecological or resource-oriented objectives)*
	*Extraction for composite and generic net-outcome objectives (refer to field: Type of net-outcome objective) focused on items contextualized by references to “net” phrases or concepts)*
	*Entries of “unstated” or “aggregated” were used if the concept was not addressed within the source or the source presented content not specific to net-outcome aspects of an objective or approach, respectively*
Implementation opportunities (opportunities)	*The concept of “opportunities” that applied to an objective and its associated approach was that opportunities:*
	*• were external factors (ie, not inherent to the objective and its associated approach)*
	*• were context-specific and related to its contextual positioning*
	*• included speculative implementation options and hypothetical future actions that might result in positive effects or implications*
	*The concept of “opportunities” that applied to an objective and its associated approach was that opportunities generally excluded:*
	*i) items that were not clearly linked to net-outcome objectives or approaches*
	*ii) items unambiguously unrelated or untransferable to health, community, social, broad environmental, or generic net-outcome contexts (eg, text addressing opportunities related to stand-alone ecological or resource-oriented objectives)*
	*Extraction for composite and generic net-outcome objectives (refer to field: Type of net-outcome objective) focused on items contextualized by references to “net” phrases or concepts*
	*Entries of “unstated” or “aggregated” were used if the concept was not addressed within the source or the source presented content not specific to net-outcome aspects of an objective or approach, respectively*
Implementation challenges (threats)	*The concept of “threats” that applied to an objective and its associated approach was that threats:*
	*• were external factors (ie, not inherent to the objective and its associated approach)*
	*• were context-specific and related to its contextual positioning*
	*• included speculative implementation options and hypothetical future actions that might result in negative effects or implications*
	*The concept of “threats” that applied to an objective and its associated approach was that threats generally excluded:*
	*i) Items that were not clearly linked to net-outcome objectives or approaches*
	*ii) Items unambiguously unrelated or untransferable to health, community, social, broad environmental, or generic net-outcome contexts (eg, text addressing threats related to stand-alone ecological or resource-oriented objectives)*
	*Extraction for composite and generic net-outcome objectives (refer to field: Type of net-outcome objective) focused on items contextualized by references to “net” phrases or concepts)*
	*Entries of “unstated” or “aggregated” were used if the concept was not addressed within the source or the source presented content not specific to net-outcome aspects of an objective or approach, respectively*
URL	*Direct weblink to source document (where available)*
(Addition to original protocol)	

BNG, biodiversity net gain; MH, mitigation hierarchy; NG, net gain; NNL, no net loss; NWO, no worse off.

Note: within free-text extracted data, square brackets were used to denote any clarifications added by reviewers to assist future readers. These include expanded acronyms, contextual clarifications, and prefixes identifying any verbatim text excerpts identified through large language model data extraction exercises (denoter: [AI]).

## Appendix IV: Characteristics of included evidence sources

**Table:**

Author, year, country	Document aims/purpose	Type of net-outcome objective (Table 1)	Derived health net-outcome objective	Derived primary net-outcome objectve	Country/countries of application	Scale(s) of application	Net-outcome level emphasized
ADDO, 2020, Australia^63^	To discuss the context for net community benefit and why we should not strive to develop a defined methodology for net community benefit assessment	4		C NG	Australia	Project, Policy (local/regional)	3B
Akturk, 2016, US^64^	To investigate the emerging field of regenerative design and development, its current theory and practice, and design support tools	6		* NG	Universal	Building, Project, System	3A
Andreucci *et al.*, 2021, EU^35^	To describe a new scenario and provide a practical guide for a restorative and regenerative approach to the built environment	3	HW NG	* NG	Universal	Building, Project, City, Policy (local/regional), Policy (national), Policy (international), Organization, System	1A
Australian Planners Declare, 2022, Australia^65^	To address an inquiry into the protections of the Victorian Planning Framework	4		C NG	Australia	Project, Policy (local/regional)	3B
Baker and McLellan, 1992, Canada^51^	To examine creative means to reduce conflict among disputing parties by viewing aggregate mining as a landscape opportunity	4		C NG	Universal	Project, Policy (local/regional)	2
Bateman and Zonneveld, 2019, UK^24^	To present options for using the Net Environmental Gain proposals to deliver on the 25 Year Environment Plan ambitions and to enhance social well-being through improved decision-making on the siting of environmental improvements	3		W * NG	UK	Project, Policy (national)	2
Berry and Steiker, 1974, US^66^	To explore the concept of fairness as a strategy for formulating decisions according to their distributional aspects	8		NG (S)	Universal	Project, Policy (local/regional)	1B
Birkeland, 2014, Australia^67^	To introduce an alternative positive framework for a more active and direct approach to sustainable design and assessment that de-couples environmental impacts and economic growth	6		* NG	Universal	Building, Project, City, System	3B
Birkeland, 2018, Australia^68^	To examine 2 documents, 1 representing sustainable urban policy and principles, the other representing urban biodiversity standards, against the Positive Development Test (whether the development increases the public estate, ecological base and future public options)	6		* NG	Universal	Building, Project, City, Policy (local/regional), Policy (national), Policy (international), System	3B
Birkeland, 2020, Australia^31^	To explain how to reform the anti-ecological biases in our current frameworks of environmental governance, planning, decision-making, and design	6		* NG	Universal	Building, Project, City, Policy (local/regional), Policy (national), Policy (international), Organization, System	3B
Birkeland, 2022, Australia^69^	To provide criteria for evaluating the potential of conventional and alternative methods for achieving nature-positive outcomes	6		* NG	Universal	Building, Project, City, Policy (local/regional), Policy (national), Policy (international), Organization, System	3B
Birkeland, 2023, Australia^70^	To distinguish STARfish [a new computer app for net-positive design] from rating tools and life-cycle analyses or “LCA”	6		* NG	Universal	Building, Project, System	3B
Bull *et al.*, 2018, UK^25^	To outline good practice principles for addressing the social impacts that arise from all losses and gains in biodiversity from a development project and its NNL/NG activities	2	BW NWO	B NO	Universal	Project, Organization	3B
Burns *et al.*, 2019, US^71^	To consider the nature of the governance problem in regenerative development	8		NG (H*)	Universal	Project, Policy (local/regional), Policy (national), Policy (international)	2
Camrass, 2020, Australia^72^	To investigate how futures concepts may further existing regenerative sustainability thinking	6		* NG	Universal	Building, Project, System	1A
Camrass, 2022, Australia^73^	To provide a systematic review of publications on urban regenerative thinking and practice, including across urban development stages and sectors	6		* NG	Universal	Building, Project, City, Policy (local/regional), Policy (national), Policy (international), System	1B
Camrass, 2023, Australia^74^	To provide 8 principles for thinking and practice drawn from extensive regenerative futures research	6		* NG	Universal	Project, City, System	1B
Carver, 2021, UK^75^	To account for the stabilization of net principles in policy frameworks by combining economic sociology, visual media analysis of the net diagram and political ecology	7		NO	Universal	Project, Policy (local/regional), Policy (national), Policy (international), Organization	1A
Castellas *et al.*, 2019, Australia^76^	Unstated	4		C NG	Universal	Project, Policy (local/regional), Policy (national), Policy (international), Organization	3A
Chang, 2021, UK^77^	Unstated	1	H NG	H NG	UK	Project, Policy (local/regional), Policy (national), System	2
Chang *et al.*, 2022, UK^45^	To support those working in the built environment and public health sectors to maximize health improvement through planning and land use decisions	1	H NG	H NG	Universal	Building, Project, Policy (local/regional), Policy (national)	1B
Chartered Institute of Ecology and Environmental Management (CIEEM), 2021, UK^29^	To summarize the core messages from all of the evidence gathered by this scoping study	2	BW DNH+	B NG	UK	Project, Policy (local/regional), Policy (national)	1A
Chartered Institute of Ecology and Environmental Management (CIEEM), 2021, UK^78^	To scope the drivers for incorporating well-being into biodiversity net gain good practice, the challenges of doing so and the possible solutions to overcoming these challenges. This report describes the responses to these consultations, with the aim to document what people told us, as faithfully as possible	2	BW DNH+	B NG	UK	Project, Policy (local/regional), Policy (national)	1A
Commission on Creating Healthy Cities, 2022, UK^46^	To investigate the links between urban matters and health and well-being	1	H NG	H NG	UK	Building, Project, City, Policy (local/regional), Policy (national), System	1B
de Sadeleer, 2023, Belgium^18^	To highlight far-reaching provisions of the Treaty on European Union, the Treaty on the Functioning of the European Union, and the Charter of Fundamental Rights; address the green transition and integration principle; and discuss climate and environmental conditionality, given the requirement that national investments should cause no “significant harm to environmental objectives”	7		NG (E)	EU	Policy (international)	1A
Department for Environment Food and Rural Affairs (Defra), 2018, UK^23^	To seek views on whether we should introduce mandatory requirements to the planning system in England so that development must deliver biodiversity net gain and to set out the proposed next steps for our longer term ambition to embed environmental net gain	6		E NG	UK	Building, Project, Policy (local/regional), Policy (national)	1A
Department for Environment Food and Rural Affairs (Defra), 2019, UK^79^	To provide a summary of net gain consultation responses and government response	6		E NG	UK	Project, Policy (local/regional), Policy (national)	1A
Department for Levelling Up Housing and Communities (DLUHC), 2023, UK^16^	To set out the government’s planning policies for England and how these should be applied and provide a framework within which locally prepared plans can provide for sufficient housing and other development in a sustainable manner	6		* NG	UK	Building, Project, Policy (local/regional), Policy (national)	3B
Drupsteen and Wakkee, 2024, Netherland^80^	To explore regenerative business models’ definition and characteristics	6		* NG	Universal	Organization, System	1A
Du Plessis and Brandon, 2015, South Africa^81^	To explore the ecological worldview and the guidelines it provides for how we interpret sustainability; as well as the strategies for the production of the built environment we need to follow if we are to adapt to coming changes in the planetary system and regenerate the world	6		* NG	Universal	Building, Project, City, System	1A
Einola, 2020, Finland^36^	To present the framework of regenerative design as the basis for future urban development in which sustainability could be re-imagined to a net-positive symbiosis between humans and nature	6		* NG	Universal	Building, Project, City, Policy (local/regional), Policy (national), Policy (international), System	1A
Eisenberg, 2016, US^82^	To compare the current regulatory goal of preventing or limiting known harm with the positive outcome goals of the Living Building Challenge	6		* NG	Universal	Building, Project, System	1A
Esteves, 2008, Australia^53^	To demonstrate the value of SIA [Social Impact Assessment] as a tool for setting priorities, assist the mine operations and local communities to understand what a ‘net positive’ impact means; provide company decision-makers with a rigorous basis on which to understand the nature and extent of their ‘social responsibility’, and the effects of their decisions	4		C NG	Universal	Project, Organization	3B
European Health Forum Gastein, 2022, EU^83^	To reflect on what needs to be done to reaffirm the relevance of health at the EU level	1	W NG	W NG	EU	Policy (international)	1A
Faccioli *et al.*, 2024, Italy^30^	To investigate social preferences for the distribution of the benefits of environmental policies	8		NG (E)	Universal	Project, Policy (local/regional), Policy (national)	2
Fauna and Flora International (FFI), 2021, UK^56^	To examine current approaches to prevent, mitigate and manage the adverse environmental impacts of development projects in complex multi-use landscapes	7		NG	Universal	Project, Policy (local/regional), Policy (national), Policy (international), Organization, System	2
Fristik, 2012, US^84^	To examine mitigation in terms of intention, interpretation, implementation, litigation and application	8		NO	US	Project, Policy (national)	2
Gastineau *et al.*, 2021, France^85^	To develop an environmental compensation policy that meets NNL and NWO objectives so that the well-being of individuals is integrated to the NNL policy	5	BS NWO	B NNL	Universal	Project, Policy (local/regional), Policy (national)	3A
Gebler *et al.*, 2022, Germany^86^	To i) analyze perceptions of impacts of socio-technical systems on absolute sustainability, ii) summarize knowledge on positive impact concepts, iii) identify design principles for positive impacts, iv) derive management functions and organizational prerequisites to enable positive impacts, v) propose a guiding framework and a definition for positive impact, and vi) discuss potential applications and further research demand	6		* NG	Universal	Project, Policy (local/regional), Policy (national), Policy (international), Organization, System	2
Gibberd, 2015, South Africa^87^	To develop an alternative approach to conceptualizing and measuring the built environment which forms the basis of a new assessment tool	6		* NG	Universal	Building, Project, City, Policy (local/regional)	3B
Globus-Harris, 2020, US^88^	To develop a model of environmental trade ratios with asymmetric information to explain why current policies fail to achieve goals of no-net-impact on the environment, as well as demonstrating that it may be impossible to achieve these goals	7		NNL (E)	Universal	Project, Policy (local/regional), Policy (national), Policy (international)	2
Greyson, 2010, UK^89^	To offer a selection of 7 simple “policy switches” (or “leverage points” in complex systems) that offer an expanded vision of people’s role on Earth and a whole-system change to implement it	6		* NG	Universal	Policy (national), Policy (international), System	1A
Griffiths, 2019, UK^90^	To explore how people’s use and non-use values of nature can be incorporated into the concept of biodiversity NNL	2	BW NWO	B NNL	Universal	Project, Policy (local/regional), Policy (national), Policy (international), Organization	3B
Griffiths *et al.*, 2019, UK^27^	To conceptualize the no-worse-off principle in the context of NNL of biodiversity	2	BW NWO	B NNL	Universal	Project, Policy (local/regional), Policy (national), Policy (international), Organization	3B
Griffiths *et al.*, 2020, UK^26^	To explore local people’s perceptions of the importance of cultural heritage to their wellbeing, how the developments affected their cultural heritage, and how these perceived impacts could be incorporated into NNL strategies	2	BW NWO	B NNL	Universal	Project, Policy (local/regional), Policy (national), Policy (international), Organization	3B
Hes *et al.*, 2018, Australia^91^	To assess the process of applying regenerative thinking and reflect on the outcomes for the participants and the design of the masterplan of a 680-hectare project, “Seacombe West”	6		E NG	Australia	Project	2
Hiedanpää *et al.*, 2023, Finland^92^	To examine the preconditions for eco-social compensation and the mitigation of the harmful effects of incremental encroachment in the context of land-use planning	6		* NNL	Finland	Project, Policy (local/regional)	2
HM Government, 2018, UK^93^	To deliver cleaner air and water in our cities and rural landscapes, protect threatened species and provide richer wildlife habitats	6		E NG	UK	Project, Policy (local/regional), Policy (national)	1A
Homes England, 2023, UK^94^	To achieve health net gain through good design policy and practices	1	H NG	H NG	UK	Building, Project, Policy (local/regional), Policy (national), System	2
Hooker, 2009, UK^95^	To explore “bad” objections to aggregation, some good objections to it, and some bad ways of reacting to the good objections	8		NG (S)	Universal	Building, Project, City, Policy (local/regional), Policy (national), Policy (international), Organization, System	1B
Hooper *et al.*, 2021, UK^96^	To explore a series of questions to implement effective net gain policies for the marine environment	6		E NG	Universal	Project, Policy (local/regional), Policy (national), Policy (international), System	1A
Jones, *et al.*, 2019, UK^28^	To highlight risks to local well-being where projects restrict access to biodiversity and ecosystem services in biodiversity offsets	2	BW NWO	B NO	Universal	Project, Policy (local/regional), Policy (national), Policy (international), Organization	3B
Jones *et al.*, 2022, US^97^	To discuss and demonstrate analyses to answer key questions for formulating effective mitigation policies and applying the mitigation hierarchy	2	BW NWO+	B NO	Universal	Project, Policy (local/regional), Policy (national), Policy (international), Organization	3B
Jones *et al.*, 2022, Australia^98^	To report on the needs to facilitate learning and uptake of benefit analysis in students from primary, secondary, post-graduate to professional schooling, as well as practitioner accreditation across industry, community and educational institutions	6		* NG	Universal	Building, Project, Policy (local/regional), Policy (national), Policy (international), Organization, System	3B
Kambo, 2019, Australia^99^	To discover how design methodologies and 3 components they encompass – design intent, practice and process – can embrace the tenets of the Ecological Worldview	6		* NG	Australia	Building, Project, City, System	2
Knight-Lenihan, 2017, New Zealand^100^	To propose a theoretical model to assess whether activities associated with urban development create net positive environmental benefits	6		E NG	Universal	Building, Project, City, Policy (national), Organization, System	3B
Koksal, 2021, UK^47^	Not stated	1	H NG	H NG	UK	Project, Policy (local/regional)	1A
Konietzko *et al.*, 2023, Netherlands^54^	To provide an initial framework for regenerative business models and differentiate them from sustainable or circular models	6		* NG	Universal	Organization, System	2
Lichtenthaler, 2023, Germany^101^	To develop the concept of positive sustainability or positainability as the next level of sustainability management	6		* NG	Universal	Organization	1A
Lovell, 2018, Australia^19^	To present different types of offsetting, and the main theoretical and practical disadvantages and advantages of offsetting	8		NNL (E)	Universal	Project, Policy (national), Policy (international), Organization	2
Mang and Reed, 2015, US^102^	To posit that realizing the potential of the “net-positive” concept depends on how practitioners define positive	7		NG	Universal	Building, Project, System	1B
Maron, 2018, Australia^103^	To examine policies with NNL and related goals, explore how to resolve conflicts between overarching and impact-specific NNL policies, and improve transparency about what NNL-type policies are actually designed to achieve	7		NNL	Universal	Project, Policy (local/regional), Policy (national), Policy (international), Organization	1A
Martens, 2011, Netherlands^104^	To provide an overview of directions an equity analysis, carried out within the context of a social cost-benefit analysis, could take	8		NG	Universal	Project	3B
Mauerhofer, 2021, Sweden^105^	To outline the EU’s and UN’s latest environmental principle towards net gains	7		NG (E)	Universal	Policy (international)	1A
McCord, 2023, NR^106^	To differentiate between achieving neutral impact and seeking net positive impact	6		* NG	Universal	Organization	3A
McLaren and Carver, 2023, UK^107^	To examine the history of “NNL” in biodiversity and biodiversity offsetting to suggest critical lessons that must be learned in climate policy if net-zero is to deliver on the expectations of the Paris Agreement	7		NO	Universal	Project, Policy (local/regional), Policy (national), Policy (international)	1A
Middle and Middle, 2010, Australia^108^	To review the use of offsets in the Western Australian environmental impact assessment process	8		NNL	Universal	Project, Policy (local/regional)	2
Mifsud and Caruso, 2024, Malta^109^	To consider the regenerative sustainability principles as a way to challenge the current speculation-driven development in Malta’s dense urban areas	6		* NG	Universal	Building, Project, City, Policy (local/regional), Policy (national)	1A
Ministry of Housing Communities and Local Government (MHCLG), 2020, UK^110^	To seek views on proposals for reform of the planning system in England to streamline and modernize the planning process, improve outcomes on design and sustainability, reform developer contributions and ensure more land is available for development where it is needed	6		* NG	UK	Building, Project, Policy (local/regional), Policy (national)	1A
Morrison-Saunders and Pope, 2013, Australia^17^	To present a framework for understanding and managing both process and substantive trade-offs within each step of a typical sustainability assessment process	8		NG	Universal	Project, Policy (local/regional), Policy (national), System	2
Morseletto, 2022, Netherlands^111^	To examine 7 widely applicable principles aimed at explicit environmental sustainability	7		NO (E)	Universal	Policy (local/regional), Policy (national), Policy (international), Organization	1A
Naboni and Havinga L, 2019, EU^112^	To challenge thinking on built environment design and enable designers to begin to change not only their design processes and outcomes but also their consideration of appropriate and ambitious goals for the design of our cities, buildings and spaces	3	H NG	* NG	Universal	Building, Project, City, Policy (local/regional), Policy (national), Policy (international), Organization, System	2
Nathwani and Narveson, 1995, Canada^52^	To propose principles and a general framework of reasoning for managing risk in the public interest	4		S NG	Universal	Project, Policy (local/regional), Policy (national), Policy (international)	3A
National Housing Supply and Affordability Council, 2024, Australia^113^	To present an overview of the state and functioning of Australia’s housing system	4		C NG	Australia	Project, Policy (local/regional), Policy (national), System	1A
Natural Capital Committee, 2019, UK^22^	To provide advice to government on net environmental gain	6		E NG	UK	Project, Policy (local/regional), Policy (national), System	1B
Net Positive Project, 2019, NR^114^	To provide guidance on the principles and aspirations shaping net positive strategies	6		* NG	Universal	Organization, System	1B
Nisbet and João, 2022, UK^115^	To present a framework for evaluating the quality of enhancement of positive impacts in Environmental Impact Assessment reports	8		NG	Universal	Project, Policy (local/regional), Policy (national), Policy (international)	2
Norris, 2017, NR^116^	Unstated	6		* NG	Universal	Organization, System	3B
Norris *et al.*, 2021, US^117^	To improve the scientific basis for transformative environmental, social, and economic positive changes	6		* NG	Universal	Organization, System	3A
Novak *et al.*, 2017, US^118^	To explore an alternative way of thinking that reframes the issues from problems of “what is” to the potential of “what could be” in the context of socioecological resilience	6		* NG	Universal	Project, City, System	2
O’Neill, 2020, UK^119^	To argue that significant goods that matter to people’s well-being will be lost through a policy of NNL	7		NNL (E)	Universal	Project, Policy (local/regional), Policy (national), Policy (international), Organization	3B
O’Neill, 2023, UK^120^	To address the commodification of biodiversity, and in particular with the development of offset markets as a means to achieving “NNL” or “net gains” in biodiversity, and place the commodification of biodiversity in the context of wider debates about the commodification of environmental goods	8		NO (E)	Universal	Project, Policy (local/regional), Policy (national), Policy (international)	3A
Parliamentary Office of Science and Technology (POST), 2019, UK^121^	POSTbriefs are responsive policy briefings from the Parliamentary Office of Science and Technology	2	BW NWO+	B NO	UK	Project, Policy (local/regional), Policy (national), Policy (international), System	3B
Pearl and Oliver, 2015, Canada^122^	To elaborate the mining processes that can produce net-positive development and transformational change	6		* NG	Universal	Building, Project, City, System	2
Pienkowski, 2022, UK^123^	To provide evidence of overlooked connections between nature, its conservation, and mental health	2	BW NWO	B NO	Universal	Project	1B
Place Alliance, 2020, UK^124^	To evaluate the design of 142 large-scale housing-led development projects across England against seventeen design considerations and establish a new baseline from which to measure progress on housing design quality in the future	6		* NG	UK	Building, Project, Organization	1A
Plaut *et al.*, 2012, US^125^	To outline the context, development and future direction for the LENSES Framework	3		H* NG	Universal	Building, Project, System	1A
Public Health England (PHE), 2019, UK^48^	To provide evidence-based advice on the most effective practical actions to reduce air pollution and its impact on our health	1	H NG	H NG	UK	Project, Policy (local/regional), Policy (national)	1A
Puig *et al.*, 2022, Spain^126^	To address some cultural implicit stances of environmental assessment and bring attention to their disregard for environmental values, which should guide environmental assessment towards increased levels of sustainability	6		E NNL	Universal	Project, Policy (local/regional), Policy (national), Policy (international)	1A
Rees, 1995, Canada^127^	To assess the role of cumulative assessment in the context of global change	6		E NNL	Universal	Project, Policy (local/regional), Policy (national), Policy (international), System	1A
Robinson and Cole, 2015, Canada^33^	To delineate the concept of “regenerative sustainability” – a net-positive approach to sustainability that is rooted in the notion of ‘procedural sustainability’	7		NG	Universal	Building, Project, City, System	2
Sala Benites, 2023, Australia^32^	To develop a framework to support the positive impact-based transition of existing urban precincts and neighborhoods	6		* NG	Universal	Building, Project, City, Policy (local/regional), Policy (national), Policy (international), Organization, System	3B
Sarkki *et al.*, 2023, Finland^128^	To propose a scenario skeleton titled “Rights for Life” to achieve ambitious biodiversity targets in a socially-equitable ways	8		NO	Universal	Policy (local/regional), Policy (national), Policy (international)	2
SGS Economics and Planning Pty Ltd, 2023, Australia^129^	To address “what a Plan for Victoria should include and how the planning vision could be delivered, with local government acting as a valued and indispensable partner”	4		C NG	Australia	Project, Policy (local/regional)	1B
Shoemaker, 1994, Canada^130^	To suggest a set of principles for the design of cumulative environmental assessment	4		C NG	Canada	Project, Policy (local/regional)	3B
Spiller, 2023, Australia^5^	To explore the reasons for the continuing controversy surrounding how the idea of net community benefit might be applied in the planning system and propose some ways forward	4		C NG	Australia	Project, Policy (local/regional)	3A
Stewart-Evans *et al.*, 2022, UK^131^	To outline conceptual, methodological and practical considerations on health net gain	1	H NG	H NG	Universal	Project, Policy (local/regional), Policy (national), System	1A
Stewart-Evans *et al.*, 2023, UK^50^	Unstated	1	H NG	H NG	Universal	Project, Policy (local/regional), Policy (national)	1A
Stewart-Evans *et al.*, 2024, UK^49^	To explore how a health net-gain objective could be applied in spatial planning policy and practice to improve people’s health and well-being	1	H NG	H NG	Universal	Project, Policy (local/regional), Policy (national), System	1A
Stewart-Evans *et al.*, 2024, UK^55^	To review the body of knowledge on net gain and no net loss (net-outcome) objectives and approaches applicable to health in spatial planning and development policies and practice	1	H NG	H NG	Universal	Building, Project, City, Policy (local/regional), Policy (national), Policy (international), Organization, System	1A
Stratford *et al.*, 2022, Australia^132^	Unstated	6		* NG	Universal	System	1A
Swangjang and Cumkhett, 2021, Thailand^133^	Not stated	8		NO	Universal	Project	2
Tàbara, 2023, Germany^134^	To present a conceptual model to help explore the theoretical possibilities for creating regenerative sustainability pathways	6		* NG	Universal	System	1B
Ten Kate *et al.*, 2018, UK^20^	Not stated	5	BS NWO+	B NNL	Universal	Project, Organization	2
Truman, 2019, UK^135^	To look at environmentnal net gain in practice using 2 technical topics as examples and also discuss potential challenges in implementing environmental more broadly	6		E NG	UK	Project, Policy (local/regional), Policy (national)	1A
Tuomisto and Pekkanen, 2005, Finland^136^	Unstated	1	H NG	H NG	Universal	Policy (local/regional), Policy (national), Policy (international)	3A
Tupala *et al.*, 2022, Finland^137^	To present the current understanding of the social impacts on biodiversity offsetting based on scientific literature	2	BW NWO	B NNL	Universal	Project, Policy (local/regional), Policy (national), Policy (international), Organization	1A
Velasco Fuentes, 2016, Canada^138^	To develop greater understanding to the role of ownership in the built environment, and particularly in the design of regenerative building systems and the advance towards regenerative sustainability in social-ecological systems	6		* NG	Universal	Building, Project, City, System	2
Victorian Auditor-General, 2017, Australia^139^	To assess whether Department of Environment, Land, Water and Planning and councils are managing and implementing the planning system to support the Act [Planning and Environment Act 1987] and the desired outcomes of state planning policies	4		C NG	Australia	Project, Policy (local/regional)	3B
Victoria State Government, 2022, Australia^140^	To guide a strategic assessment under Minister’s Direction No. 11	4		C NG	Australia	Policy (local/regional)	3B
Villarroya *et al.*, 2014, Spain^141^	To review the main scientific bibliography addressing the rationale behind ecological compensation in order to examine general guidelines	7		NO	Universal	Project	2
Wessel, 2016, US^142^	To present a proposal for accomplishing development that is truly sustainable	6		* NG	Universal	Project, Organization	3B
Williams, 2012, UK^143^	To explore the opportunities and challenges for the advocated changes in the way society conceptualizes the built environment	6		E NG	Universal	Building, Project	1A
Wirral Council, 2022, UK^144^	To set out the strategy, policies and proposals for meeting the Borough’s development needs in a sustainable and transformational manner	4		S NG	UK	Building, Project, Policy (local/regional)	3B
Young, 2022, Australia^145^	To present an international framework for the delivery of socially and environmentally responsible ecological restoration after mining	3		W* NG	Universal	Project, Organization	3B
Zarsky and Stanley, 2013, US^4^	To develops a framework to evaluate net benefits from mining and use it to assess the Marlin mine in Guatemala	6		* NG	Universal	Project	3B
Zhang *et al.*, 2015, China^34^	To discuss a special volume that focuses upon various dimensions of regenerative sustainability (eg, regenerative design, regenerative development, and positive development) applied to the urban built environment	6		* NG	Universal	Building, Project, City, System	1A
Zingoni de Baro, 2022, NR^37^	To set out the major features of 2 social-ecological design approaches to the built environment, Biophilic urbanism and Regenerative development	6		* NG	Universal	Building, Project, City, System	2
zu Ermgassen *et al.*, 2022, UK^146^	To examine the emerging trend of ‘nature-positive’ in business and large organizations	2	NW NWO+	N NG	Universal	Organization	1B

NR, not reported.

Targets: *, multi-objective; B, biodiversity; C, community; E, environmental; H, health; N, nature; S, social; W, well-being.

Outcomes: DNH+, do no harm or better off; NG, net gain; NNL, no net loss; NO, net outcome; NWO, no worse off; NWO+, no worse off or better off.

Net-outcome level emphasized: 1A, overarching requirement; 1B, overarching requirement (locally defined); 2, enhanced process; 3A, assessed outcome; 3B, assessed outcome (locally defined).
